# Systemic Glucose Homeostasis Requires Pancreatic but Not Neuronal ATP-sensitive Potassium Channels

**DOI:** 10.1093/function/zqaf002

**Published:** 2025-01-14

**Authors:** Athena H Li, Wen-Sheng Tsai, Wen-Hao Tsai, Shi-Bing Yang

**Affiliations:** Institute of Biomedical Sciences, Academia Sinica, Taipei 115, Taiwan; Taiwan International Graduate Program in Interdisciplinary Neuroscience, National Yang Ming Chiao Tung University and Academia Sinica, Taipei 115, Taiwan; Institute of Biomedical Sciences, Academia Sinica, Taipei 115, Taiwan; Institute of Biomedical Sciences, Academia Sinica, Taipei 115, Taiwan; Institute of Biomedical Sciences, Academia Sinica, Taipei 115, Taiwan; Neuroscience Program of Academia Sinica, Academia Sinica, Taipei 115, Taiwan

**Keywords:** K_ATP_ channels, potassium channels, glucose-stimulated insulin secretion, pancreatic β cell, mouse genetics, insulin

## Abstract

The adenosine triphosphate (ATP)-sensitive potassium (K_ATP_) channels, composed of Kir6.2 and sulfonylurea receptor 1 (SUR1) subunits, are essential for glucose homeostasis. While the role of pancreatic K_ATP_ channels in regulating insulin secretion is well-documented, the specific contributions of neuronal K_ATP_ channels remain unclear due to challenges in precisely targeting neuronal subpopulations. In this study, we utilized a Kir6.2 conditional knockout mouse model to distinguish the roles of K_ATP_ channels in different cell types. Our findings demonstrate that deletion of neuronal K_ATP_ channels does not impair glucose homeostasis, as glucose-sensing neurons retained their responsiveness despite the absence of functional K_ATP_ channels. In contrast, the deletion of K_ATP_ channels in pancreatic β cells led to significant hyperglycemia and glucose intolerance, indicating unstable blood glucose levels under varying physiological conditions. Importantly, we showed that restoring K_ATP_ channel function exclusively in pancreatic β cells within a global Kir6.2 knockout background effectively reversed glucose regulation defects. This underscores the critical role of pancreatic K_ATP_ channels in maintaining systemic glucose homeostasis. Our results challenge the previous hypothesis that neuronal K_ATP_ channels are essential for glucose regulation, suggesting that their primary function may be neuroprotective rather than homeostatic. These findings highlight pancreatic K_ATP_ channels as key regulators of glucose balance and potential therapeutic targets for correcting glucose dysregulation.

## Graphical Abstract

**Graphical Abstract:**
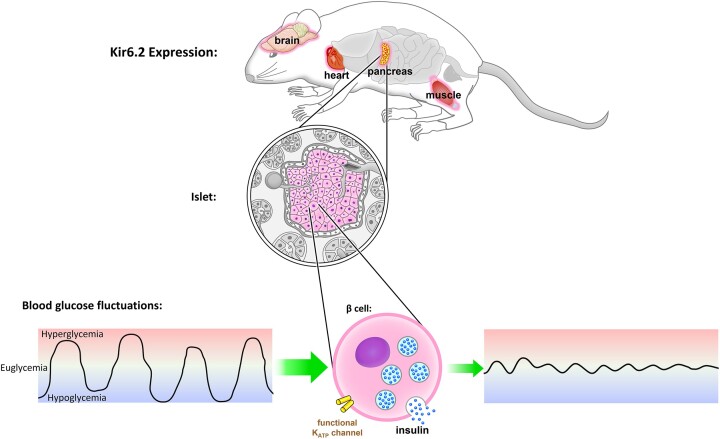
Although expressed also in the brain, heart, and muscle, only the K_ATP_ channels expressed in pancreatic β cells are essential for glucose homeostasis regulation and serve as the systemic glucostat to maintain stable circulating glucose levels.

## Introduction

Tight regulation of glucose homeostasis is essential for maintaining proper physiological functions. The endocrine and nervous systems work synergistically to ensure adequate glucose balance. Elevated circulating glucose levels stimulate insulin secretion from pancreatic β cells in a process known as glucose-stimulated insulin secretion (GSIS). At the molecular level, the ATP-sensitive potassium (K_ATP_) channel plays a central role in GSIS, acting as an “electrochemical transducer” that converts metabolic signals from glucose metabolism into membrane excitability, thereby regulating insulin secretion.^1–3^ The pancreatic K_ATP_ channel comprises four pore-forming inwardly-rectifying potassium channel (Kir6.2) and four sulfonylurea receptor (SUR1) subunits.^4,5^ Mutations in either subunit can lead to severe conditions such as permanent neonatal diabetes and persistent hyperinsulinemic hypoglycemia of infancy, further highlighting the importance of K_ATP_ channels in glucose homeostasis.^6–11^

Besides the pancreas, K_ATP_ channels are also expressed in the central nervous system (CNS), heart, and skeletal muscle. The CNS has long been considered a crucial regulator of glucose balance, as lesions in the hypothalamus and brainstem can alter glucose metabolism and body weight,^12^ and only the K_ATP_ channels expressed in the CNS but not myocytes share the same composition as that in the pancreas. Recent studies suggest that neurons in these brain areas can modulate circulating glucose levels by regulating central K_ATP_ channel activities in response to nutrients and glucostatic hormones such as leptin and insulin.^13–15^ This adequate glucose homeostasis can be disrupted through pharmacological or genetic manipulation of KATP channels. Intracerebroventricular (ICV) injection of K_ATP_ channel blocker glibenclamide could induce feeding in both fasted and fed rats, while the channel activator diazoxide could inhibit feeding.^16^ In hyperphagic mice, the blockade of K_ATP_ channels in pro-opiomelanocortin (POMC)-expressing neurons, which suppress feeding behavior when activated,^17^ was sufficient to alleviate the behavior.^13^ The ablation of K_ATP_ channels in agouti-related peptide (AgRP)-expressing neurons, a hypothalamic neuronal subpopulation that induces voracious feeding behavior when activated and becomes immediately silenced upon the detection of food,^18^ disrupted the leptin-induced inhibition of food intake via presynaptic GABAergic afferents.^19^ However, the precise mechanisms remain unclear, largely due to the complexity of neuronal populations and the lack of cell-type-specific K_ATP_ channel deletions.

In this study, we sought to clarify the role of cell-type-specific K_ATP_ channel deletion in glucose homeostasis, specifically using a Kir6.2 conditional knockout (KO) (Kir6.2^flox^) mouse model that allowed us to differentiate the effects of Kir6.2 deletion in pancreatic β cells from those in specific neuronal populations. As Kir6.2 subunits are capable of forming partially functional channels with weak ATP inhibition that give rise to proper conductance^20,21^ and SUR1 subunits are also known to form nonselective cation channels with TRPM4 subunits in the CNS that facilitate sodium (Na^+^) influx and depolarization as opposed to the hyperpolarizing effect of K_ATP_ channels due to potassium (K^+^) efflux,^22^ we considered Kir6.2 a better candidate than SUR1 for precise and complete targeting of K_ATP_ channels. Our findings indicate that mice with pancreatic β cell-specific, but not neuronal-specific K_ATP_ channel deletion, impaired glucose homeostasis. These results strongly suggest that pancreatic K_ATP_ channels, rather than neuronal K_ATP_ channels, are essential for maintaining glucose balance.

## Materials and Methods

### Animal Care

Experimental procedures were performed strictly in accordance with recommendations from the Guide for the Care and Use of Laboratory Animals of the US National Research Council. The experimental protocols were approved by the Institutional Animal Care and Use Committee of Academia Sinica under protocol number: 15-01-813.

Mice aged 3–24 weeks were housed 3–5 animals per cage in a special pathogen-free animal facility on a standard 12-hour light/dark cycle (light on from 8 am to 8 pm) and fed with a regular chow diet (PicoLab^®^ Rodent Diet 20 #5053, LabDiet, St. Louis, MO, USA) unless specified. Metabolic assays were conducted at 6–12 weeks old. Mice were euthanized after metabolic analyses and tissue samples were collected for histological analyses.

### Mouse Strains

The Kir6.2 conditional KO mouse strain, referred to as “Kir6.2^flox^,” was derived from the Kir6.2-FloxA strain (EMMA ID: EM:08 845; C57BL/6N-*Kcnj11*^tm1a(EUCOMM)Wtsi/H^), as shown in Supplementary Figure S1A, provided by the European Mouse Mutant Archive (MRC Harwell, UK). The Kir6.2-FloxA strain was initially designed as a knockin-KO, where inserting a *frt*-flanked cassette containing a neomycin resistance (Neo) cassette and β-galactosidase gene disrupted Kir6.2 function. To reinstate a functional Kir6.2 allele, we generated the Kir6.2^flox^ strain by crossing the Kir6.2-FloxA strain with a Flp deleter strain, Actin-Flp (JAX Strain No. 005 703; B6.Cg-Tg(ACTFLPe)9205Dym/J),^23^ which excised the inserted Neo cassette and β-galactosidase gene. Various Cre lines, including Nestin-Cre (JAX Strain No. 003 771; B6.Cg-Tg(Nes-cre)1Kln/J),^24^ POMC-Cre (JAX Strain No. 010 714; B6.FVB-Tg(POMC-cre)1Lowl/J),^25^ Agrp-Ires-Cre (JAX Strain No. 012 899; STOCK *Agrp^tm1(^*^cre^*^)Lowl^*/J),^26^ LepR-Cre (JAX Strain No. 032457; B6.129-*Lepr*^*tm3(cre)Mgmj*^/J),^27^ Sf1-Cre (JAX Strain No. 012 462; STOCK Tg(Nr5a1-cre)7Lowl/J),^28^ αMHC-MerCreMer (JAX Strain No. 005 657; B6.FVB(129)-*A1cf ^Tg(Myh6-^*^*cre*^^/Esr1*)1Jmk^/J),^29^ MCK-Cre (JAX Strain No. 006 475; B6.FVB(129S4)-Tg(Ckmm-cre)5Khn/J),^30^ TH-Cre (JAX Strain No. 008 601; B6.Cg-*7630403G23Rik^Tg(Th-^*^cre^*^)1Tmd^*/J),^31^ Protamine-Cre (JAX Strain No. 007 252; B6Ei.129S4-Tg(Prm-cre)58Og/EiJ),^32^ and Ai14 (JAX Strain No. 007 914; B6.Cg-*Gt(ROSA)26Sor^tm14(CAG-tdTomato)Hze^*/J),^33^ were from The Jackson Laboratory (Bar Harbor, USA). Additionally, the Ins1-RFP-Cre strain (RMRC No. RMRC13172; C57BL/6-Tg(Ins1-RFP,-cre)14Narl) was obtained from the National Laboratory Animal Center (NLAC) Rodent Mouse Resource Center (RMRC) in Taipei, Taiwan. All strains were backcrossed and maintained on a C57BL/6J background for more than 15 generations to ensure genetic consistency.

In our experimental setup, heterozygous littermates (X^Cre/0^; Kir6.2^flox/+^) were used as controls to eliminate any effects specific to the Cre lines.^34^ To validate Cre recombinase activity in the experimental mice, each mouse carried the tdTomato reporter allele (Ai14), allowing visualization of Cre expression. Mice exhibiting off-target tdTomato expression were excluded from the study to ensure precise targeting of Cre activity. Additionally, the genetic deletion of Kir6.2 was further confirmed by in situ hybridization (RNAscope^®^), verifying the absence of Kir6.2 expression in targeted tissues and cell types.

### Histological Analyses

All mice were euthanized by intraperitoneal injections of 25 mg/kg tiletamine, 25 mg/kg zolazepam (Zoletil^®^, Virbac, Carros, France), and 5.84 mg/kg xylazine (Rompun^®^, Bayer, Germany) mixture and perfused with 1 × phosphate-buffered saline (1 × PBS; 10 mm, pH 7.4) and 4% paraformaldehyde (PFA; Sigma-Aldrich, St. Louis, USA) in 1 × PBS (4% PFA/PBS; pH 7.4). Harvested tissue samples were fixed with 4% PFA/PBS at 4°C overnight, cryoprotected in 30% sucrose (J.T.Baker, Phillipsburg, USA)/PBS at 4°C, embedded in Cryo-Gel (Leica, Germany), cryosectioned to 14–20 µm on a Leica CM3050 S Research Cryostat, and directly mounted onto glass slides with hydrophobic coating (Matsunami APS^™^, Japan).

For RNAscope^®^ (ACDBio, Newark, USA) 2.5 HD Duplex Assay, tissue sections were hybridized with the following probes: Mm-Kcnj11-O1, Mm-Agrp-C2, and Mm-Gcg-C2, counterstained with hematoxylin, air-dried on a slide warmer at 60°C, and mounted with xylene-free, non-aqueous VectaMount^®^ Mounting Medium H-5000 (Vector Laboratories, Newark, USA).

Bright-field and confocal images were acquired, respectively, using the Olympus BX51 microscope (Tokyo, Japan) and the Zeiss LSM 700 laser scanning confocal microscope (Zeiss, Germany).

### Metabolic Analyses

Monthly measurements of mouse body weight were taken from 1 to 5 months. Blood glucose levels of the mice were measured via lateral tail veins using a blood glucose monitoring meter (OneTouch^®^ Ultra Plus Flex^™^ Meter).

Protocols for intraperitoneal (i.p.) glucose tolerance test (IPGTT), insulin tolerance test (ITT), and 2-deoxy-glucose (2DG)-induced glycopenia assessment were designed based on previous studies.^35,36^ The mice were fasted for 6 (IPGTT and 2DG assay) or 4 hours (ITT) with free access to water and were injected intraperitoneally with 2 g/kg glucose (Merck, Rahway, USA) dissolved in double-distilled water (ddH_2_O) (IPGTT), 0.75 U/kg bovine insulin (Sigma-Aldrich) dissolved in saline containing 0.5% bovine serum albumin (Sigma-Aldrich) to avoid absorption to Eppendorf tubes and 10 mm hydrochloric acid (J.T. Baker, USA) to facilitate the dissolution of insulin (ITT), and 500 mg/kg 2DG (Sigma-Aldrich) dissolved in ddH_2_O (2DG assay) with blood glucose levels measured at designated time points.

For 3-stage blood glucose level monitoring experiments, mice were maintained on a 12-hour light/dark cycle. The 3-stage monitoring was divided into the 24-hour *ad libitum* stage, the 24-hour fasting stage, and the 4-hour re-feeding stage. The mice were provided with free access to the regular chow diet and water during the *ad libitum* stage, then moved to clean cages without access to chow pellets for the fasting stage, and, finally, reintroduced to free access to the chow pellets during the re-feeding stage, with blood glucose levels measured at designated time points (*ad libitum*: every 4 hours; fasting: every 2 hours for the former 12 hours, every 4 hours for the latter 12 hours; re-feeding: every 0.5 hour).

### Plasma Insulin and Glucagon Measurements

The blood samples were collected via the lateral tail vein using K^+^ EDTA-coated microvettes (Microvette^®^ CB 300 K2 EDTA, Sarstedt, Germany) at the following time points: (1) after a 6-hour fasting duration and 20 minutes after an acute hyperglycemia induction (i.p. 2 g/kg glucose injection), (2) after a 24-hour fasting duration and 30 minutes after re-feeding, (3) after a 6-hour fasting duration and 30 minutes after a 2DG injection (i.p. 500 mg/kg). The blood samples were left on ice prior to centrifugation at 3000 rpm, 4°C in a refrigerated microcentrifuge (Hermle Z216MK, Germany). The plasma samples were transferred to new Eppendorf tubes and preserved at −80°C until insulin or glucagon level measurements using enzyme-linked immunoabsorbent assay (ELISA) (Mouse Ultrasensitive Insulin ELISA, ALPCO, Salem, USA; Glucagon ELISA–10 µL, Mercodia, Uppsala, Sweden). The absorbance of each sample was measured using the spectrophotometer (SpectraMax^®^ 190 Microplate Reader, Molecular Devices, San Jose, USA). Concentrations were determined as recommended by the manufacturer.

### Intraductal Viral Administration

The adeno-assoociated virus (AAV) vectors AAV9-FLEX-Kir6.2-GFP and AAV9-GFP generated by the AAV core facility at Academia Sinica were injected into the pancreas of Ins1^Cre/0^; Kir6.2 KO mice aged 6-7 weeks old via pancreatic ductal infusion^37^ (Supplementary Figure S1B). The injected total viral genome (vg) was adjusted to 2 × 10^11^ vg per mouse and diluted with RPMI 1640 cell culture medium (Thermo Fisher Scientific, Waltham, USA). After recovery from surgery, IPGTT was retested, the plasma was collected after all IPGTT trials, and injected mice were sacrificed for electrophysiology recordings and validation of viral infection efficiency.

### Electrophysiology

Mouse pancreatic (140 µm) and brain slices (300 µm), sectioned with a 5100mz series vibrating microtome (Campden Instruments Ltd., Leicestershire, UK), were prepared as previously described.^38,39^ Glass pipettes were pulled from 1.5-mm borosilicate glass capillaries (Sutter Inc., Novato, USA). The MultiClamp 700B Microelectrode Amplifier (Axon Instruments^®^ Molecular Devices, San Jose, USA) was used for recordings, and data were acquired at 10 kHz using the Axon^™^ Digidata^®^ 1550A Data Acquisition System (Axon Instruments^®^ Molecular Devices, USA).

For pancreatic slice recordings, the mouse pancreas was inflated via ductal injection of 1.9% low melting point agarose (FocusBio, Spain) dissolved in the extracellular solution containing: 125 mm NaCl, 5 mm KCl, 26 mm NaHCO_3_, 1.25 mm NaH_2_PO_4_, 2 mm sodium pyruvate, 3 mm myo-inositol, 0.25 mm L-ascorbic acid, 6 mm lactic acid, 2 mm CaCl_2_, 1 mm MgCl_2_, 3 mm D(+)-glucose, sectioned, and submerged in ice-cold incubating solution containing: 125 mm NaCl, 5 mm KCl, 10 mm HEPES, 10 mm NaHCO_3_, 1.25 mm NaH_2_PO_4_, 2 mm sodium pyruvate, 3 mm myo-inositol, 0.25 mm L-ascorbic acid, 6 mm lactic acid, 2 mm CaCl_2_, 1 mm MgCl_2_, 3 mm D(+)-glucose with continuous carbogen (95% O_2_ and 5% CO_2_) bubbling until recording. The intracellular solution contained 15 mm KCl, 135 mm K^+^ gluconate, 10 mm creatine phosphate, 10 mm HEPES, 1 mm MgCl_2_, 5 mm EGTA, 4 mm MgATP, and 1 mm Na_2_GTP. The recording chamber was perfused with the extracellular solution or that containing 10 µm glibenclamide.

For brain slice recordings (Supplementary Figure S1C), mice were anesthetized with isoflurane, perfused with ice-cold cutting solution containing 110 mm choline chloride, 2.5 mm KCl, 25 mm NaHCO_3_, 1.25 mm NaH_2_PO_4_, 3.1 mm sodium pyruvate, 11.6 mm (+)-sodium L-ascorbate, 0.5 mm CaCl_2_, 7 mm MgCl_2_, 11 mm D(+)-glucose, and 12 mm N-acetyl cysteine, and decapitated. Harvested mouse brains were immersed in ice-cold cutting solution with continuous carbogen bubbling and sectioned.^40^ After 30 minutes of incubation with continuous bubbling, the brain slices were transferred to ice-cold carbogen-equilibrated artificial cerebrospinal fluid (aCSF) solution containing: 119 mm NaCl, 2.5 mm KCl, 26.2 mm NaHCO_3_, 1 mm NaH_2_PO_4_, 1.3 mm MgSO_4_, 2.5 mm CaCl_2_, and 11 mm D(+)-glucose until recording. The intracellular solution contained 15 mm KCl, 135 mm K^+^ gluconate, 10 mm creatine phosphate, 10 mm HEPES, 1 mm MgCl_2_, 5 mm EGTA, 4 mm (for glucose responsiveness assessment) or 0.5 mm (for K_ATP_-dependent K^+^ conductance assessment) MgATP, 1 mm Na_2_GTP, and the recording chamber was perfused with aCSF solutions with different glucose concentrations: 0.5 mm, 2 mm, and 25 mm. For K_ATP_-dependent K^+^ conductance assessment, the aCSF solutions (0.5 mm glucose) contained 300 µm diazoxide or 10 µm glibenclamide.

### Statistical Analyses

Statistical analyses were performed using GraphPad Prism 10 (GraphPad Software, Boston, USA). All data are presented as the mean ± standard error of the mean (SEM). Statistical significance between groups is defined as a probability value smaller than 0.05 (*P* < 0.05) and is labeled with an asterisk (*). For data analyzed with two-way ANOVA, statistics for group factors have been provided and annotated unless specified. Data were analyzed as indicated in figure legends.

## Results

### Glucose Intolerance Is Caused Solely by the Lack of Pancreatic K_ATP_ Channels

**Figure 1.**
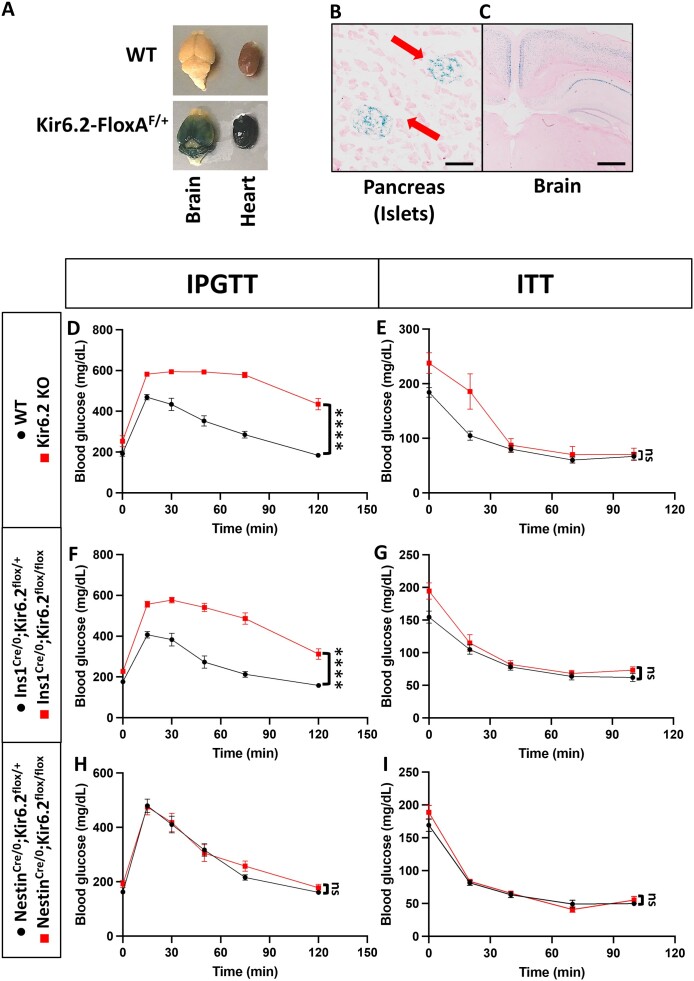
Pancreatic K_ATP_ channels are essential for regulating glucose tolerance. (A) Whole-mount X-gal staining in Kir6.2-FloxA^F/+^ mouse (*bottom*) brain and heart (from left to right). (B-C) X-gal staining in Kir6.2-FloxA^F/+^ mouse pancreas (B) and brain (C) tissue sections. Blue (dark) signals indicate Kir6.2-dependent β-galactosidase activity; nuclei were stained with Nuclear Fast Red (pink). Red arrows indicate pancreatic islets (B). Scale bars, 100 µm (B); 500 µm (C). (D-I) Blood glucose levels of WT versus (vs) Kir6.2 KO (D, E), Ins1^Cre/0^; Kir6.2^flox/+ vs flox/flox^ (F, G), and Nestin^Cre/0^; Kir6.2^flox/+ vs flox/flox^ (H, I) mice during IPGTT (D, F, H) and ITT (E, G, I). WT: *n* = 16, 17 (D-E); Kir6.2 KO: *n* = 9 (D), 8 (E); Ins1^Cre/0^; Kir6.2^flox/+^: *n* = 9 (F, G); Ins1^Cre/0^; Kir6.2^flox/flox^: *n* = 15 (F, G); Nestin^Cre/0^; Kir6.2^flox/+^: *n* = 10 (H), 11 (I); Nestin^Cre/0^; Kir6.2^flox/flox^: *n* = 8 (H, I). Statistical comparisons were made by two-way ANOVA with Šídák’s correction (D-I). Data are represented as mean ± SEM. ****P < 0.0001, ns: not significant.

We initially validated the Kir6.2 reporter mouse (Kir6.2-FloxA) through whole-mount X-gal staining. In this mouse line, a β-galactosidase gene was inserted immediately downstream of the Kir6.2 promoter, serving as a reporter to visualize the native expression pattern of *Kcnj11*, the gene encoding Kir6.2. Indeed, similar to the previous studies, the Kir6.2 expression was observed in the brain and heart (Figure 1A); stained tissue sections indicated the expression of the *Kcnj11* gene in the pancreatic islets (Figure 1B) and brain cortex (Figure 1C). Further RNAscope staining showed dense expression in the heart and skeletal muscle (Supplementary Figure S2A and B). Ubiquitous expression of the *Kcnj11* mRNA was observed in the brain (Supplementary Figure S2C), including the cortical and subcortical brain regions (Supplementary Figure S2D-G) and the hypothalamus (Supplementary Figure S2H-K). Previous studies have shown that the K_ATP_ channel KO mice, including Kir6.2^−/−^ and SUR1^−/−^ mice, exhibited impaired glucose homeostasis and GSIS.^41,42^

We generated a new whole-body Kir6.2 KO mouse by crossing Kir6.2^flox^ mice with Protamine-Cre mice, which express Cre recombinase specifically in the male germline. This cross produced offspring with a Kir6.2-null genotype, resulting in complete deletion of the *Kcnj11* gene. Unlike the previous KO model, which disrupted *Kcnj11* by inserting a neomycin-resistance cassette,^41^ this genetic design achieved a near-complete deletion of the entire Kir6.2 protein. To validate this newly generated Kir6.2 KO mouse line, we conducted comparative analyses with the original Kir6.2 KO mouse line.^41^ Both models displayed comparable glucose-related phenotypes, including glucose intolerance (two-way ANOVA with Šídák’s correction, *P* < 0.0001, F (1, 23) = 72.00) (Figure 1D) and impaired GSIS without affecting insulin sensitivity (two-way ANOVA with Šídák’s correction, *P* = 0.0155, F (1, 23) = 6.840) nor body weight (two-way ANOVA with Šídák’s correction, *P* = 0.8395, F (1, 11) = 0.04302) (Figure 1E, Supplementary Figure S4A). Additionally, we confirmed the absence of *Kcnj11* expression in the new Kir6.2 KO mice (Supplementary Figure S3), supporting the effectiveness of this model for investigating the functional impacts of Kir6.2 deletion.

To differentiate how the lack of functional neuronal or pancreatic K_ATP_ channels attributed, respectively, to the glucose dyshomeostasis phenotypes observed in Kir6.2 KO mice, the metabolic manifestations were re-examined in mice upon specific Kir6.2 deletion in the nervous system (Nestin^Cre/0^; Kir6.2^flox/flox^) or the pancreatic β cells (Ins1^Cre/0^; Kir6.2^flox/flox^) after validation of successful deletion in target regions (Supplementary Figure S3). The Ins1^Cre/0^; Kir6.2^flox/flox^ mice exhibited similar phenotypes as seen in the whole-body Kir6.2 KO mice: severely impaired glucose tolerance (two-way ANOVA with Šídák’s correction, *P* < 0.0001, F (1, 22) = 55.03) (Figure 1F) without affecting insulin sensitivity (two-way ANOVA with Šídák’s correction, *P* = 0.1627, F (1, 22) = 2.087) nor body weight (two-way ANOVA with Šídák’s correction, *P* = 0.8063, F (1, 13) = 0.6265) (Figure 1G, Supplementary Figure S4B), whereas Nestin^Cre/0^; Kir6.2^flox/flox^ mice showed no effect in either glucose tolerance (two-way ANOVA with Šídák’s correction, *P* = 0.5930, F (1, 16) = 0.2975), insulin sensitivity (two-way ANOVA with Šídák’s correction, *P* = 0.4220, F (1, 17) = 0.6770), or body weight (two-way ANOVA with Šídák’s correction, *P* = 0.7537, F (1, 11) = 0.1035) (Figure 1H and I, Supplementary Figure S4C).

**Figure 2.**
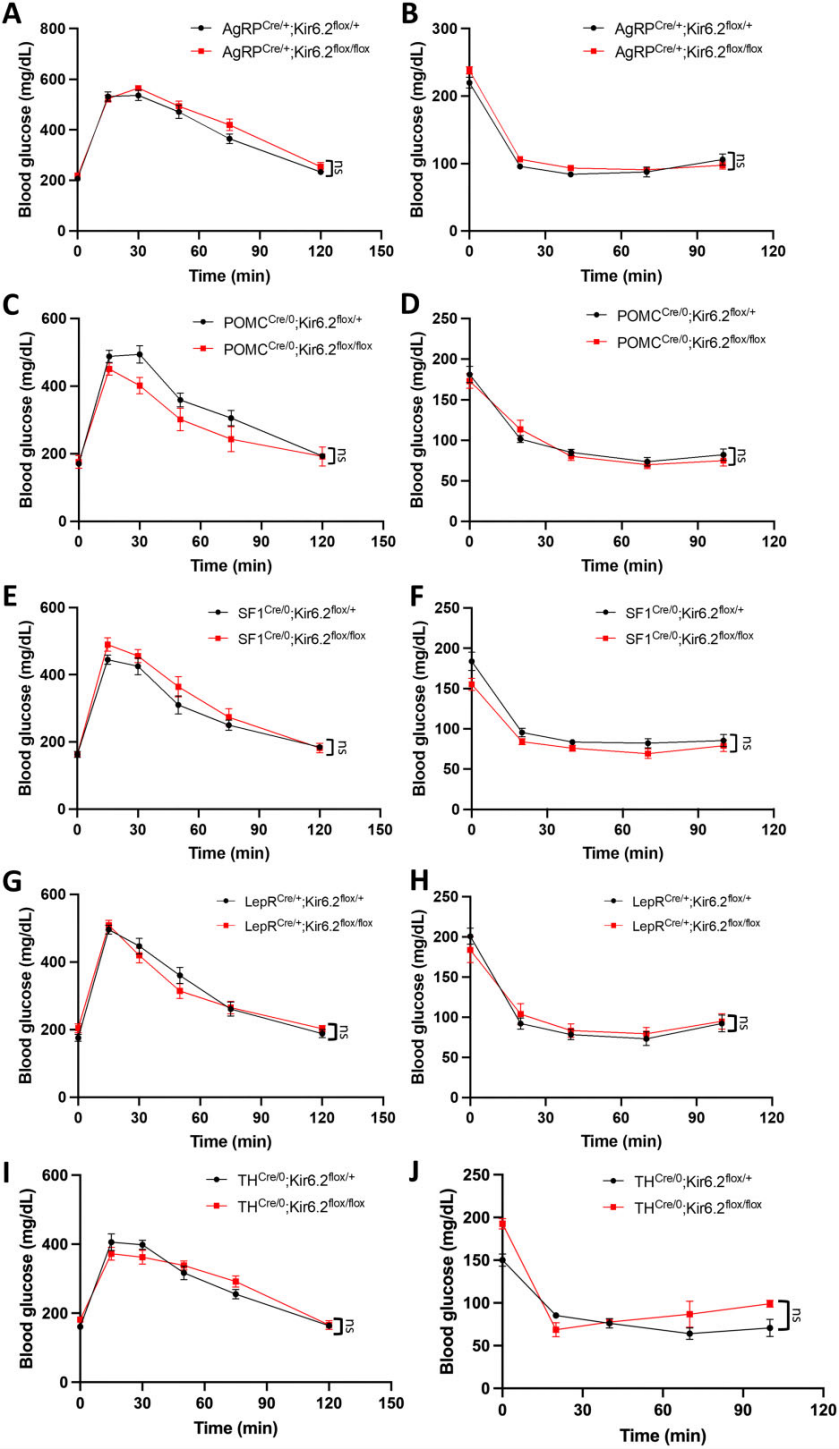
Glucose tolerance and insulin sensitivity are unaffected in mice without functional neuronal K_ATP_ channels. (A-J) Blood glucose levels during IPGTT and ITT in (A, B) AgRP^Cre/+^; Kir6.2^flox/+, flox/flox^ (IPGTT: *P* = 0.2381, F (1, 31) = 1.447; ITT: *P* = 0.2210, F (1, 31) = 1.560), (C, D) POMC^Cre/0^; Kir6.2^flox/+, flox/flox^ (IPGTT: *P* = 0.1655, F (1, 20) = 2.071; ITT: *P* = 0.7440, F (1, 22) = 0.1093), (E, F) SF1^Cre/0^; Kir6.2^flox/+, flox/flox^ (IPGTT: *P* = 0.2804, F (1, 23) = 1.222; ITT: *P* = 0.0659, F (1, 23) = 3.730), (G, H) LepR^Cre/+^; Kir6.2^flox/+, flox/flox^ (IPGTT: *P* = 0.9119, F (1, 33) = 0.01243; ITT: *P* = 0.8672, F (1, 25) = 0.02855), and (I, J) TH^Cre/0^; Kir6.2^flox/+, flox/flox^ mice (IPGTT: *P* = 0.9186, F (1, 12) = 0.01089; ITT: *P* = 0.1967, F (1, 12) = 1.869). AgRP^Cre/+^; Kir6.2^flox/+^: *n* = 13 (A, B); AgRP^Cre/+^; Kir6.2^flox/flox^: *n* = 20 (A, B); POMC^Cre/0^; Kir6.2^flox/+^: *n* = 11 (C, D); POMC^Cre/0^; Kir6.2^flox/flox^: *n* = 11 (C), 13 (D); SF1^Cre/0^; Kir6.2^flox/+^: *n* = 11 (E, F); SF1^Cre/0^; Kir6.2^flox/flox^: *n* = 14 (E, F); LepR^Cre/+^; Kir6.2^flox/+^: *n* = 17 (G), 13 (H); LepR^Cre/+^; Kir6.2^flox/flox^: *n* = 18 (G), 14 (H); TH^Cre/0^; Kir6.2^flox/+^: *n* = 11 (I, J); TH^Cre/0^; Kir6.2^flox/flox^: *n* = 3 (I, J). Statistical comparisons were made by two-way ANOVA with Šídák's correction. Data are represented as mean ± SEM. ns: not significant.

Due to potential antagonizing effects among neuronal populations, Kir6.2 was further selectively deleted in specific subsets of neurons that are suggested to be involved in K_ATP_-mediated glucostatic responses: (1) AgRP and (2) POMC neurons, both “first-order” sensory neurons in detecting humoral cues^19,43^; (3) steroidogenic factor-1 (SF1)-expressing neurons, of which artificial manipulation of its excitability had a profound effect on the blood glucose levels^44,45^; (4) leptin receptor (LepR)-expressing neurons, in which functional K_ATP_ channels are required for hypothalamic leptin-mediated glucostatic effects^13,46^; (5) tyrosine hydroxylase (TH)-expressing catecholaminergic neurons, which regulate brown adipose tissue sympathetic nerve activity via K_ATP_ channels.^47^ However, none of these Kir6.2 conditional KO mice exhibited glucose dyshomeostasis (two-way ANOVA with Šídák’s correction) (Figure 2). Specific deletion of Kir6.2 in skeletal muscle (muscle creatine kinase-expressing, MCK^+^) or heart (α-myosin heavy chain-expressing, αMHC^+^), two metabolically active organs with strong *Kcnj11* expression, did not result in any abnormalities in glucose homeostasis (two-way ANOVA with Šídák's correction) (Supplementary Figure S5).

### Neuronal K_ATP_ Channels Do Not Modulate Glycopenia-Induced Counter-regulatory Mechanisms

**Figure 3.**
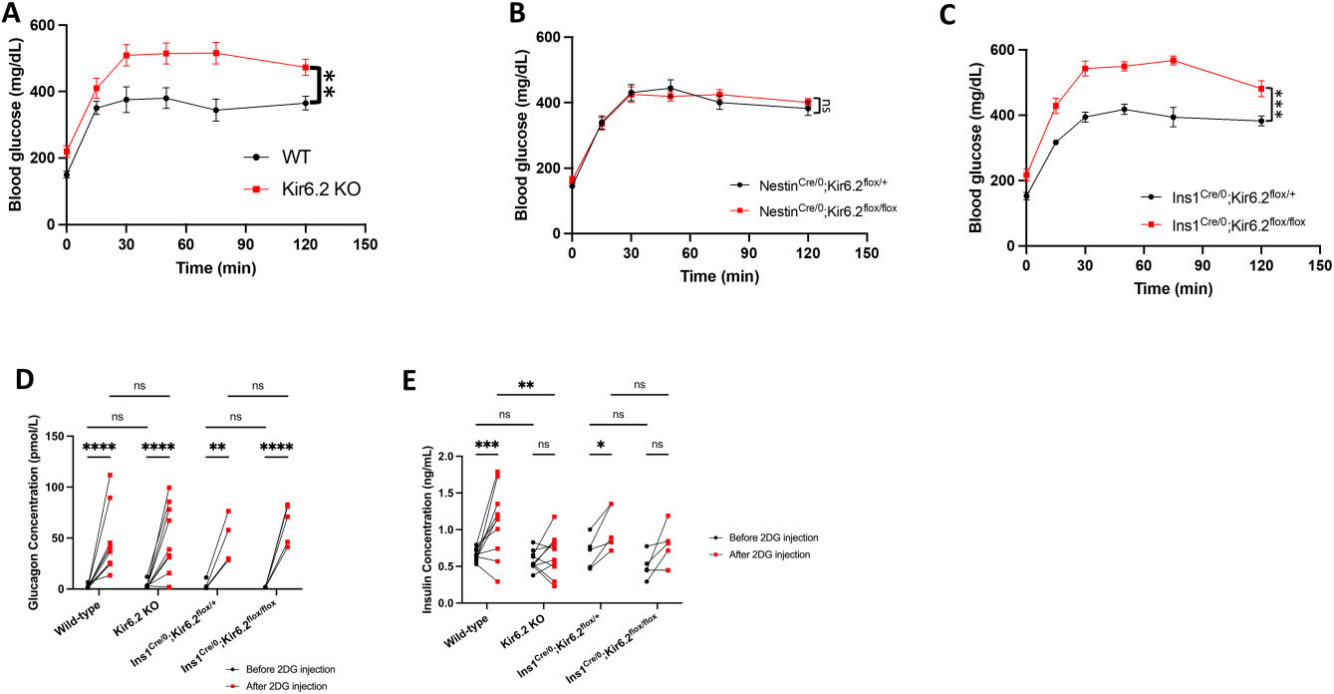
Glycopenia-induced counter-regulatory mechanisms are regulated by pancreatic but not neuronal K_ATP_ channels. (A-C) Blood glucose levels during 2DG-induced glycopenia assessment in WT vs Kir6.2 KO (A), Nestin^Cre/0^; Kir6.2^flox/+^ vs ^flox/flox^ (B), and Ins1^Cre/0^; Kir6.2^flox/+^ vs ^flox/flox^ mice (C). (D-E) Plasma glucagon (D) and insulin concentration (E) after a 6-hour fasting duration (Before 2DG injection) and 30 minutes after 2DG injection (After 2DG injection) in WT, Kir6.2 KO, Ins1^Cre/0^; Kir6.2^flox/+^, and Ins1^Cre/0^; Kir6.2^flox/flox^ mice. WT: *n* = 8 (A), 10 (D, E); Kir6.2 KO: *n* = 8 (A), 9 (D), 10 (E); Ins1^Cre/0^; Kir6.2^flox/+^: 5 (C, E), 4 (D); Ins1^Cre/0^; Kir6.2^flox/flox^: *n* = 10 (C), 5 (D), 6 (E); Nestin^Cre/0^; Kir6.2^flox/+^: *n* = 10 (B); Nestin^Cre/0^; Kir6.2^flox/flox^: *n* = 8 (B). Statistical comparisons were made by two-way ANOVA with Šídák’s correction (A-C) and two-way ANOVA with Tukey’s correction followed by multiple comparisons (D-E). Data are represented as mean ± SEM. *P < 0.05, **P < 0.01, ***P < 0.001, ****P < 0.0001, ns: not significant.

We observed that Kir6.2 KO mice displayed exacerbated 2DG-induced glycopenia (two-way ANOVA with Šídák’s correction, *P* = 0.0062, F (1, 14) = 10.37) (Figure 3A). Although it has been suggested that the absence of functional K_ATP_ channels in the autonomic nervous system—the central regulator of hypoglycemia-induced counter-regulatory mechanisms—could account for the heightened glucose levels following 2DG administration in Kir6.2 KO mice,^35,48^ we found that both the Nestin^Cre/0^; Kir6.2^flox/flox^ mice and their sibling controls had comparable blood glucose levels upon 2DG administration (two-way ANOVA with Šídák’s correction, *P* = 0.8319, F (1, 16) = 0.04658) (Figure 3B). Similar 2DG-induced glycopenia was also observed in AgRP^Cre/+^; Kir6.2^flox/flox^ (two-way ANOVA with Šídák’s correction, *P* = 0.400, F (1, 21) = 0.7379) and TH^Cre/0^; Kir6.2^flox/flox^ (two-way ANOVA with Šídák’s correction, *P* = 0.5466, F (1, 12) = 0.3849) (Supplementary Figure S6), suggesting that the exacerbated glycopenic effect of 2DG in Kir6.2 KO mice is not of neuronal but of pancreatic origin (two-way ANOVA with Šídák’s correction, *P* = 0.0001, F (1, 13) = 29.59) (Figure 3C). Furthermore, we also observed that the glucagon secretion induced by 2DG was comparable across all groups (two-way ANOVA with Tukey’s correction, *P* = 0.7469, F (3, 24) = 0.4105) (Figure 3D and Supplementary Table S1). However, Kir6.2 KO and Ins1^Cre/0^; Kir6.2^flox/flox^ mice demonstrated an intensified 2DG-induced glycopenia (Figure 3A and C). Additionally, Kir6.2 KO and Ins1^Cre/0^; Kir6.2^flox/flox^ mice exhibited reduced insulin secretion (two-way ANOVA with Tukey’s correction, *P* = 0.0189, F (3, 26) = 3.960) (Figure 3E and Supplementary Table S2), which may underlie the elevated blood glucose levels observed upon 2DG administration. These findings suggest that the pancreatic K_ATP_ channel is essential for maintaining systemic glucose homeostasis.

### The Lack of Pancreatic K_ATP_ Channels in Pancreatic β Cells Causes Glucose Dyshomeostasis

**Figure 4.**
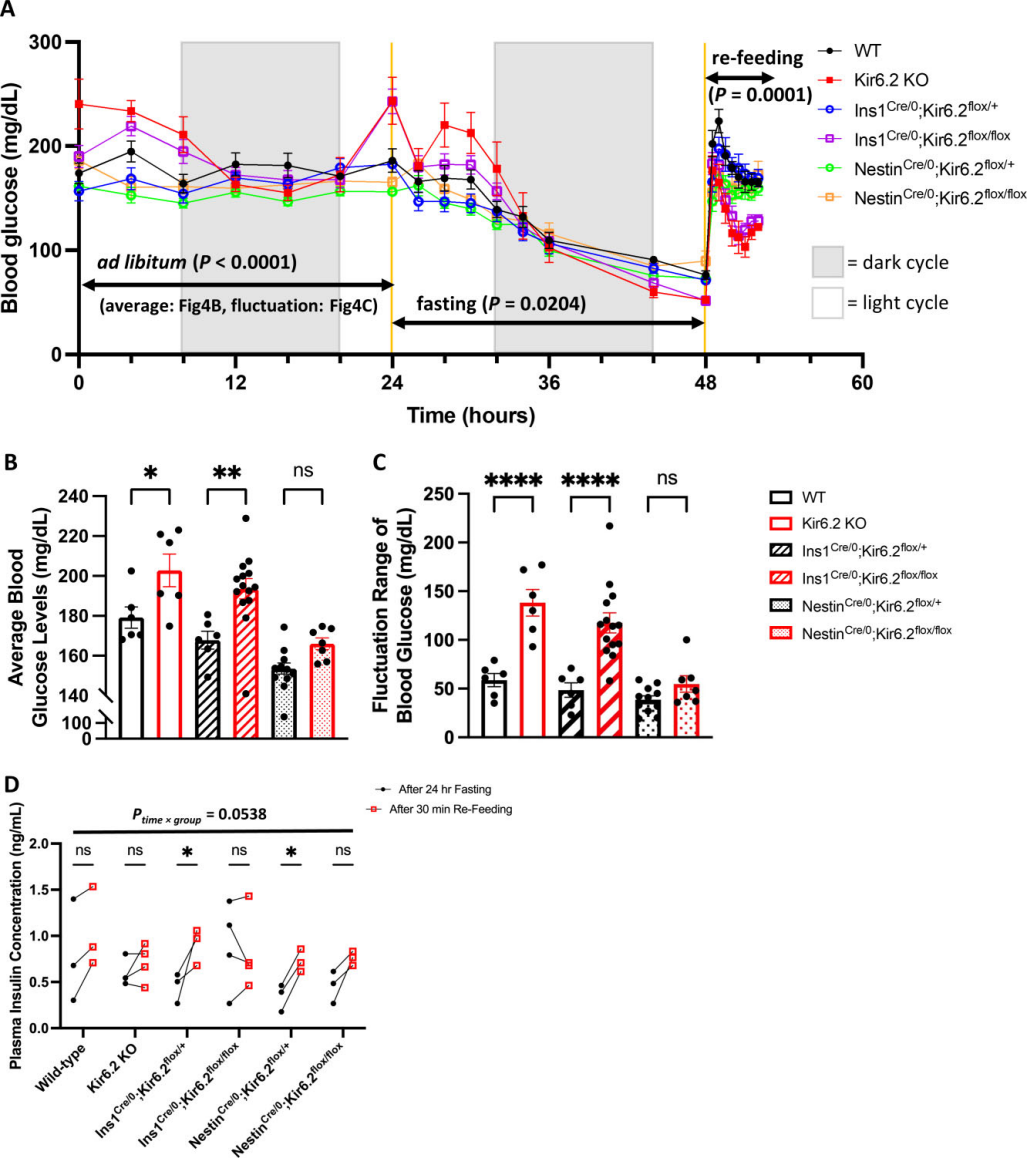
K_ATP_ channels in pancreatic β cells act as the systemic glucostat. (A) 3-stage monitoring of blood glucose levels. *Ad libitum: P* < 0.0001, F (5, 44) = 14.23; fasting: *P* = 0.0204, F (5, 44) = 3.003; re-feeding: *P* = 0.0001, F (5, 44) = 6.492; made by two-way ANOVA with Tukey’s correction. (B) The average blood glucose levels during *ad libitum*. (C) The fluctuation range of blood glucose levels during *ad libitum*, which is derived from the difference between the maximum and minimum value of individual mouse *ad libitum* blood glucose levels. (D) Plasma insulin concentration in mice after a 24-hour fasting duration and 30 minutes after re-feeding. WT: *n* = 6 (A-C), 3 (D); Kir6.2 KO: *n* = 6 (A–C), 4 (D); Ins1^Cre/0^; Kir6.2^flox/+^: *n* = 6 (A-C), 3 (D); Ins1^Cre/0^; Kir6.2^flox/flox^: *n* = 14 (A-C), 4 (D); Nestin^Cre/0^; Kir6.2^flox/+^: *n* = 11 (A-C), 3 (D); Nestin^Cre/0^; Kir6.2^flox/flox^: *n* = 7 (A-C), 3 (D). Statistical comparisons were made by two-way ANOVA with Tukey’s correction followed by multiple comparisons (A) or Šídák’s correction followed by multiple comparisons (B-D). Data are represented as mean ± SEM. *P < 0.05, **P < 0.01, ****P < 0.0001, ns: not significant.

Glucose dyshomeostasis observed in only Kir6.2 KO and Ins1^Cre/0^; Kir6.2^flox/flox^ mice suggested that pancreatic rather than neuronal K_ATP_ channels are critical to maintain the adequate glucose homeostasis, further proven in a 3-stage blood glucose monitoring (Figure 4A and Supplementary Figure S7). During the *ad libitum* state, the blood glucose levels and the fluctuation ranges were significantly higher in Kir6.2 KO (two-way ANOVA with Šídák’s correction, average: *P* = 0.0226; fluctuation range: *P* < 0.0001) and Ins1^Cre/0^; Kir6.2^flox/flox^ mice (two-way ANOVA with Šídák’s correction, average: *P* = 0.0025; fluctuation range: *P* < 0.0001) (Figure 4B and C). Although the Nestin^Cre/0^; Kir6.2^flox/flox^ mice showed constitutively higher blood glucose levels compared to their sibling controls during the *ad libitum* stage (two-way ANOVA with Šídák’s correction; *P* = 0.0097, F (1, 16) = 8.603) (Supplementary Figure S7C), the blood glucose levels showed no difference when averaged (two-way ANOVA with Šídák’s correction, *P* = 0.2363), and neither did the fluctuation range (two-way ANOVA with Šídák’s correction, *P* = 0.5382) (Figure 4B and C), indicating the possibility of neuronal K_ATP_ channels controlling the set point of blood glucose levels while glucose homeostasis remains unperturbed. Both Kir6.2 KO and Ins1^Cre/0^; Kir6.2^flox/flox^ mice failed to secrete insulin accordingly after 30 minutes of re-feeding (Figure 4D and Supplementary Table S3). These results confirm that pancreatic K_ATP_ channels are essential for maintaining adequate glucose homeostasis in response to daily metabolic challenges. During the fed *ad libitum* period, pancreatic β cells respond to minute fluctuations in blood glucose by fine-tuning insulin release through GSIS. This process highly depends on functional K_ATP_ channels, which act as metabo-electrical transducers, enabling immediate negative feedback control with high temporal accuracy. In the fasting state, lower ATP levels keep more K_ATP_ channels open, further suppressing basal insulin secretion. Upon refeeding, the rapid increase in blood glucose triggers robust insulin release via GSIS, swiftly restoring blood glucose levels to baseline (Figure 4A). These regulatory effects are absent in Kir6.2 KO and Ins1^Cre/0^; Kir6.2^flox/flox^ mice, further confirming that pancreatic K_ATP_ channels function as the systemic glucostat.

### Restoration of Functional K_ATP_ Channels in Pancreatic β Cells Is Sufficient to Restore Glucose Homeostasis in Kir6.2 KO Mice

**Figure 5.**
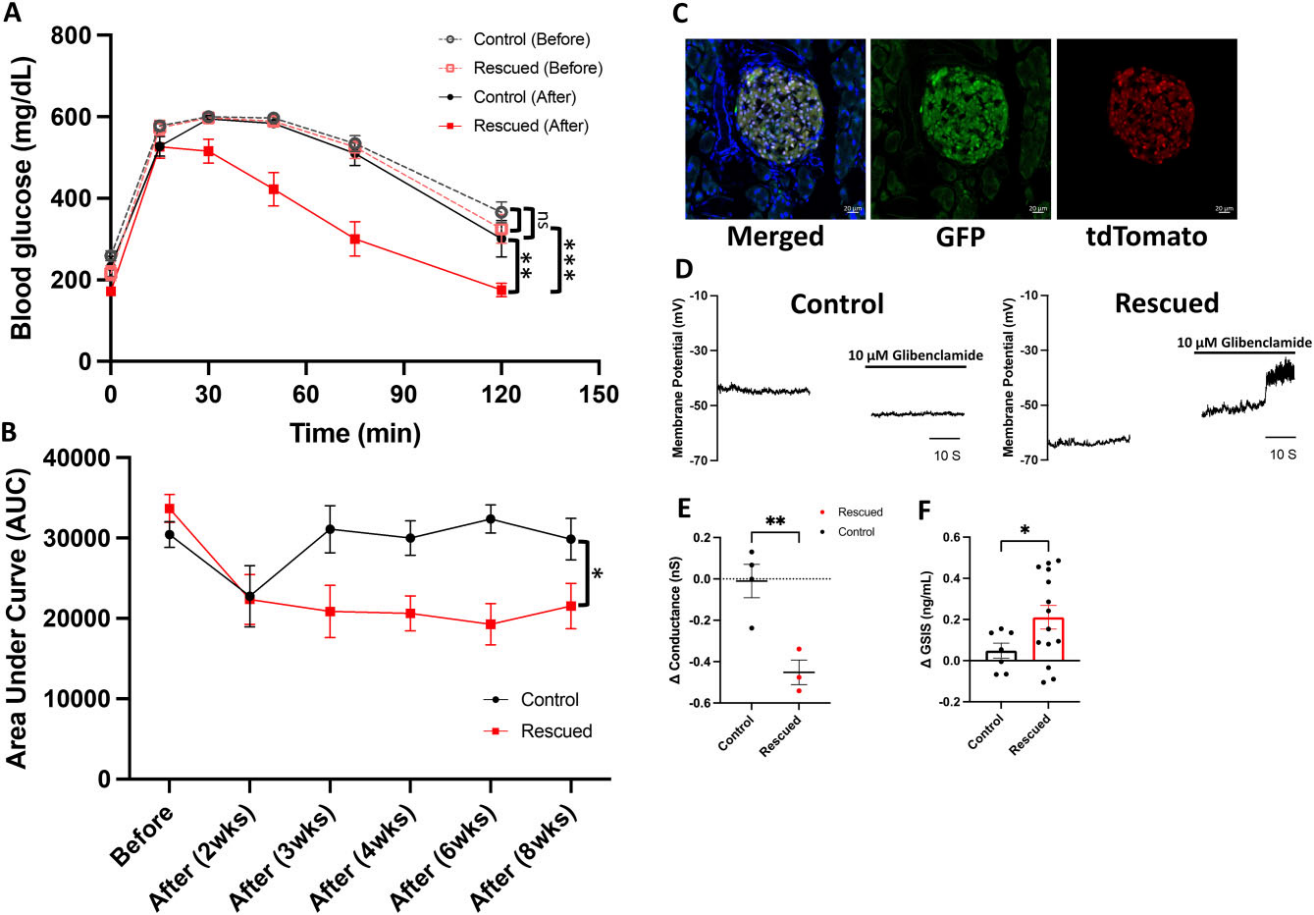
Restoration of functional pancreatic K_ATP_ channels rescues glucose homeostasis. (A) Blood glucose levels during IPGTT in control (AAV9-GFP-injected) and rescued (AAV9-FLEX-Kir6.2-GFP-injected) mice before surgery and 8 weeks after surgery. Control before vs control after: *P* = 0.1355, F (1, 16) = 2.471; rescued before vs rescued after: *P* = 0.0008, F (1, 18) = 16.24; control before vs rescued before: *P* = 0.3206, F (1, 17) = 1.047; control after vs rescued after: *P* = 0.0038, F (1, 17) = 11.20; made by two-way ANOVA with Šídák’s correction. (B) Area under curve (AUC) of IPGTT traces comparison in control and rescued mice before surgery and 2, 3, 4, 6, and 8 weeks post-surgery. (C) Fluorescent images of Ins1^Cre/0^; Ai14^+/−^; Kir6.2 KO pancreatic islets that have been successfully infected with AAV9-FLEX-Kir6.2-GFP. Green (center), GFP (Kir6.2^+^); red (right), tdTomato (Ins1^+^); blue, DAPI. Scale bars, 20 µm. (D-E) Representative electrophysiology recordings of Ins1^Cre/0^; Kir6.2 KO pancreatic β cells before and after administering 10 µm glibenclamide. Membrane potential (D) and change in conductance before and after 10 µm glibenclamide administration (E) of control (left) and rescued (right) Ins1^Cre/0^; Kir6.2 KO pancreatic β cells indicate successful functional K_ATP_ channel restoration. ΔConductance (nS) (E): rescued (−0.4518 ± 0.05944 nS) vs control (−0.01033 ± 0.08042 nS): *P* = 0.0071, *t* = 4.415, df = 4.955, 95% confidence interval (CI) of the difference: 0.1837 to 0.6993; made by student’s two-tailed, unpaired *t*-test with Welch’s correction. (F) Amount increase in secreted insulin 20 minutes after a glucose stimulation before and after surgery. Rescued (0.2111 ± 0.05 719 mg/dL) vs control (0.04886 ± 0.03 646 mg/dL): *P* = 0.0272, *t* = 2.393, df = 18.94, 95% confidence interval (CI) of the difference: −0.3043 to −0.02030; made by student’s two-tailed, unpaired *t*-test with Welch’s correction. Control: *n* = 9 (A, B), 4 cells (E), 7 (F); Rescued: *n* = 10 (A, B), 3 cells (E), 14 (F). Statistical comparisons were made by two-way ANOVA with Šídák’s correction (A, B) and student’s two-tailed, unpaired *t*-test with Welch’s correction (E, F). Data are represented as mean ± SEM. *P < 0.05, **P < 0.01, ***P < 0.001, ****P < 0.0001, ns: not significant.

Our results thus far demonstrate that deleting K_ATP_ channels in pancreatic β cells significantly disrupts whole-body glucose homeostasis (Figures 1 and 3). To investigate whether restoring functional K_ATP_ channels specifically in pancreatic β cells could re-establish glucose metabolism, we re-expressed the K_ATP_ channels in Ins1^Cre/0^; Kir6.2 KO mice by delivering AAV9-FLEX-Kir6.2-GFP via the pancreatic duct. We found that, prior to surgery, Kir6.2 KO mice exhibited severely impaired glucose tolerance. However, only the mice injected with AAV9-Kir6.2 showed markedly improved glucose tolerance post-treatment (two-way ANOVA with Šídák’s correction) (Figure 5A and B and Supplementary Figure S8A-C), with significantly lower glucose levels after a 6-hour fast (Supplementary Figure S8D). The treated mice showed no signs of pancreatitis post-surgery, and fluorescent imaging confirmed successful delivery of the viral vectors to the pancreas (Figure 5C and Supplementary Figure S8E). We observed that glibenclamide, a K_ATP_ channel-specific blocker, induced action potentials by reducing background K^+^ conductance in the rescued pancreatic β cells, confirming the successful re-expression and function of K_ATP_ channels on the cell surface (Δ conductance: student’s two-tailed, unpaired *t*-test with Welch’s correction, *P* = 0.0071, *t* = 4.415, df = 4.955) (Figure 5D and E). While Kir6.2 KO mice initially failed to respond to acute glucose stimulation, reconstituting K_ATP_ channels in pancreatic β cells successfully restored GSIS (student’s two-tailed, unpaired *t*-test with Welch’s correction, *P* = 0.0272, *t* = 2.393, df = 18.94) (Figure 5F). These findings demonstrate that restoring K_ATP_ channels in the pancreatic β cells of Kir6.2 KO mice is sufficient to maintain adequate glucose homeostasis.

### K_ATP_ Channels Are Not Required for Neuronal Glucose Responsiveness

**Figure 6.**
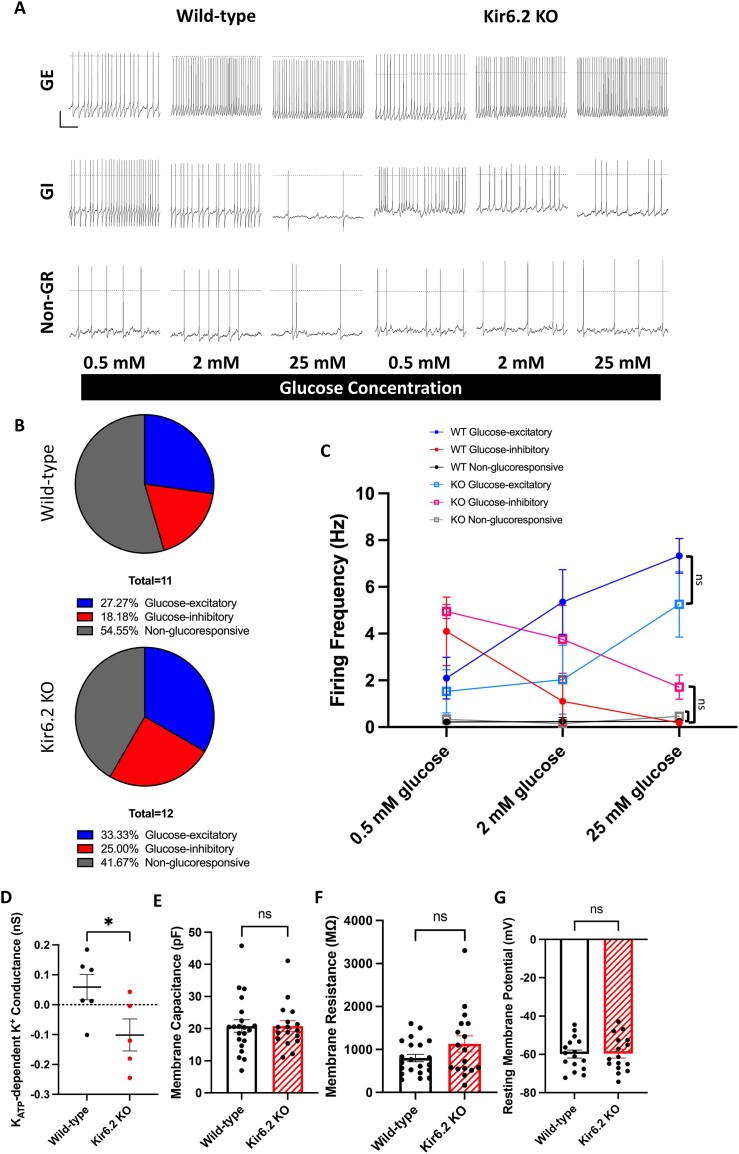
K_ATP_ channels are not required for neuronal glucose responsiveness. (A) Representative traces of WT and Kir6.2 KO GE, GI, and non-GR VMH neurons when exposed to different glucose concentrations. Scale bar *vertical*, 20 mV; scale bar *horizontal*, 1 sec. (B) Pie charts to demonstrate the proportion of GE, GI, and non-GR neurons among the spontaneous firing VMH neurons in WT (top) and Kir6.2 KO brain slices (bottom). (C) Firing frequency of WT and Kir6.2 KO GE, GI, and non-GR VMH neurons in different glucose concentrations. GE: *P* = 0.2733, F (1, 5) = 1.514; GI: *P* = 0.2311, F (1, 3) = 2.243; non-GR: *P* = 0.6857, F (1, 9) = 0.1747; made by two-way ANOVA with Šídák’s correction. (D) K_ATP_-dependent background K^+^ conductance in WT and Kir6.2 KO VMH neurons, calculated by subtracting steady-state 10 µm glibenclamide background K^+^ conductance from steady-state 300 µm diazoxide background K^+^ conductance. (E-G) Comparison of neuronal membrane properties, including membrane capacitance (E), membrane resistance (F), and resting membrane potential (G), in WT and Kir6.2 KO VMH neurons. WT: *n* = 6 (D), 21 (E-F), 16 cells (G); Kir6.2 KO: *n* = 5 (D), 17 (E-F), 16 cells (G). Statistical comparisons were made by Chi-square and Fisher’s exact test (B), two-way ANOVA with Šídák's correction (C), and student’s two-tailed, unpaired *t*-test with Welch’s correction (D-G). Data are represented as mean ± SEM. *P < 0.05, ns: not significant.

The K_ATP_ channel is widely recognized as the primary glucose-sensing molecular component in neurons—particularly in ventromedial hypothalamic (VMH) neurons, which fine-tune glucose homeostasis by detecting changes in circulating glucose levels.^35,49^ However, electrophysiological recordings from Kir6.2 KO mouse brain slices revealed that the absence of functional K_ATP_ channels did not abolish glucose responsiveness in the VMH neurons (Figure 6A). Among the recorded VMH neurons, 68.75% (11/16) of wild-type (WT) neurons and 75% (12/16) of Kir6.2 KO neurons exhibited spontaneous firing, with no significant difference between the groups (Chi-square analysis, *P* = 0.6942; Fisher’s exact test, *P* > 0.9999). In both groups, glucose responsiveness was distributed similarly: 27.27% (3/11), 18.18% (2/11), and 54.55% (6/11) of WT neurons, and 33.33% (4/12), 25% (3/12), and 41.67% (5/12) of Kir6.2 KO neurons were classified as glucose-excitatory (GE), glucose-inhibitory (GI), and non-glucoresponsive (non-GR), respectively (Figure 6B), with no significant differences (Chi-square analysis, *P* = 0.8224; Fisher’s exact test, *P* > 0.9999). The firing frequencies of VMH neurons were also comparable between WT and Kir6.2 KO groups under different glucose concentrations (two-way ANOVA with Šídák’s correction, GE: *P* = 0.2733, F (1, 5) = 1.514; GI: *P* = 0.2311, F (1, 3) = 2.243; non-GR: *P* = 0.6857, F (1, 9) = 0.1747) (Figure 6C and Supplementary Figure S8). Diazoxide significantly increased background K^+^ conductance in WT but not in Kir6.2 KO neurons (Figure 6D), confirming that Kir6.2 is a vital molecular component of the neuronal K_ATP_ channels. Further analyses of membrane capacitance, membrane resistance, and resting membrane potential revealed no differences between WT and Kir6.2 KO VMH neurons (Figure 6E-G). These findings suggest that K_ATP_ channels may not be required for glucose sensing in VMH neurons.

## Discussion

In this study, we demonstrated that K_ATP_ channels are crucial for glucose homeostasis, primarily by modulating GSIS through the regulation of pancreatic β-cell excitability. Mice lacking functional K_ATP_ channels exhibited impaired glucose homeostasis (Figure 1), including reduced glucose tolerance, exaggerated glucose release in response to 2DG-induced glycopenia, and impaired GSIS, with no observable abnormalities in insulin sensitivity or body weight. Similar deficits were explicitly observed in mice lacking functional K_ATP_ channels in pancreatic β cells, but none of the neuronal populations examined showed these effects (Figures 2-3, Supplementary Figure S6A and B), all of which have previously been associated with K_ATP_-channel-mediated glucose homeostasis regulation.^13,19,35,50,51^

The regulation of whole-body glucose homeostasis involves multiple mechanisms, including insulin secretion, insulin sensitivity, and glucose effectiveness.^52–54^ A commonly accepted explanation for the severe hypoglycemia observed in Kir6.2 KO mice following insulin administration is that, without functional neuronal K_ATP_ channels, mice have enhanced insulin sensitivity^35,41^ and fail to initiate appropriate counter-regulatory responses to glycopenia, including hypoglycemia-induced glucagon secretion.^35,55^ In early metabolic studies, the standard procedure was to perform overnight (over 16 hours) fasting in mice prior to the IPGTT or ITT assays.^19,35,41,42,56,57^ However, prolonged fasting durations prior to metabolic assays have become unadvised because it is known to induce metabolic stress and enhance insulin sensitivity,^58,59^ and a fasting duration of 6 hours followed by a 2 g/kg glucose dose of injection is advised, as it is the most optimal condition to assess glucose tolerance.^58,60^ As the 3-stage blood glucose monitoring results indicated, the blood glucose levels were comparable among groups after 12 hours of fasting (two-way ANOVA with Šidák’s correction, WT vs Kir6.2 KO: *P* = 0.9377; Ins1^Cre/0^; Kir6.2^flox/+ vs flox/flox^: *P* = 0.9992; Nestin^Cre/0^; Kir6.2^flox/+ vs flox/flox^: *P* = 0.4032). This suggests that prolonged fasting may serve as a potential factor in why hyperglycemia and severe glucose intolerance were not observed in previous studies. Further prolongation of the fasting duration to 24 hours triggered hypoglycemia in both whole-body and pancreatic β cell-specific but not neuronal-specific Kir6.2 KO mice (Figure 4A and Supplementary Figure S7), which may have been previously misinterpreted as a consequence of enhanced insulin sensitivity in Kir6.2 KO mice. Initially believed to result from dysfunctional pancreatic β cell-specific K_ATP_ channels that are typically activated in response to low glucose levels to hyperpolarize pancreatic β cells and prevent basal insulin secretion,^61^ our results were not able to support this hypothesis, as the Kir6.2 KO mice did not exhibit hyperinsulinemia after prolonged fasting (Figure 4D and Supplementary Table S3). Although Kir6.2 KO pancreatic β cell resting membrane potential seemed more depolarized in the traces (Figure 5D left), the amount of data obtained was not yet sufficient to draw this conclusion. Glucose-stimulus-to-insulin-secretion coupling becomes less efficient in Kir6.2 KO mice,^62^ which could be the reason for not being able to observe any hyperinsulinemia phenotypes. Detailed investigation regarding changes in ion channel composition and density should be conducted to validate the presence of a causal link to dampened insulin secretion events. Cell signaling pathways can alter ion channel expression, further altering pancreatic β cell excitability. For example, leptin increases K_ATP_ channel conductance by increasing the surface density of this channel, resulting in inhibition of insulin secretion.^63^ Also, high voltage-gated calcium channel (HVCC) membrane surface expression mediates insulin secretion and increases glucose-stimulated intracellular calcium oscillation frequency upon deletion in pancreatic β cells.^64^ Apart from great transient calcium influx via HVCC, store-operated calcium entry mediated by TRPC1-containing protein complex occurs under endoplasmic reticulum calcium depletion. If this pathway is blocked, GSIS becomes impaired.^65^ Whether and how the dynamics of ion channel composition and expression density on the pancreatic β cell plasma membrane upon removal and restoration of functional K_ATP_ channels require further validation. Nonetheless, the occurrence of this phenotype exclusively in mice lacking pancreatic K_ATP_ channels strongly points to a pancreatic origin for this trait (Figure 4A).

Furthermore, 2DG-induced glycopenia (Figure 3B and Supplementary Figure S6B) did not support the hypothesis that defects in counter-regulatory mechanisms are mediated by the lack of functional K_ATP_ channels in catecholaminergic neurons, which would impair adequate glucagon secretion due to defective glucose-sensing ability.^47,66^ Instead, our results showed a significant increase in blood glucose levels only in whole-body and pancreatic β cell-specific Kir6.2 KO mice following 2DG administration (Figure 3A-C). In these cases, counter-regulatory mechanisms should be promptly activated across all groups, indicating that the absence of functional neuronal K_ATP_ channels does not impair glycopenia sensing or glucagon secretion (Figure 3D). Following 2DG injection, blood glucose levels remained significantly higher only in whole-body and pancreatic β cell-specific Kir6.2 KO mice (Figure 3A-C). 2DG blocks glycolysis, leading to low ATP production and consequently, suppressed insulin secretion in pancreatic β cells. Concurrently, glucose uptake in peripheral tissues is inhibited, and counter-regulatory mechanisms, including increased glycogenolysis and gluconeogenesis, are triggered.^67^ As blood glucose levels rise, glucose outcompetes 2DG, allowing glycolytic metabolism to resume in pancreatic β cells (Figure 3E). Consequently, GSIS is restored as K_ATP_ channels can be closed due to increased ATP production from the resumed glycolysis. The elevated insulin levels are then sufficient to lower blood glucose by enhancing glucose uptake and suppressing gluconeogenesis. These findings underscore the critical role of pancreatic glucose sensing in the counter-regulatory response to glycopenia.

Previous studies have aimed to unveil the importance of neuronal K_ATP_ channels in glucose homeostasis regulation. As mentioned previously, K_ATP_ channels are expressed in many glucose-sensing neuronal subpopulations, including ARC AgRP and POMC neurons and VMH SF1 neurons,^19,35,40,56,68^ and have been treated as the major component for insulin- and leptin-induced glucose homeostasis regulation.^14,15,19,68^ Without proper-functioning K_ATP_ channels, these neurons lose their glucose-sensing properties with simultaneously impaired systemic glucose homeostasis.^19,35,56,69^ However, we did not observe glucose dyshomeostasis in mice lacking functional neuronal K_ATP_ channels (Figures 1H and I, 2, 3B, and Supplementary Figure S6A and B). Using the Kir6.2^flox^ mouse strain allowed us to precisely target specific neuronal populations, and all mice carried Ai14 alleles to rigorously exclude ectopic Cre activity. This was especially important in Nestin^Cre/0^, POMC^Cre/0^, and TH^Cre/0^ mice, which can otherwise result in unintended whole-body Kir6.2 KO and potentially lead to erroneous conclusions due to undetected ectopic Cre expression.^34,70,71^ Additionally, some Cre-expressing mice themselves exhibit metabolic abnormalities^72–74^: Nestin^Cre/0^ mice having lower body weight and lower blood glucose levels and AgRP^Cre/+^ mice being glucose intolerant.^70^ Comparing mice without proper littermate controls expressing the same Cre recombinase can lead to inaccurate or misleading results. No glucose homeostasis impairment in mice lacking functional neuronal K_ATP_ channels was detected after excluding mice with ectopic Cre expression. In summary, our results show that neuronal K_ATP_ channels are not required for central regulation of glucose homeostasis.

Glucose sensing is a well-established phenomenon in neurons, particularly those involved in energy metabolism.^75–79^ Genetic ablation or pharmacological inhibition of K_ATP_ channels has been shown to disrupt the glucose-sensing abilities of these neurons accompanied by impaired glucose homeostasis.^19,35,80^ As a result, K_ATP_ channel-mediated glucose sensing in neurons has been recognized as a key physiological mechanism for brain-mediated control of glucose homeostasis. However, in our study, we were surprised to find that the glucose-sensing properties in the CNS were not disrupted in VMH neurons of whole-body Kir6.2 KO mice (Figure 6). This observation suggests that neuronal K_ATP_ channels are neither essential for glucose homeostasis nor necessary for glucose responsiveness. This unexpected finding raises two important questions: (1) the specific roles that neuronal K_ATP_ channels fulfill and (2) other molecular mechanisms that may act as crucial glucose-sensing components. As a highly metabolically active organ,^81,82^ the brain requires neuronal K_ATP_ channels, activated by low intracellular ATP levels, to induce hyperpolarization and prevent excitotoxicity under hypoxic conditions.^83,84^ Therefore, central K_ATP_ channels may serve neuroprotective roles during hypoxia and ischemia. Additionally, recent studies have identified a variety of ion channels with possible glucose-sensing properties. These channels may act as alternative regulators of energy metabolism, providing new insights into glucose-sensing mechanisms that extend beyond K_ATP_ channels.^85^

Regarding technical limitations, our conclusions rely on a commonly used glucose homeostasis assessment—IPGTT. While this method is straightforward and widely adopted, it is influenced by multiple factors, including insulin secretion, insulin sensitivity, glucose utilization, and glucose production,^86,87^ and provides limited mechanistic insights. Previous studies have demonstrated the neuronal influence on glucose homeostasis and the role of K_ATP_ channels in glucose metabolism using more sensitive techniques, such as hyperinsulinemic-euglycemic clamps, which are technically challenging and require sophisticated surgery but provide a more precise assessment of insulin action.^45,69,88,89^ However, it is worth noting that IPGTT has also been widely used in prior studies to assess the effects of neuronal K_ATP_ channels on glucose homeostasis, concluding their involvement in whole-body glucose homeostasis.^19,45^ Building on this approach and following established IPGTT guidelines,^59,90^ we are confident that IPGTT can be adapted as a primary screening method to evaluate the impact of K_ATP_ channel deletions across multiple mouse lines. This method enables us to screen multiple lines efficiently under standardized conditions. Another limitation of this study is the lack of direct K_ATP_ channel manipulation in the neurons apart from genetic ablation upon development. For example, AgRP neurons are essential for feeding but only in adult mice and can be compensated when ablated in neonates.^91^ As the conditional KO mice lose functional K_ATP_ channels at birth, it remains uncertain whether this may lead to a series of compensatory mechanisms via other neurons in the metabolism-regulating neural circuitry. However, the current methods available for adult-stage channel manipulation are susceptible to confounding factors. Intracerebroventricular injections of channel openers or blockers are a common method to directly target the ion channel of interest located only in the brain, yet hydrophilic drugs administered are readily detectable in the systemic circulation within the first hour post-administration.^92^ Viral delivery of channel mutants and optogenetic- or chemogenetic-(in)activated channels is also a commonly practiced method despite being prone to alter metabolic manifestations due to hypothalamic lesions.^93^ Inducible Cre lines could also be implemented to ablate or restore channel expression at a designated time point, but the recombination efficiency is rarely 100%. Still, no alteration in glucose homeostasis was observed in any of the mice with individual neuronal subpopulation-targeted K_ATP_ channel ablation, and VMH neurons were still glucose responsive without neuronal K_ATP_ channels, suggesting the unlikelihood of compensation. Most importantly, recent revisits of AgRP neuron ablation in adult mice unveiled the dispensability of these neurons in *ad libitum* feeding and body weight maintenance, challenging previous experimental paradigms with new models and approaches.^94^ Hence, we believe that using this new Kir6.2^flox^ mouse provides a more accurate physiological phenotype.

In conclusion, our results demonstrate that pancreatic K_ATP_ channels indeed serve as the systemic glucostat, and glucose homeostasis becomes dysregulated in mice without these channels in the pancreas. Furthermore, functional neuronal K_ATP_ channels are not required for glucose-sensing properties in the neurons; on that account, the lack of functional neuronal K_ATP_ channels does not result in glucose dyshomeostasis in mice.

## Supplementary Material

zqaf002_Supplemental_File

## Data Availability

The data supporting this study’s findings are available from the corresponding author, S.-B. Yang, upon reasonable request.
