# Supplementary material for: Systemic Glucose Homeostasis Requires Pancreatic but Not Neuronal ATP-sensitive Potassium Channels
Source: Function (Oxf). 2025 Jan 14;6(1):zqaf002. doi: 10.1093/function/zqaf002 (PMC11815579; doi:10.1093/function/zqaf002)
Supplement: zqaf002_Supplemental_File [file zqaf002_supplemental_file.docx]

**A**


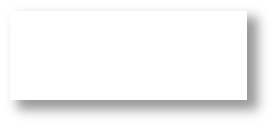

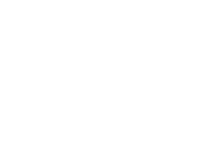

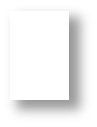

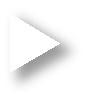

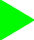

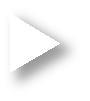

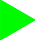

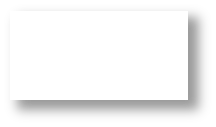

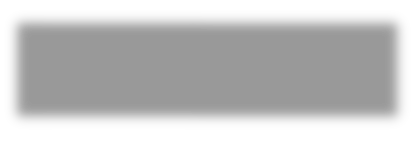

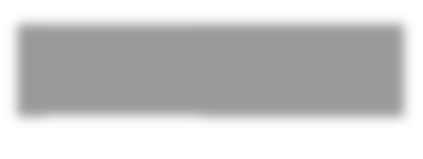

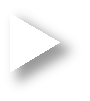

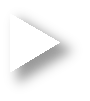

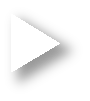

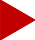

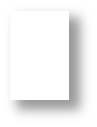

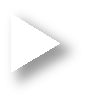

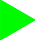

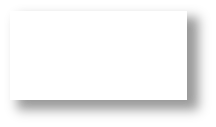

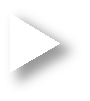

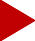

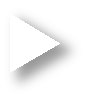

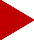


Exon

Frt

loxP

Frt loxP

loxP

polyA neo polyA

Exon’ F2A

human beta actin promoter

Exon”

LacZ

intron

**Kir6.2 Wild-type: Kir6.2-FloxA:**

**Kir6.2-FloxC:**

Exon’ Frt loxP

intron

Exon”

loxP

## B AAV9-FLEX-Kir6.2-GFP vector design:

**C**

## Schematic diagram of paradigm for assessing

CAG

**VMH neuron glucose responsiveness:**

2A

**0.5 mM 2 mM 25 mM**

**aCSF**

**Glucose**

**Glucose Glucose**

## Schematic diagram of mouse breeding strategy for intraductal viral administration:


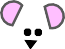

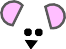

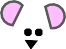


**0 min**

**3 min**

**6 min 9 min 12 min**

## Schematic diagram of paradigm for assessing

×
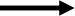
 **K_ATP_-dependent K+ conductance:**

**aCSF 0.5 mM Glucose**

**0.5 mM Glucose**

*Ins1-RFP-Cre+/0; Kir6.2-FcFcN/+; ai14+/-*

*Kir6.2-FcFcN/+; ai14+/-*

*Ins1-RFP-Cre+/0; Kir6.2-FcFcN/FcN; ai14+/-*

**0 min**

**300 μM Diazoxide 10 μM Glibenclamide**

**2 min 7 min 12 min**

## Schematic diagram of timeline for intraductal viral administration:

**recovery**

**IPGTT re-test**

**surgery 2 weeks IPGTT pre-test post-surgery**

**sacrifice for validation**

**(age: 6 weeks)**

**Supplementary Figure 1: Schematic diagrams of specified experimental procedures used in this study. A:** Schematic diagram of Kir6.2 mouse strain genetic design. Kir6.2 is encoded by the *Kcnj11* gene that contains an exon of 3,115 base pairs (*top*). In the Kir6.2-FloxA allele (*middle*), the bacterial *lacZ* reporter gene and the neomycin selection marker gene, together flanked by two flippase (Flp) recognition target (Frt) sequences, are inserted between the *Kcnj11* exon. A less efficient self-cleaving peptide sequence F2A is added to the 5’ end of the *lacZ* gene, the neomycin expression is driven by the human beta-actin promoter, and poly-A tails are added to the 3’ end of both genes. The insertion of these genes creates a disruption of the *Kcnj11* gene. Three loxP sequences are inserted (listed in the order from 5’ to 3’): (1) between the lacZ and neomycin genes, (2) after the 3’ Frt sequence, and (3) after the 3’ exon fragment, with the latter two loxP sequences flanking the 3’ exon fragment. The Kir6.2-FloxC mouse strain (*bottom*) is derived from mice that carried both Kir6.2-FloxA and Actin-Flp alleles. Actin-driven Flp cleaves the Frt sites in the Kir6.2-FloxA and removes the *lacZ* and neomycin, leading to global reconstitution of the *Kcnj11* exon, with the 3’ fragment flanked by two loxP sequences. The Kir6.2-FloxC mouse strain will express functional Kir6.2 and is used as a conditional Kir6.2 KO mouse strain. When crossed with a Cre-carrying mouse strain, the 3’ exon fragment is excised in the Cre-expressing cells. **B:** Schematic diagrams of the AAV9-FLEX-Kir6.2-GFP vector design (*top*), the mouse breeding strategy to obtain whole-body Kir6.2 KO mouse strain carrying pancreatic-β-cell-specific Cre (Ins1-RFP-Cre) and reporter (Ai14) alleles (*middle*), and the timeline for intraductal viral administration experiments (*bottom*). **C:** Schematic diagrams of brain slice recording paradigms for assessing VMH neuron glucose responsiveness (*top*) and K_ATP_-dependent K+ conductance (*bottom*).

**Alt text:** Graphic visualization of specific experimental procedures used in this study, including the genetic design of the conditional Kir6.2 knockout mouse strain in **A**, the viral vector design, breeding strategy, and schematic timeline for intraductal viral administration rescue experiments in **B**, and the schematic diagram of paradigms used for ventromedial hypothalamic neuron electrophysiology recordings in **C**.

**A B**


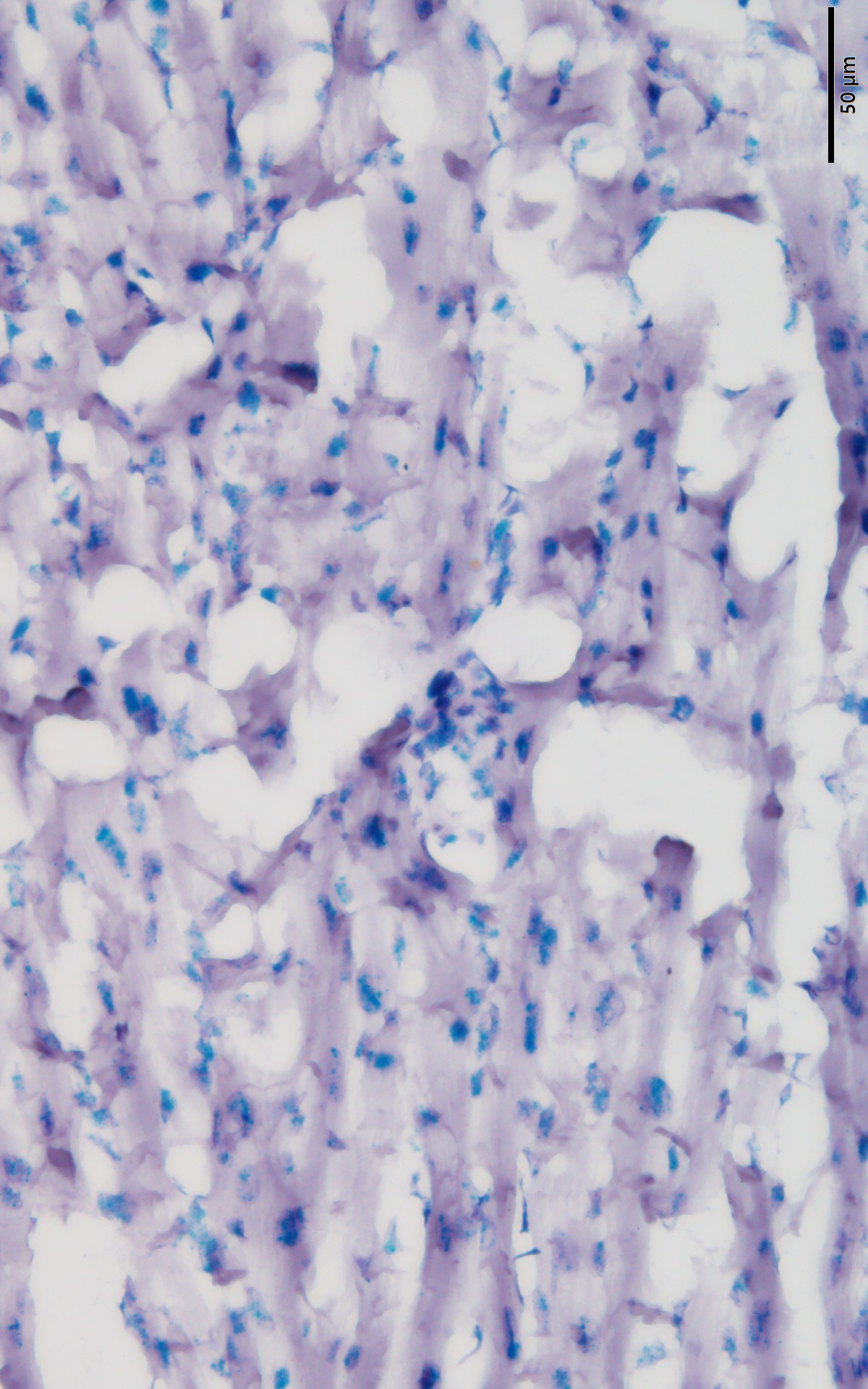

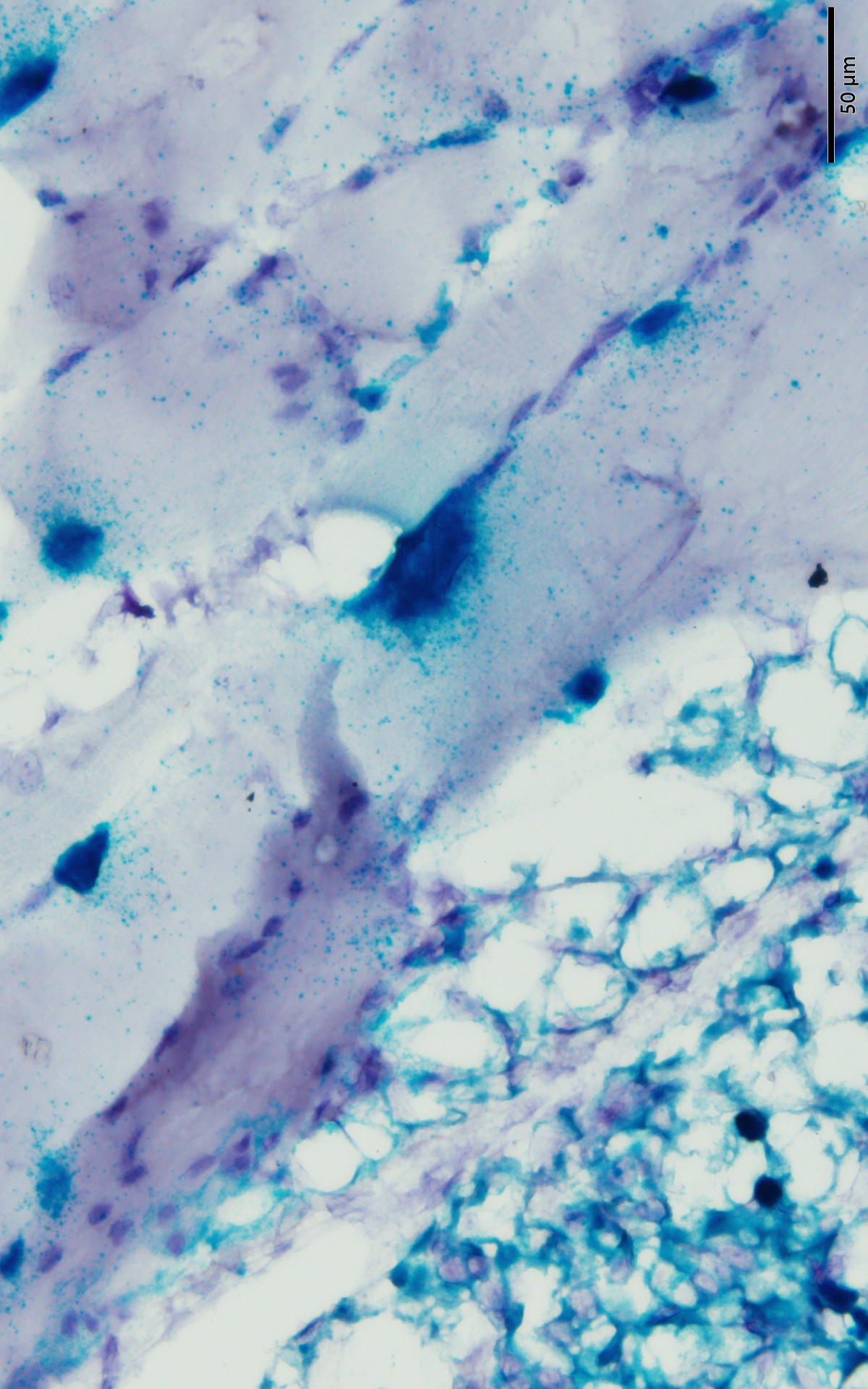


**C**


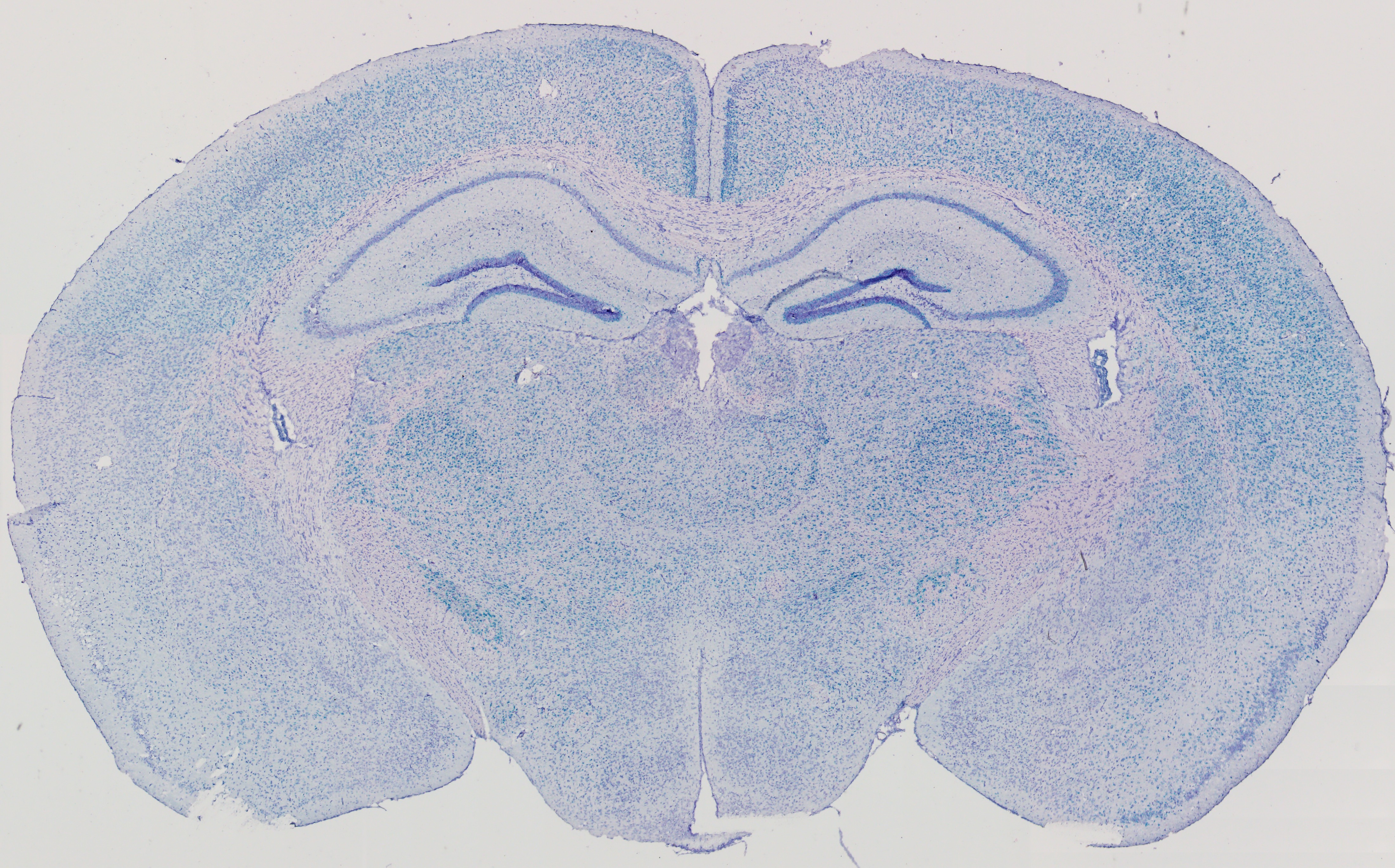

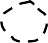

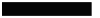


VP

STh

LH

DMH

VMH

ARC

**D E F G**


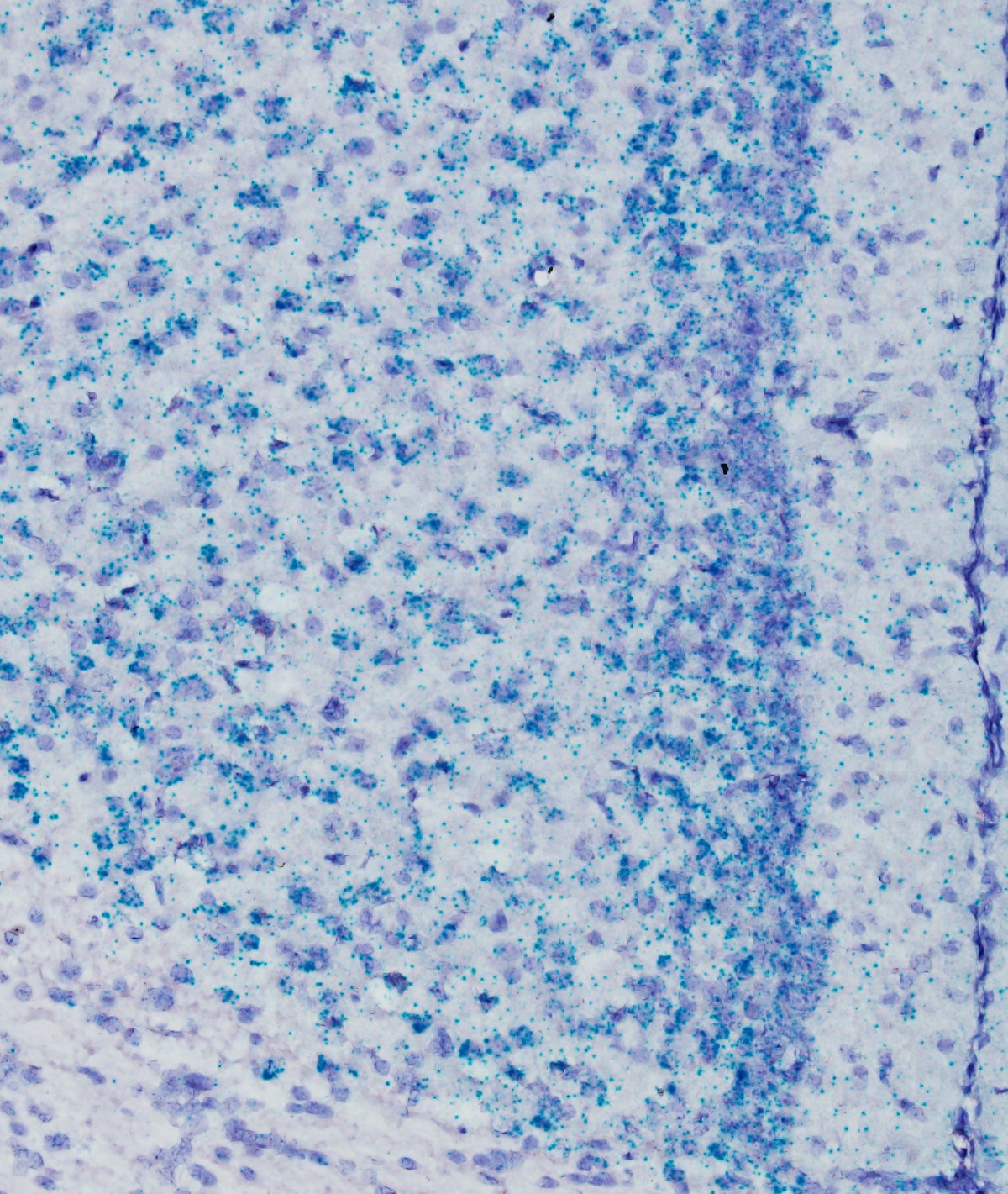

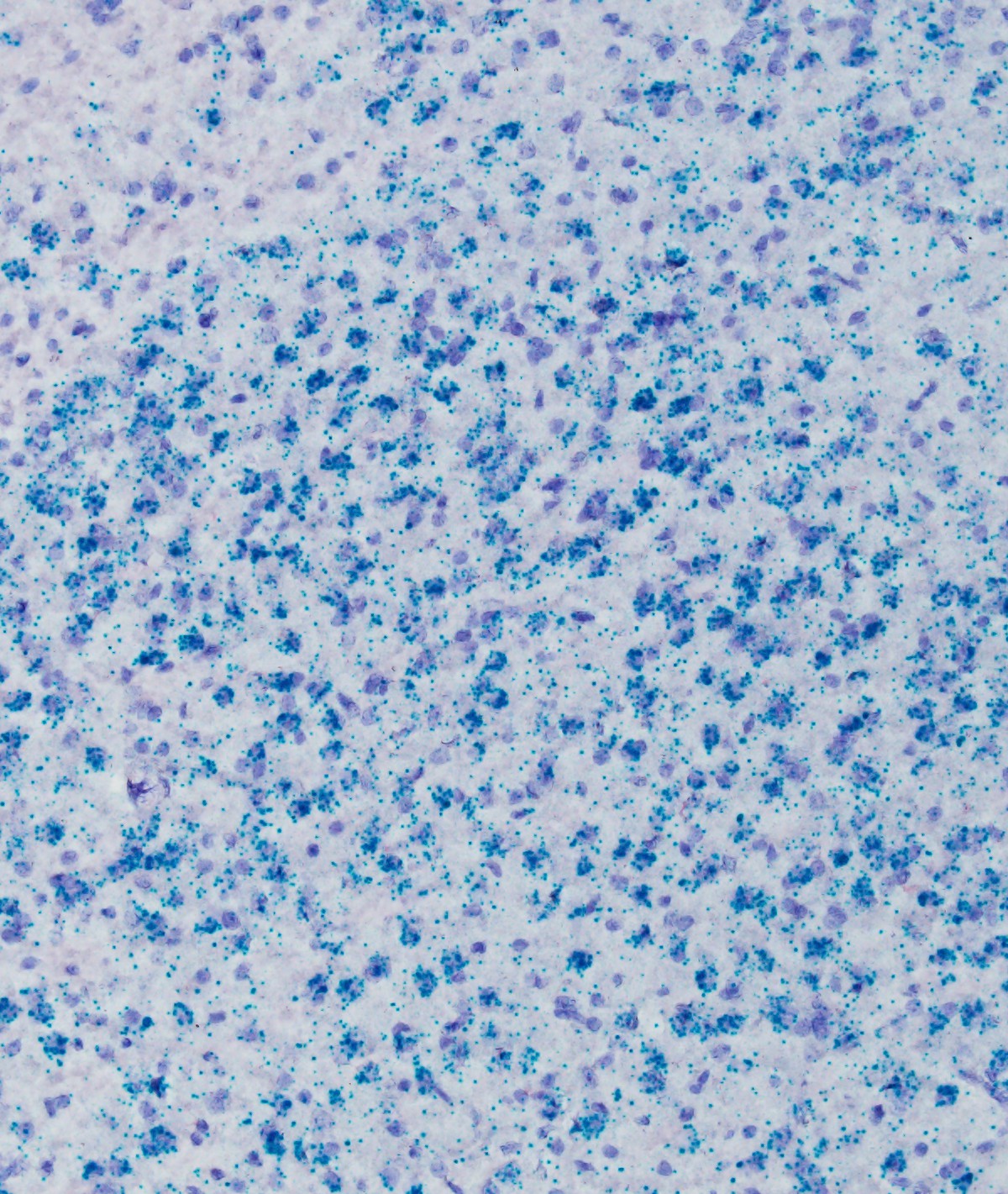

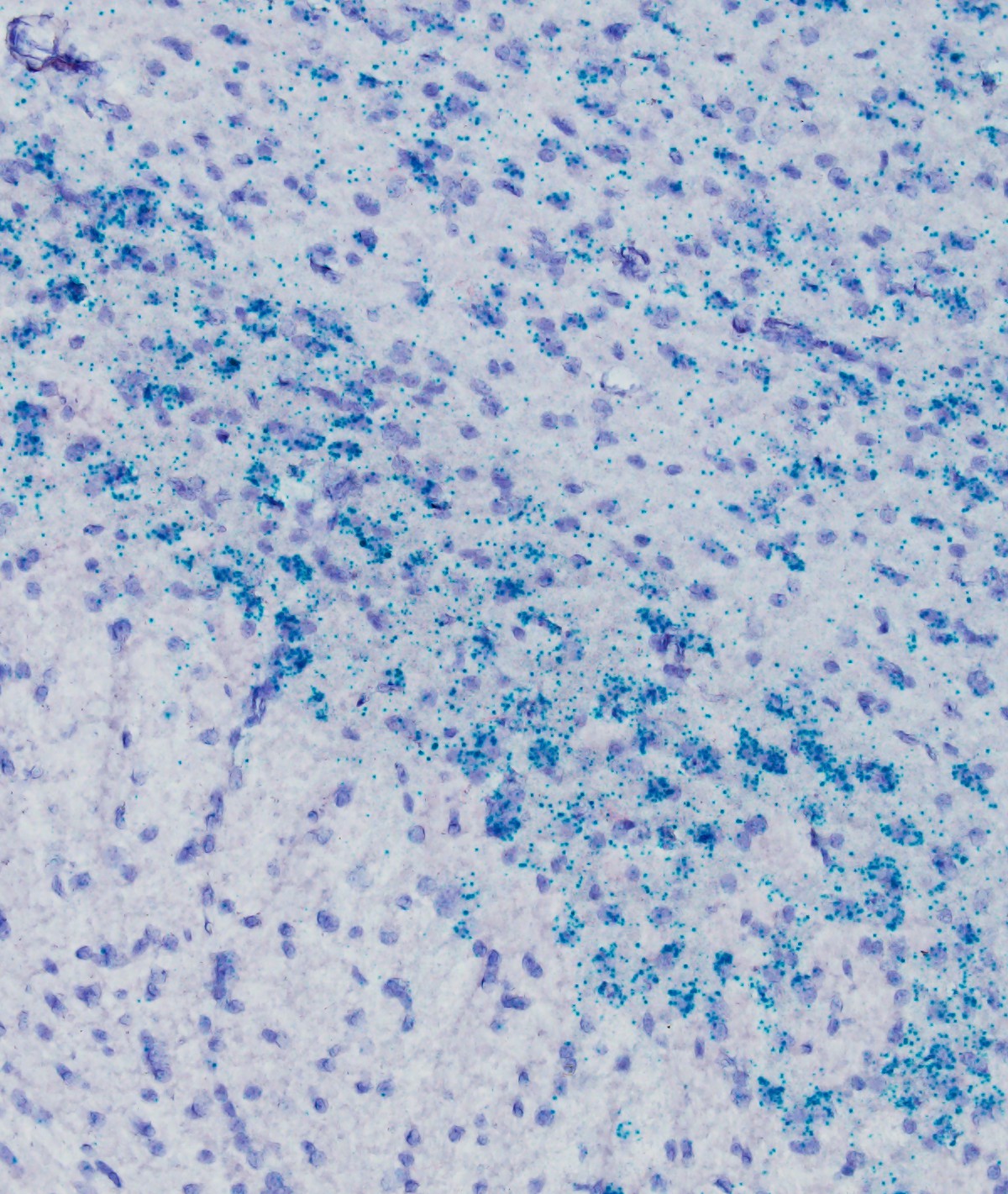

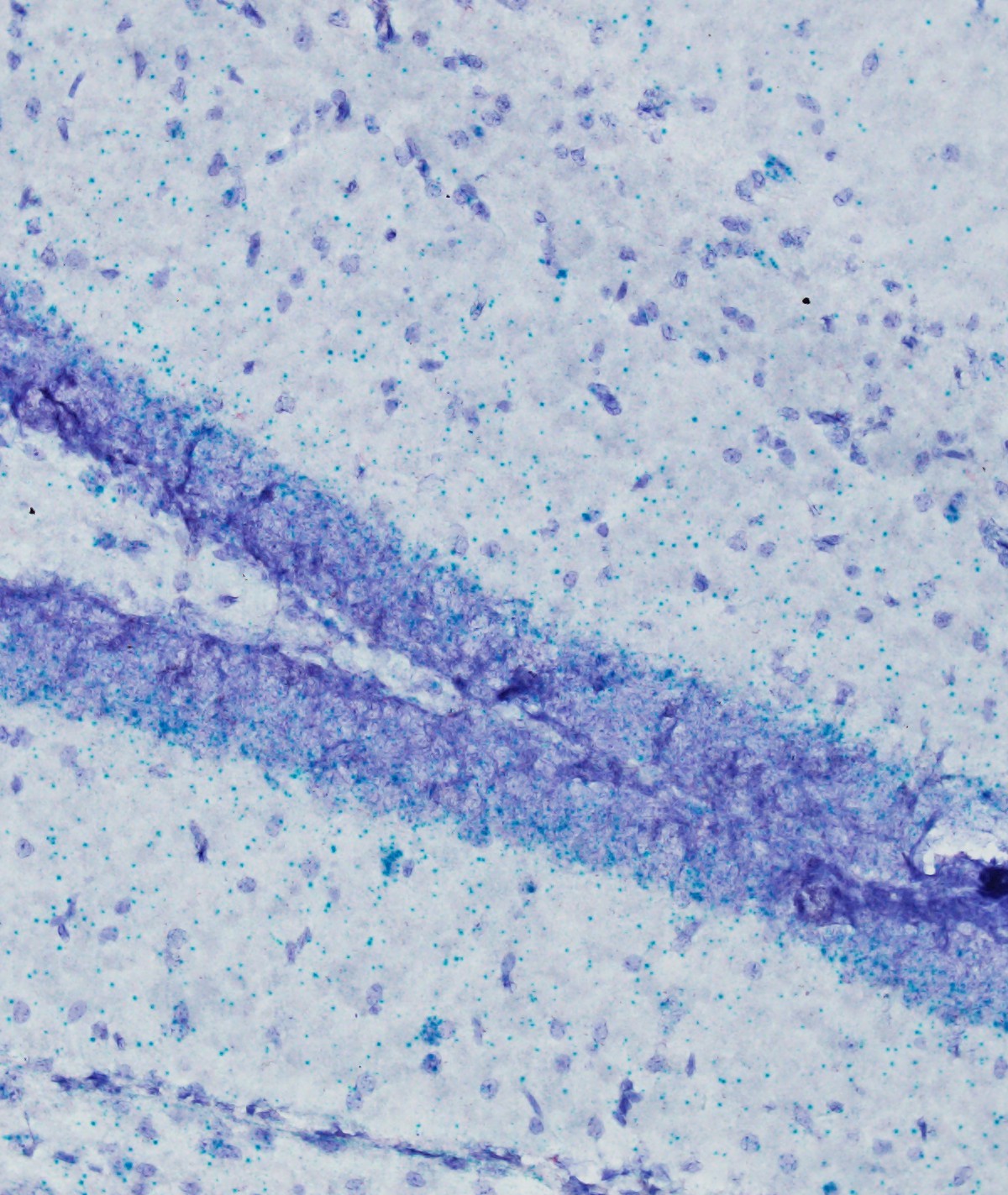

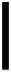

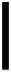

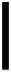

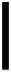


**H I J K**


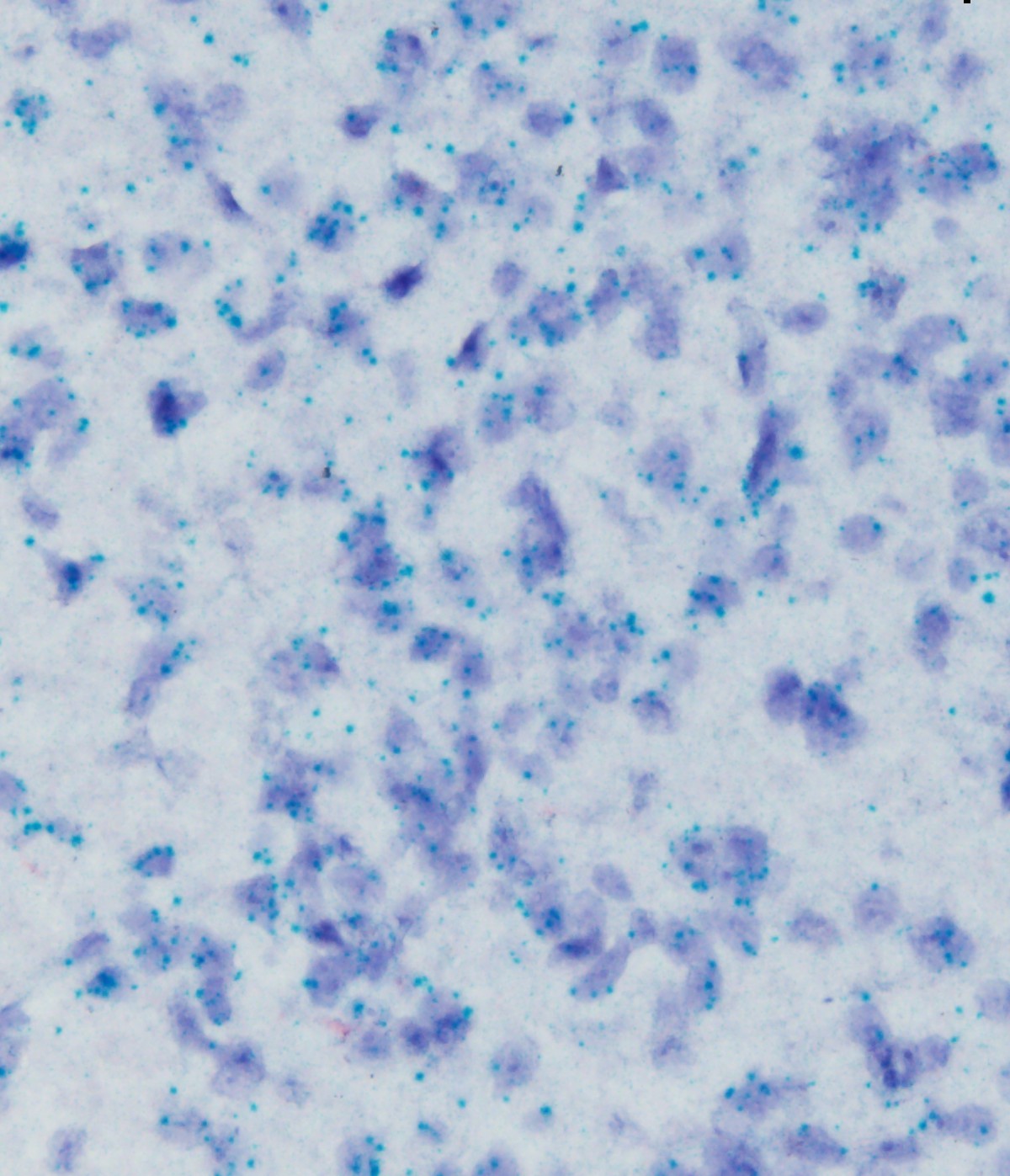

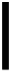

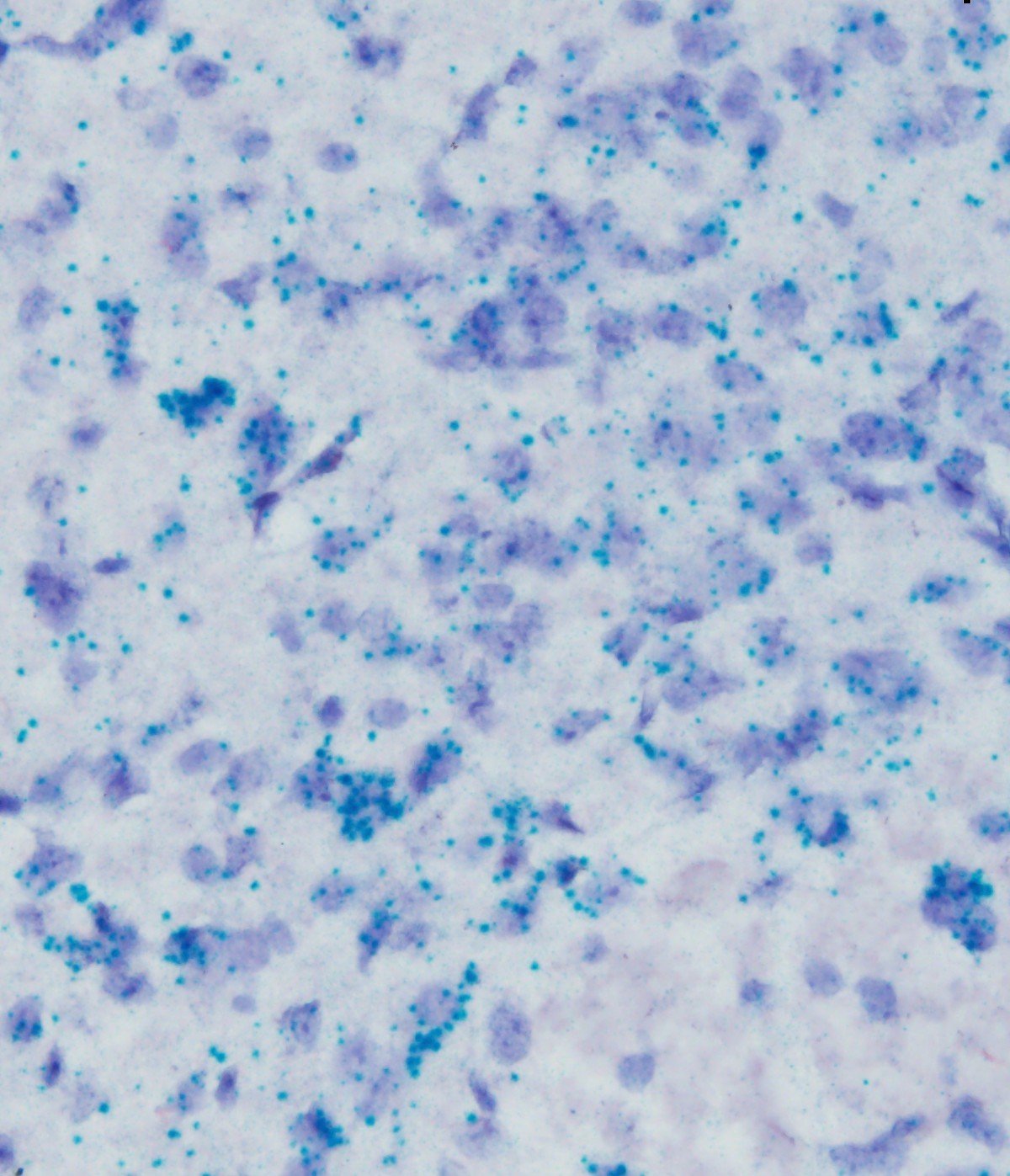

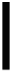

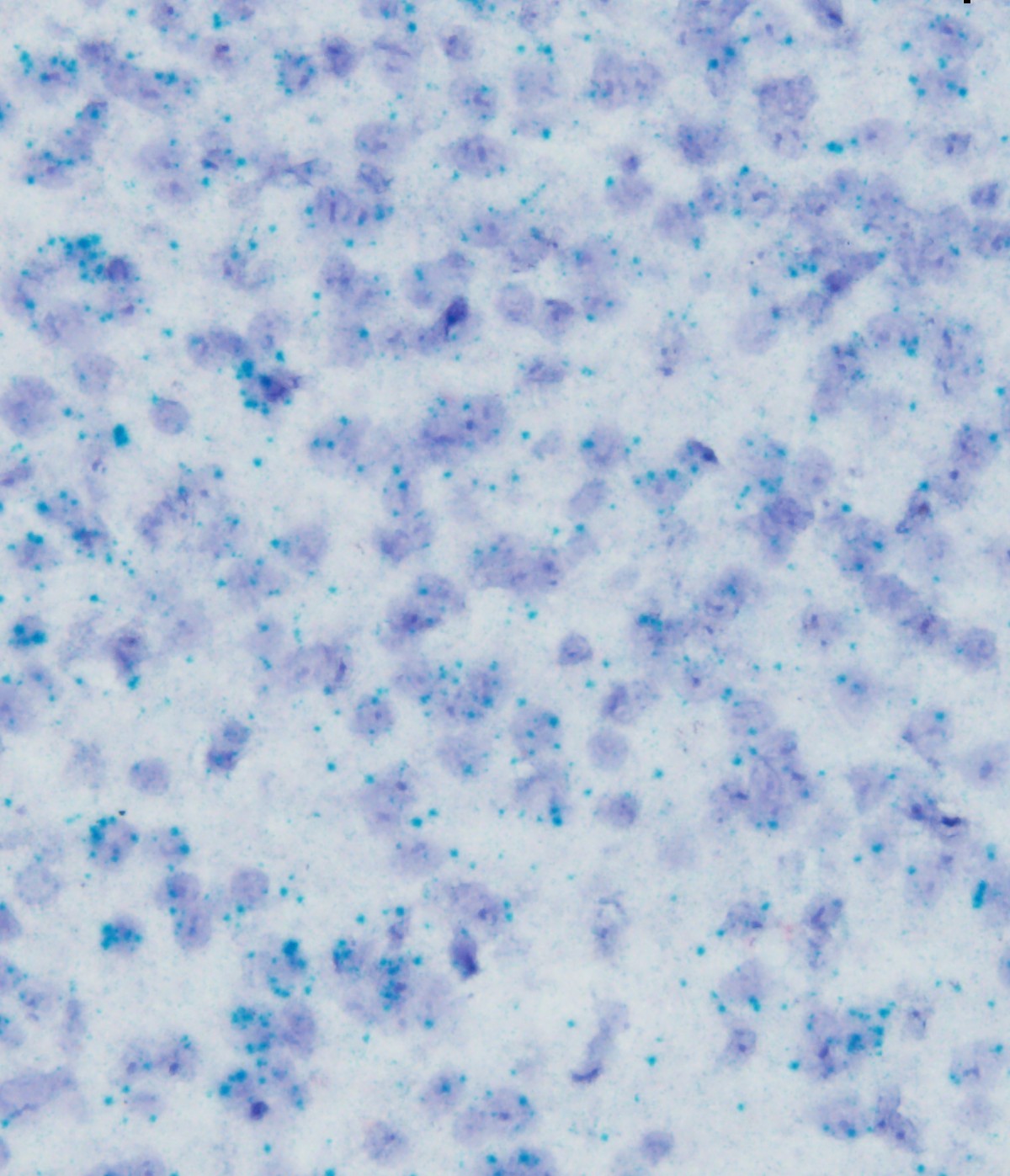

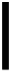

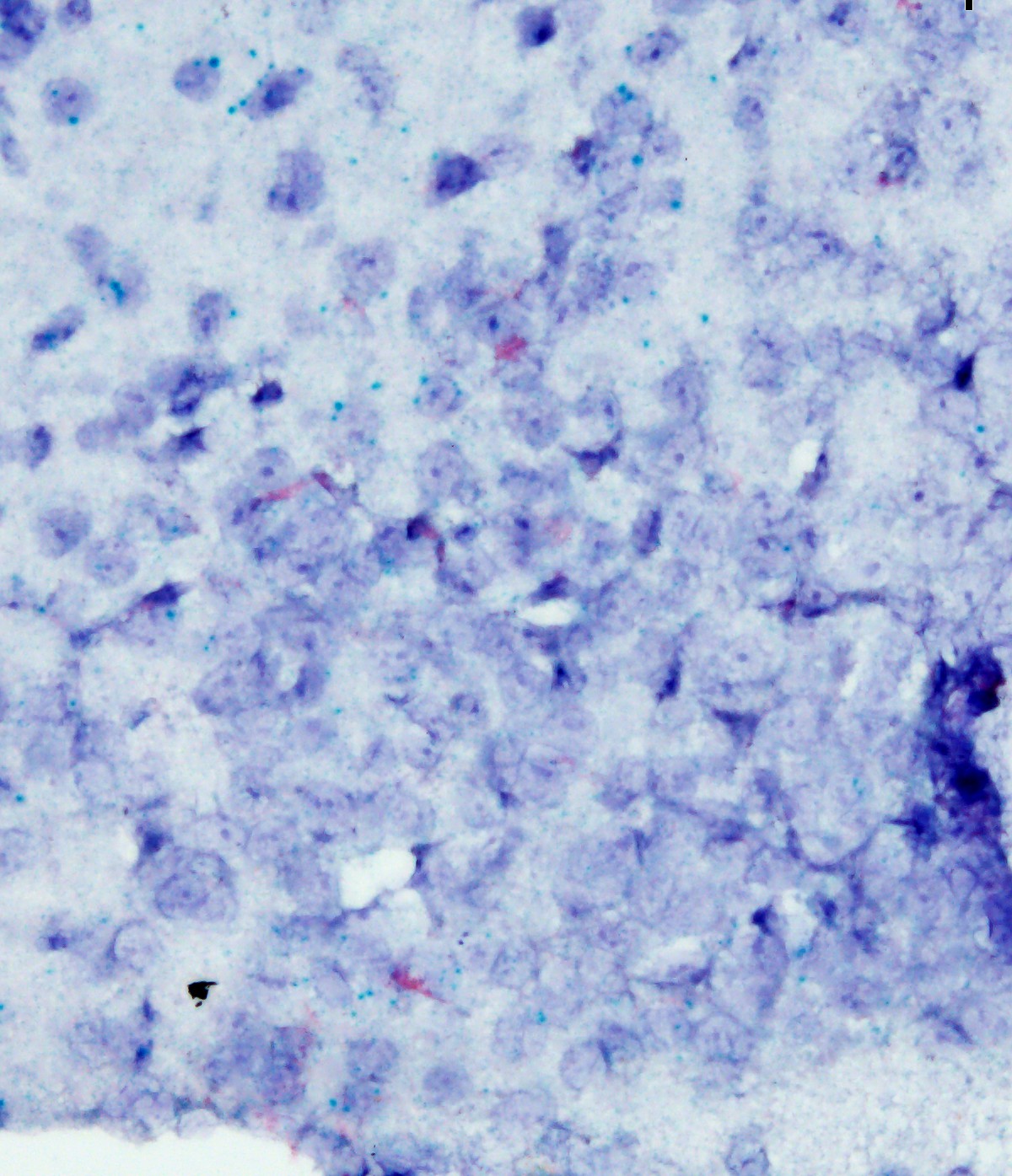

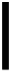


|  |  |  |  |
| --- | --- | --- | --- |

## Supplementary Figure 2: Validation of *Kcnj11* mRNA expression pattern.

(legend on next page)

## Supplementary Figure 2: Validation of *Kcnj11* mRNA expression pattern.

RNAscope staining of *Kcnj11* mRNA (*blue*) in WT mouse heart (**A**), skeletal muscle (**B**), and brain (**C**–**K**), counterstained with hematoxylin (*purple*). In the brain, signals were detected in cortex (**D**), hippocampal granule cells (**E**), subthalamic nucleus (STh) (**F**), and ventral posterior thalamic nucleus (VP) (**G**), lateral hypothalamus (LH) (**H**), dorsomedial hypothalamus (DMH) (**I**), ventromedial hypothalamus (VMH) (**J**), and arcuate nucleus (ARC) (**K**). Scale bars, 500 μm (**C**); 100 μm (**D**–**G**); 50 μm (**A**–**B**, **H**–**K**).

**Alt text:** *Kcnj11* mRNA expression labeled in blue is present in mouse cardiac and skeletal myocytes shown in **A** and **B** and neurons located throughout the brain shown in **C**, including but not limited to the cortex, hippocampal granule cells, subthalamic nucleus, ventral posterior thalamic nucleus, lateral hypothalamus, dorsomedial hypothalamus, ventromedial hypothalamus, and arcuate nucleus shown in **D** to **K** with purple counterstain to label the cell nuclei.

**A**


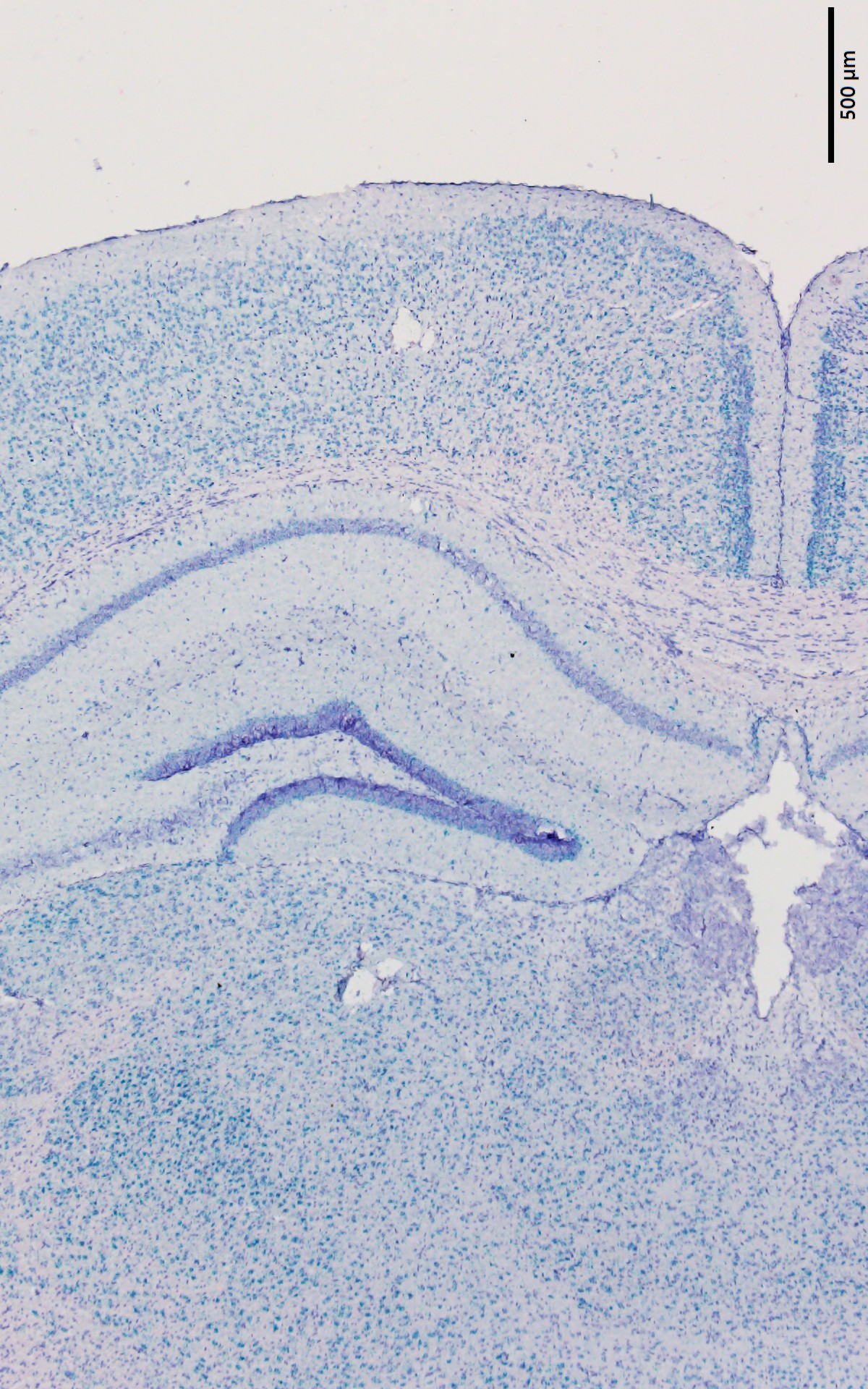

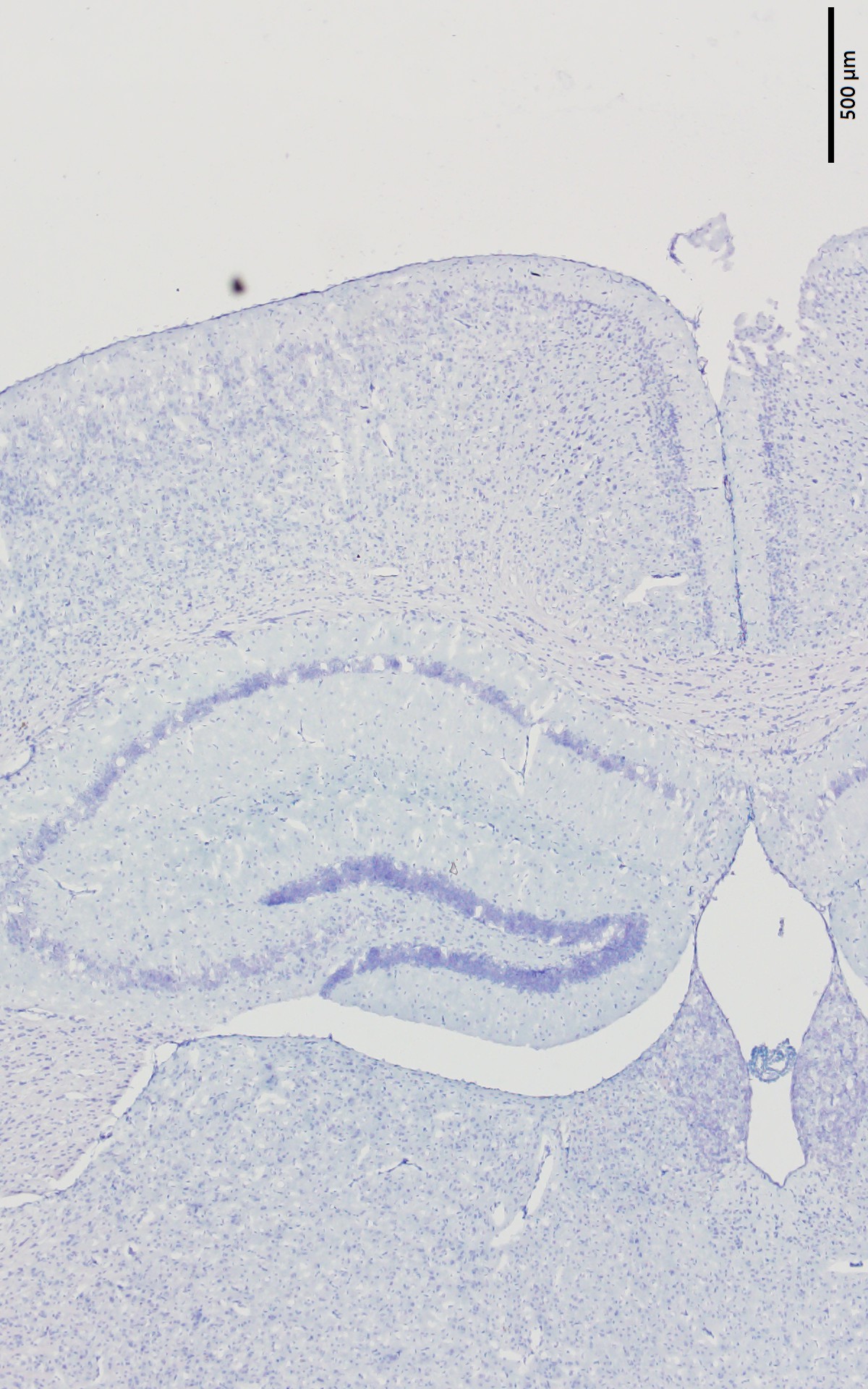

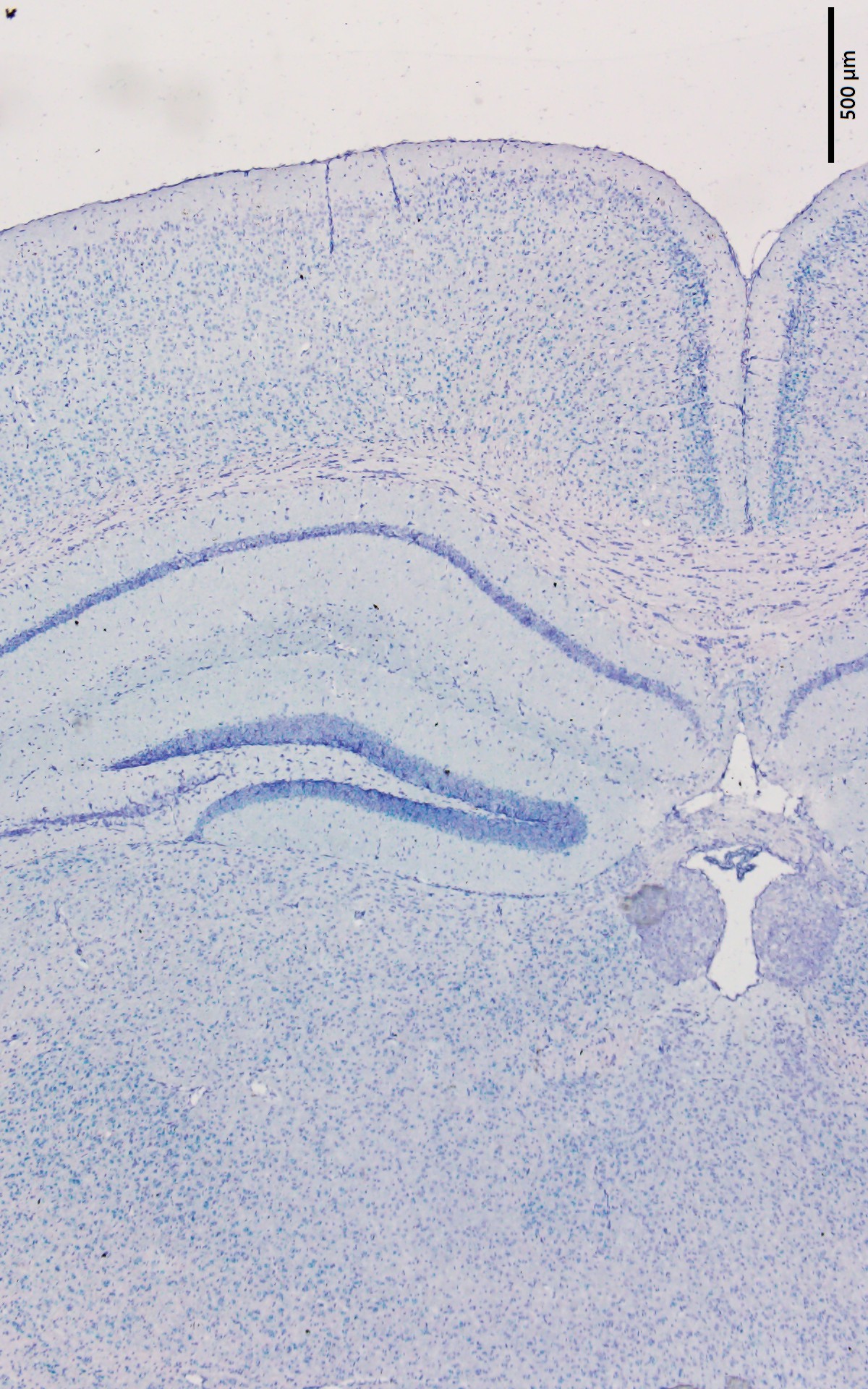

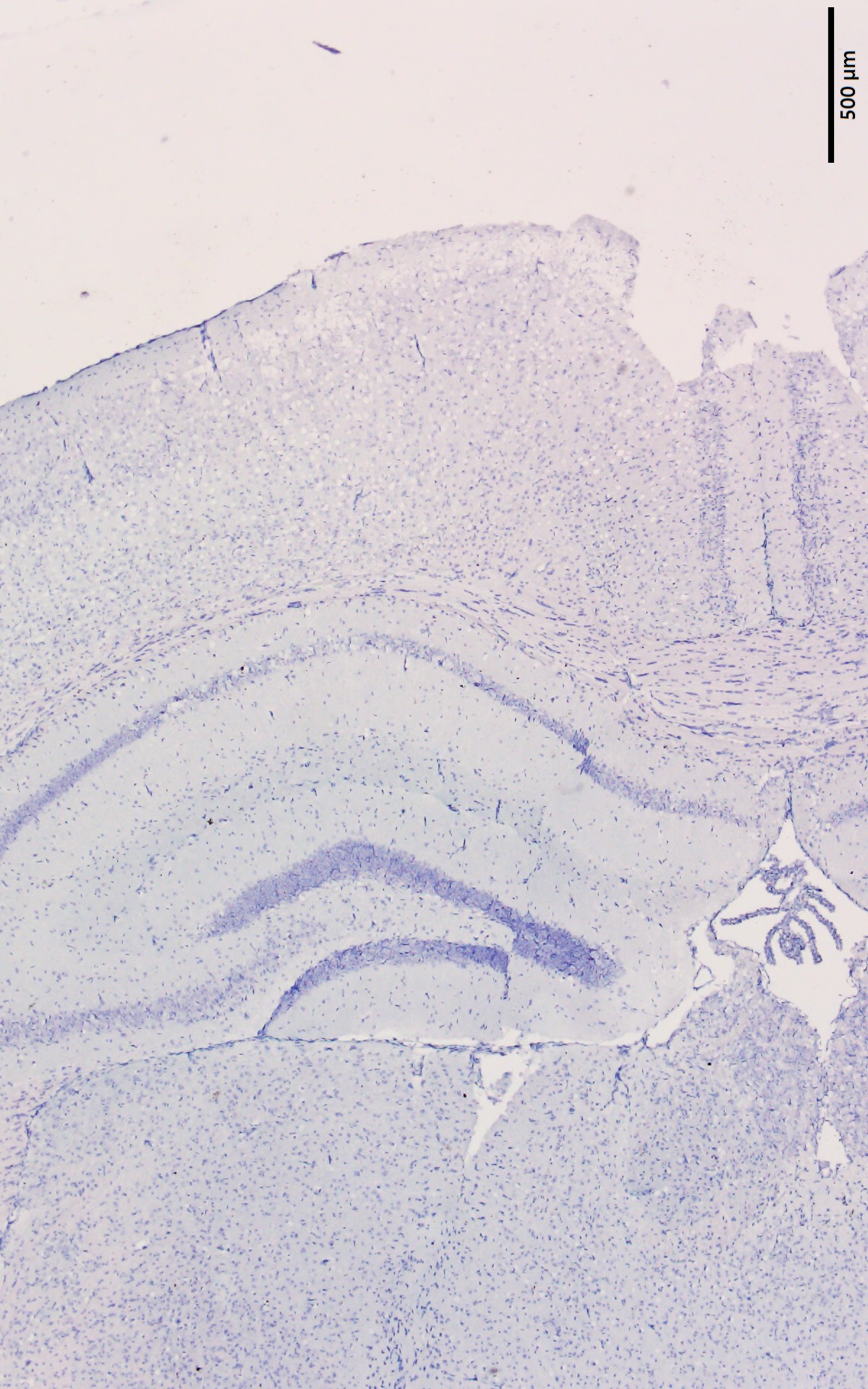

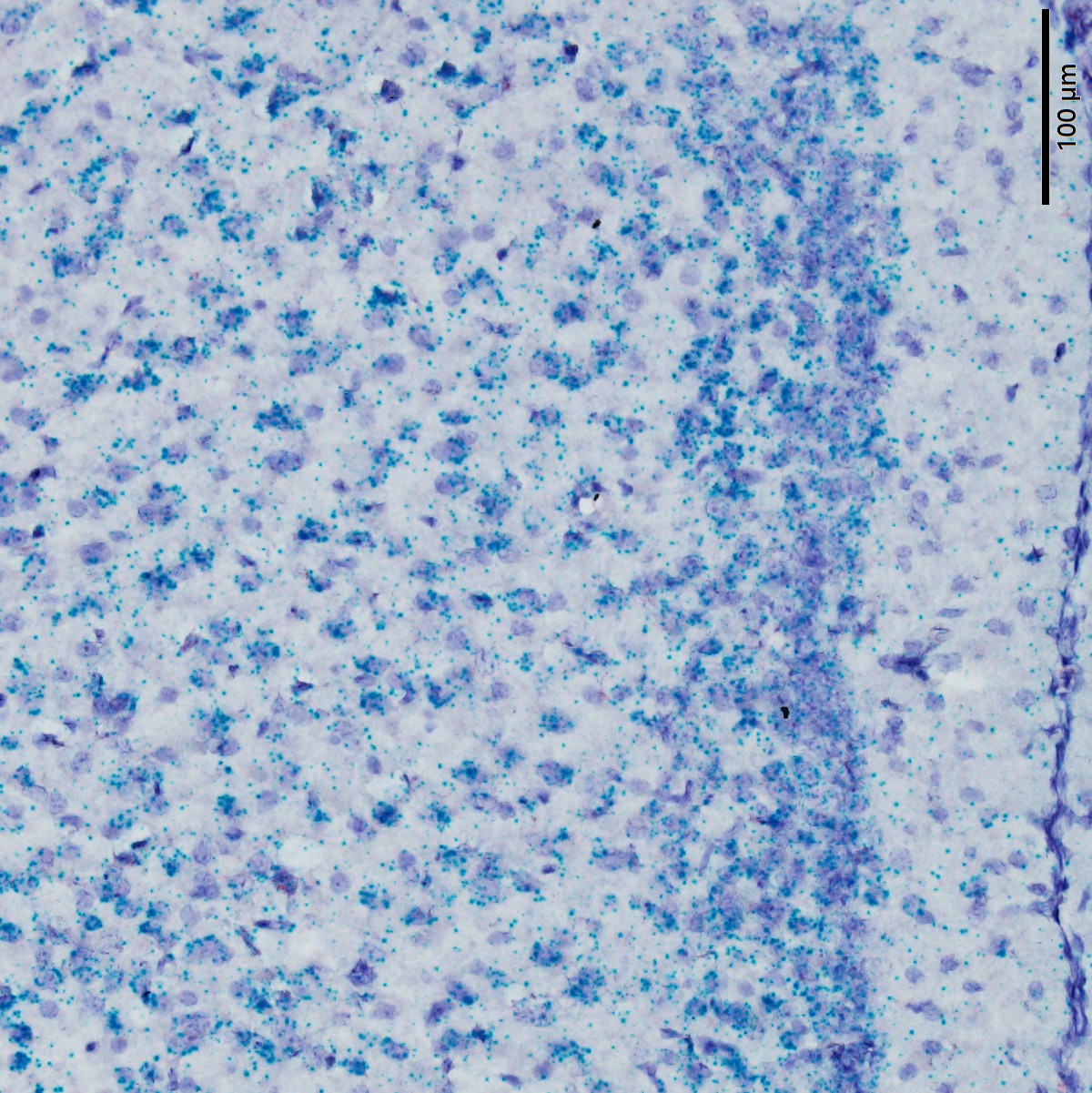

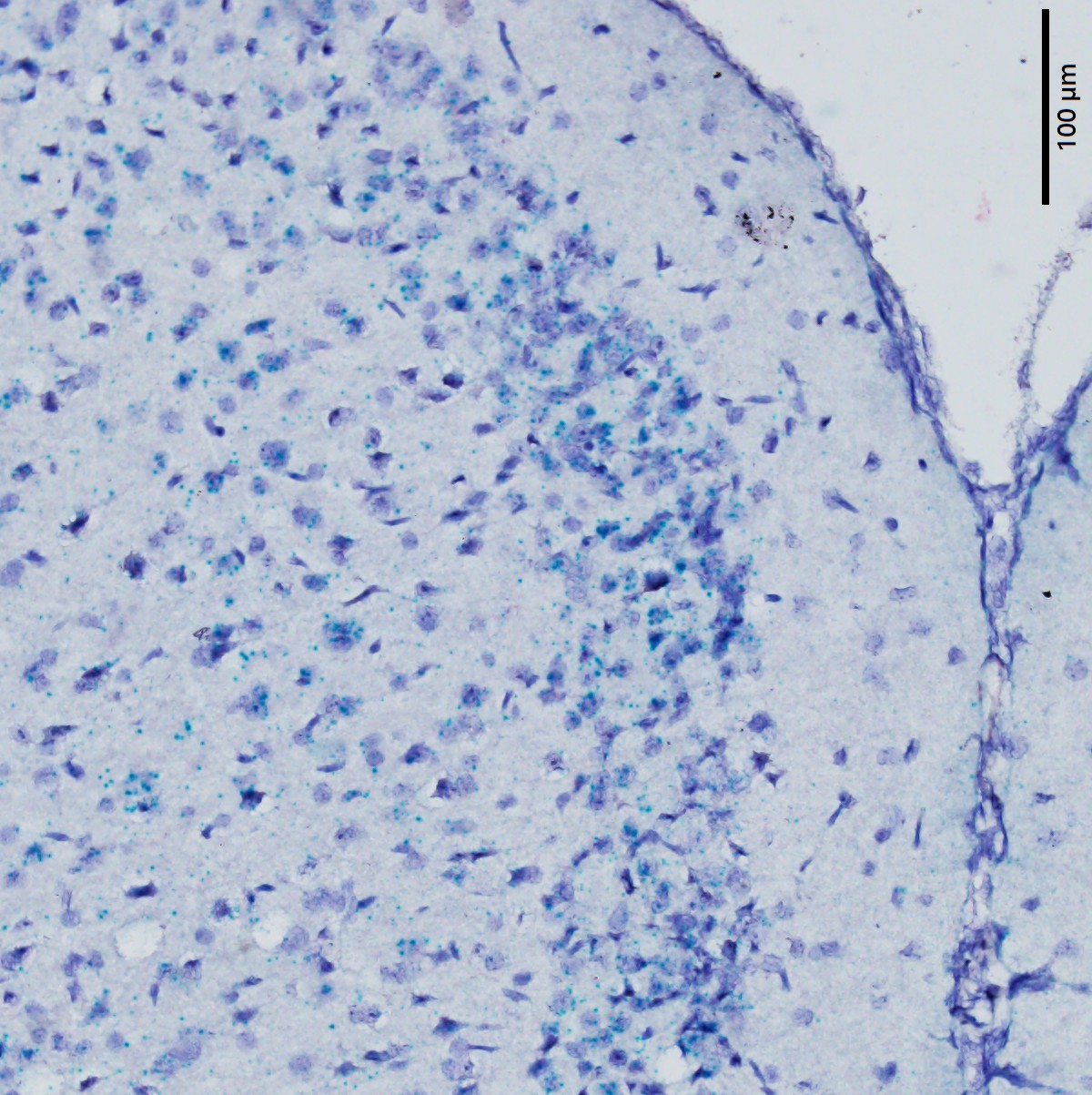

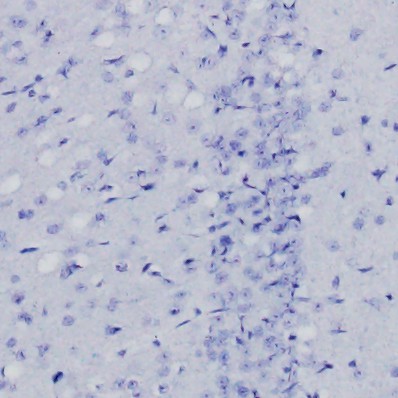

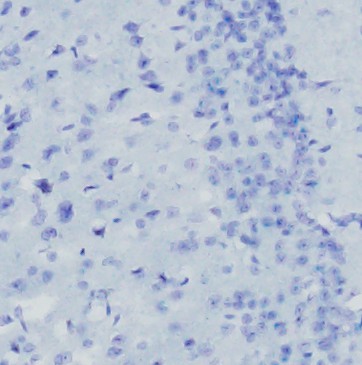


**Kir6.2 WT**

**Kir6.2 KO**

**Ins1Cre/0;Kir6.2flox/flox**

**NestinCre/0;Kir6.2flox/flox**

**B**


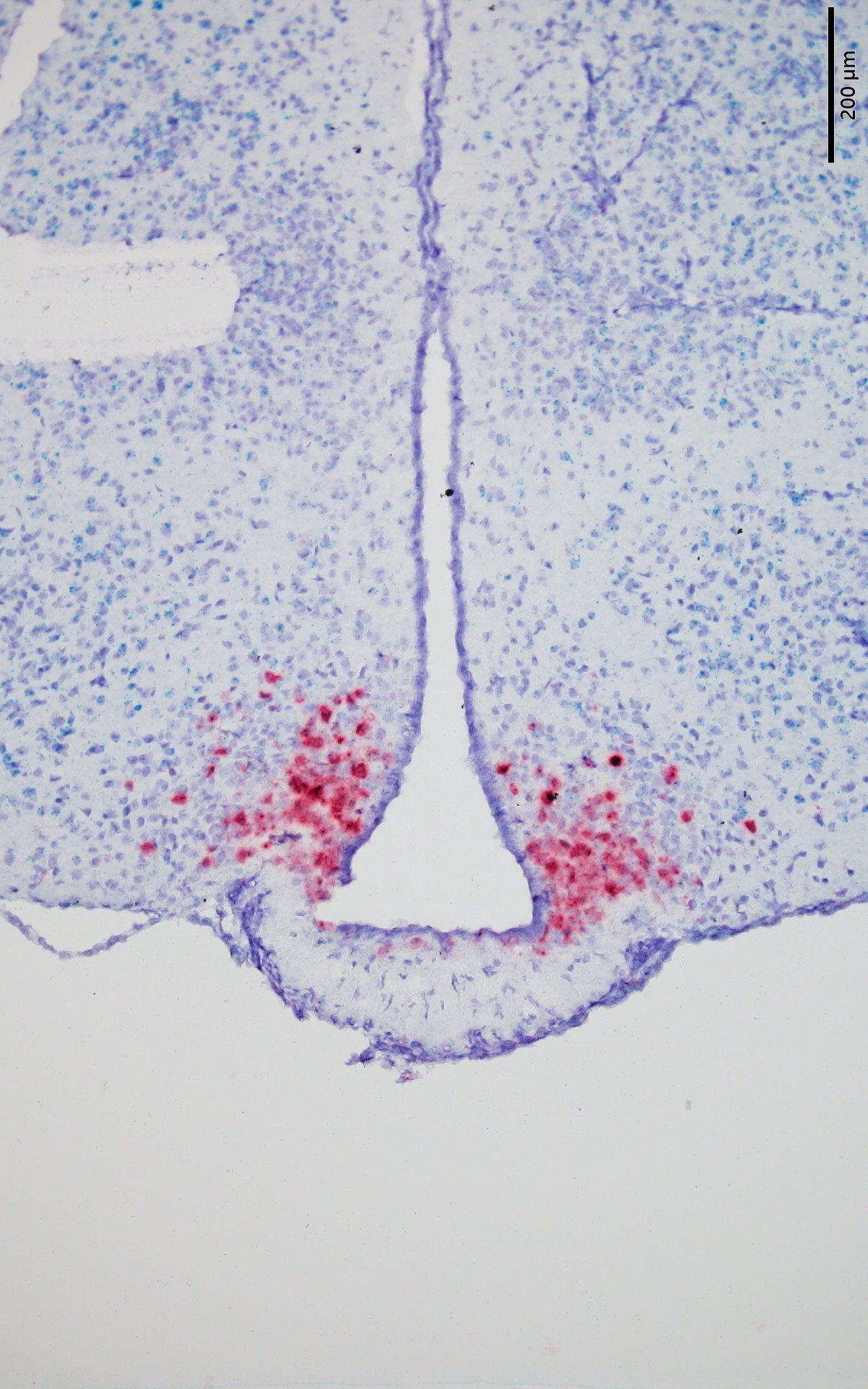

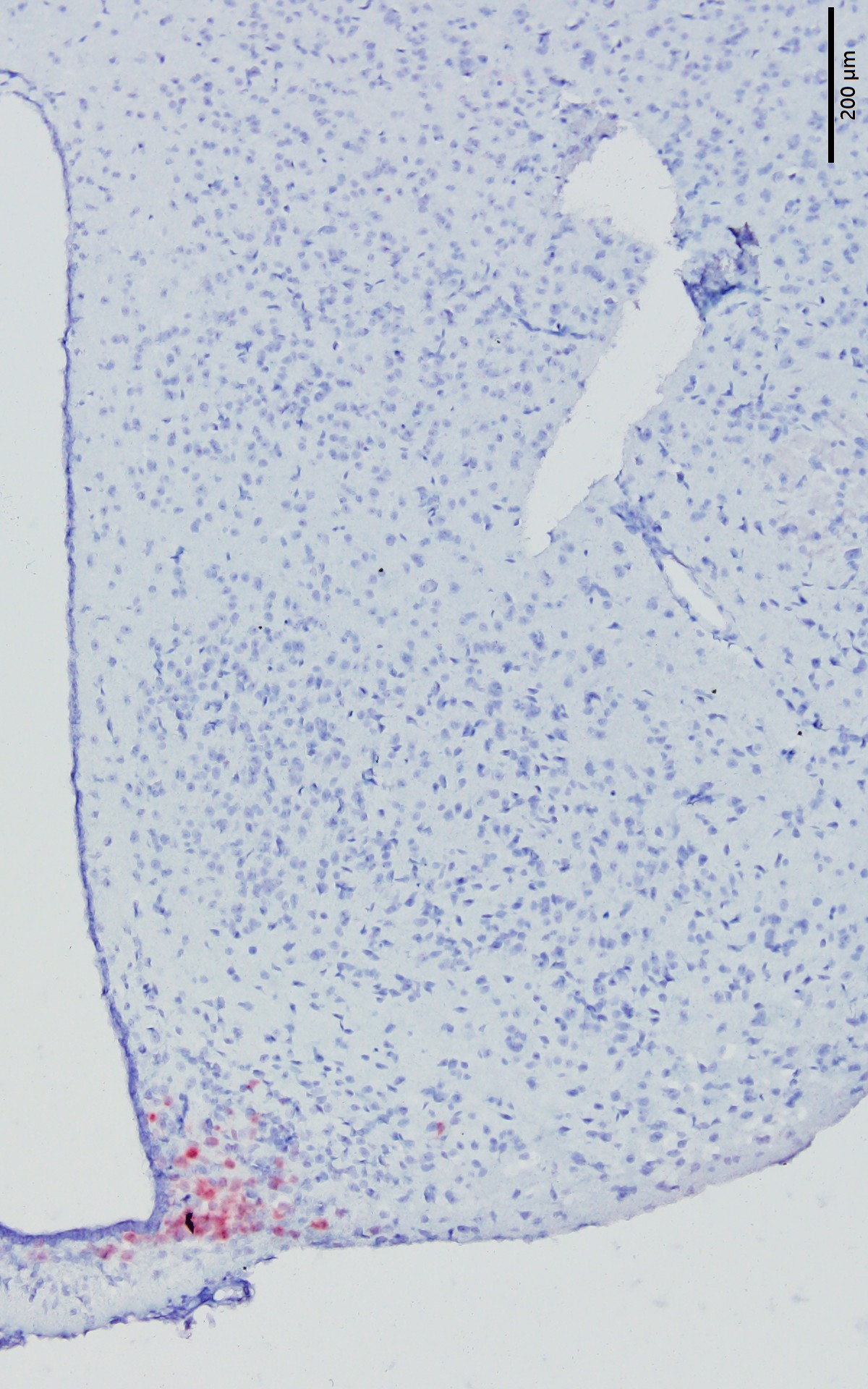

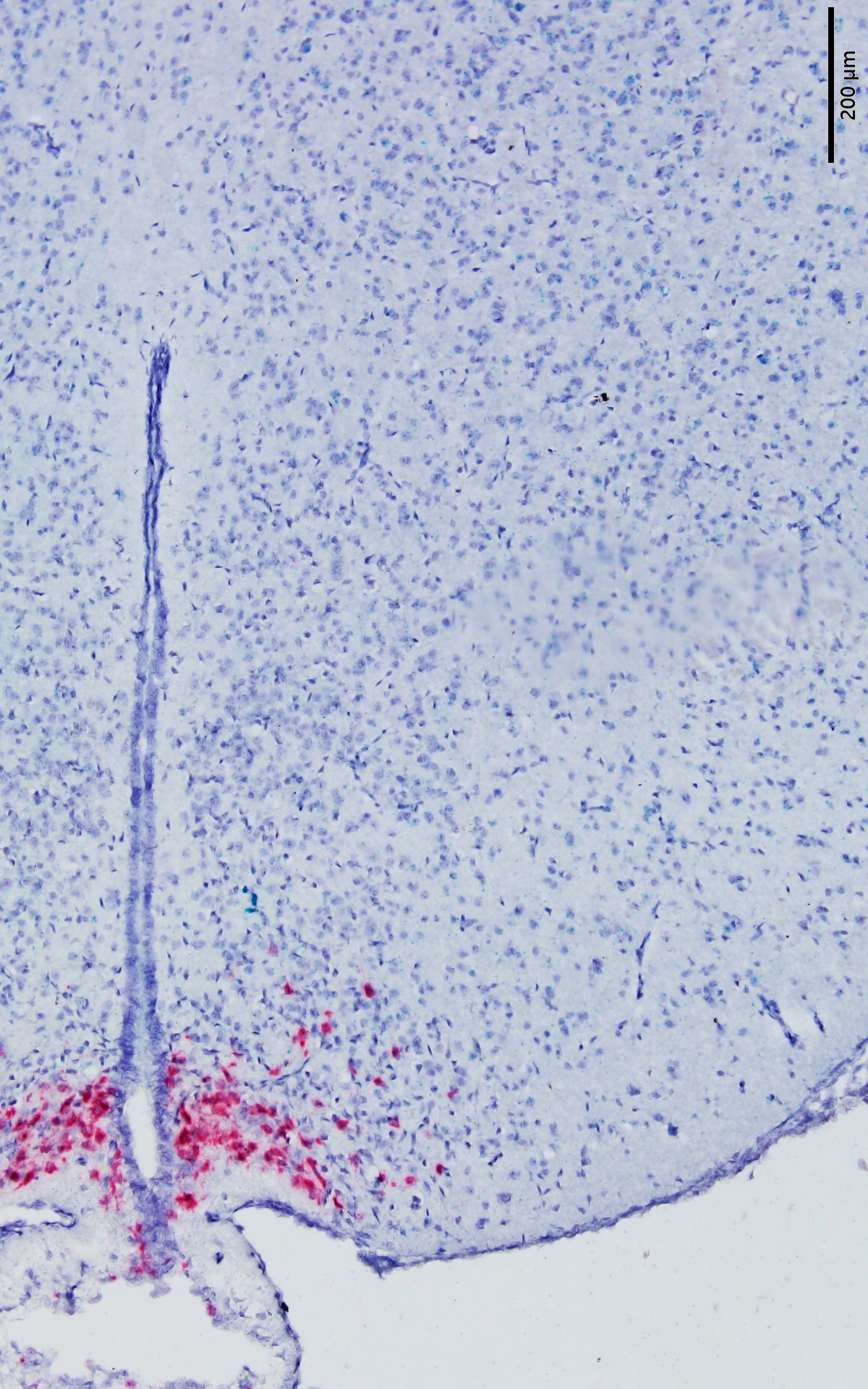

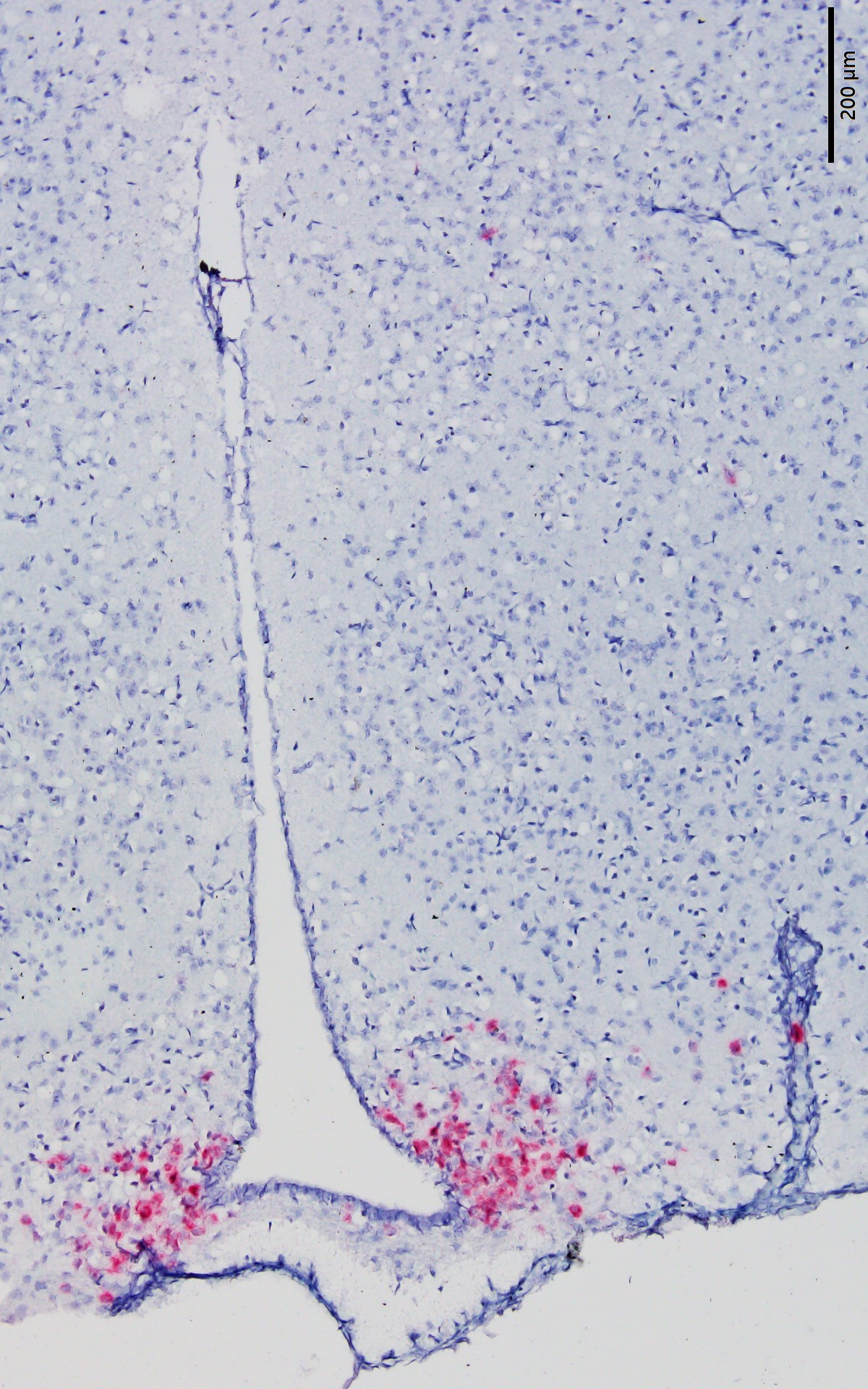

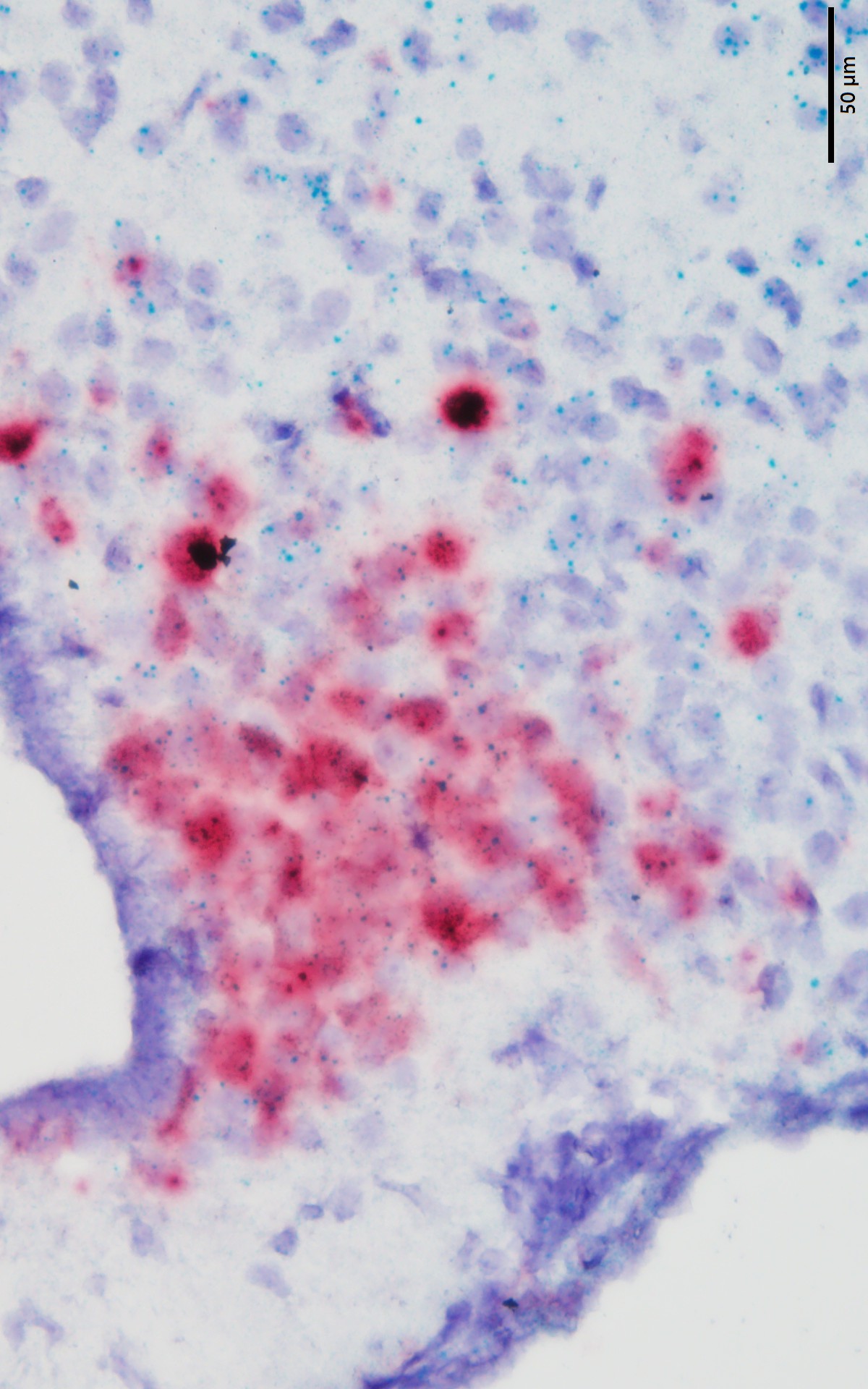

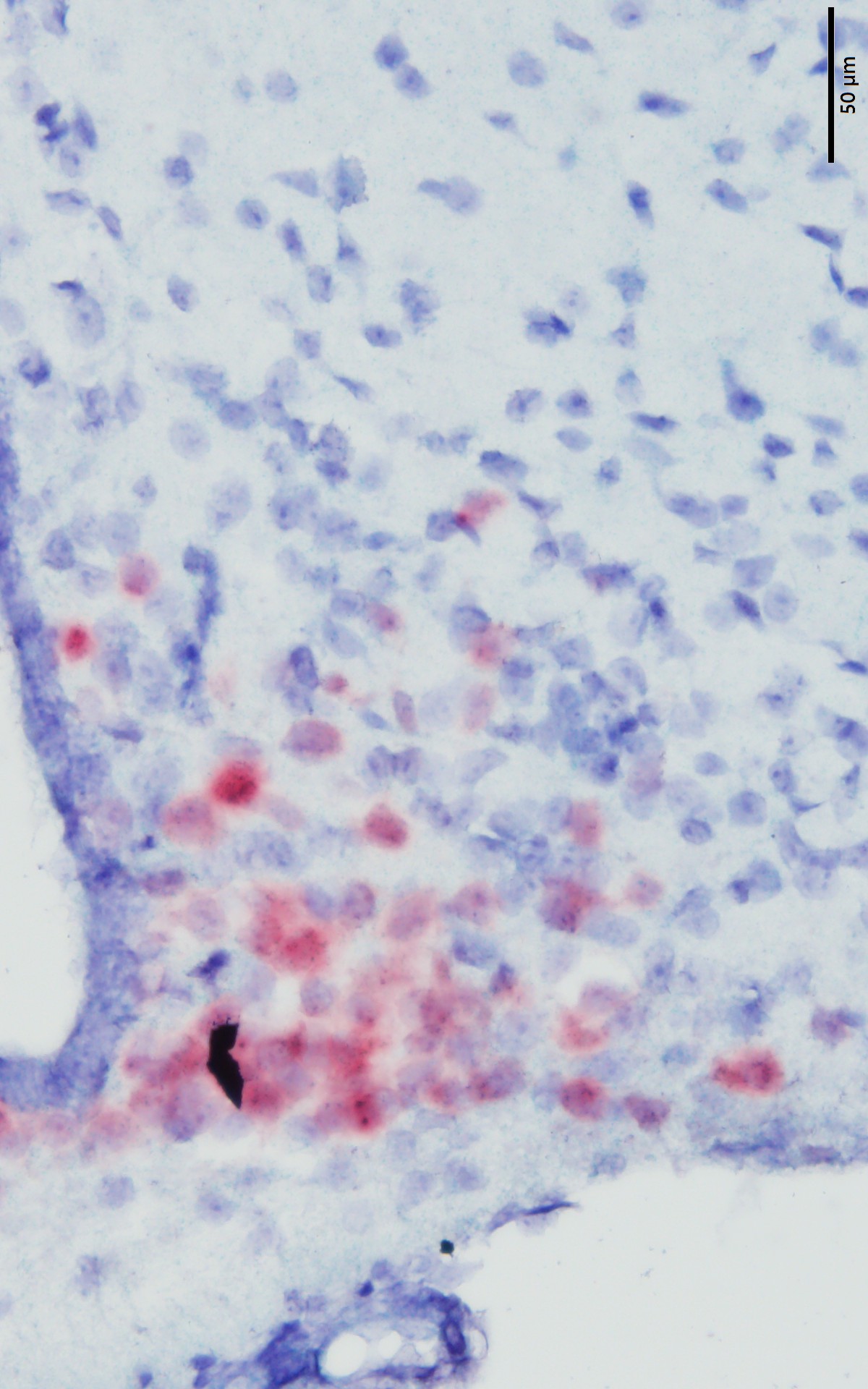

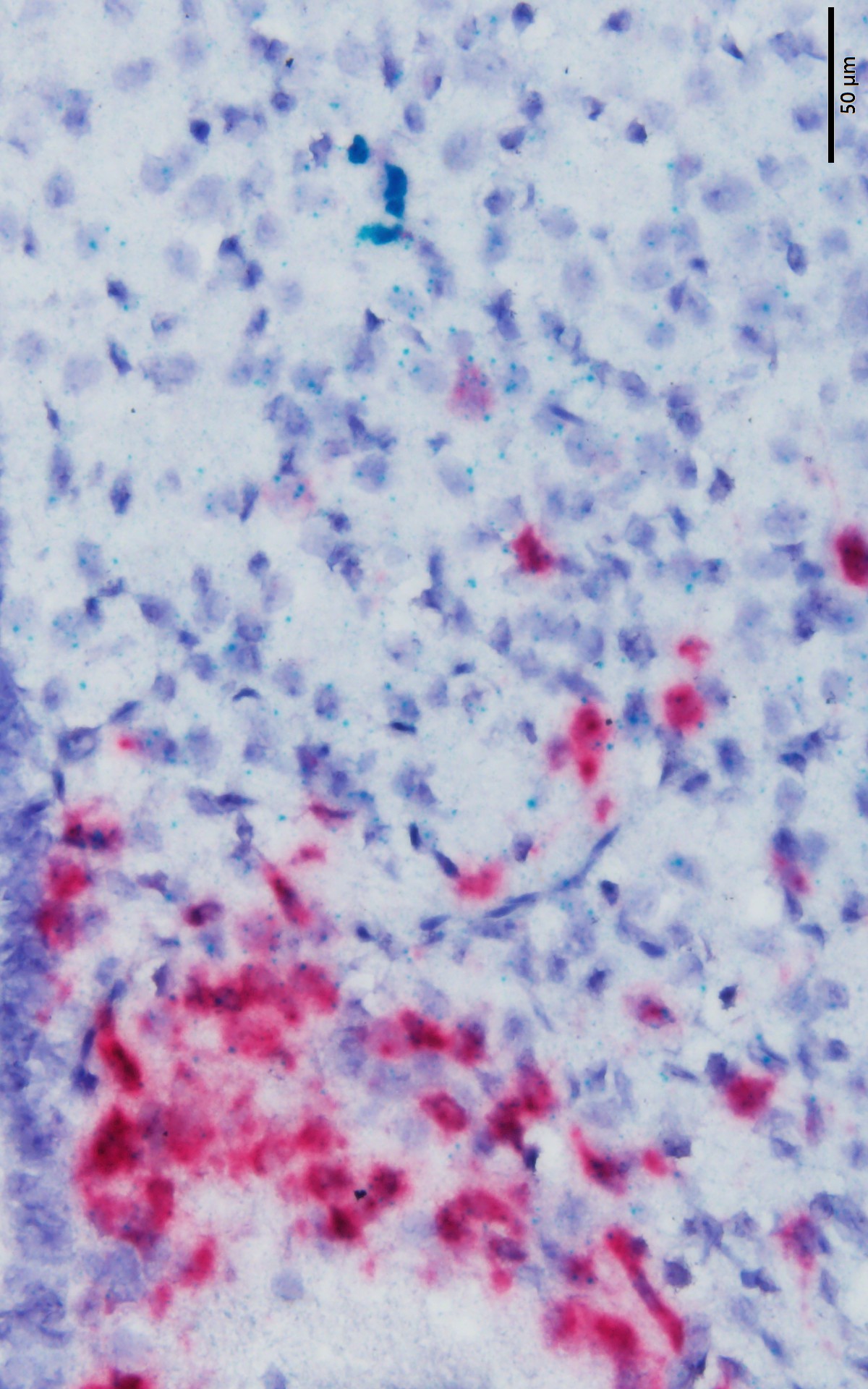

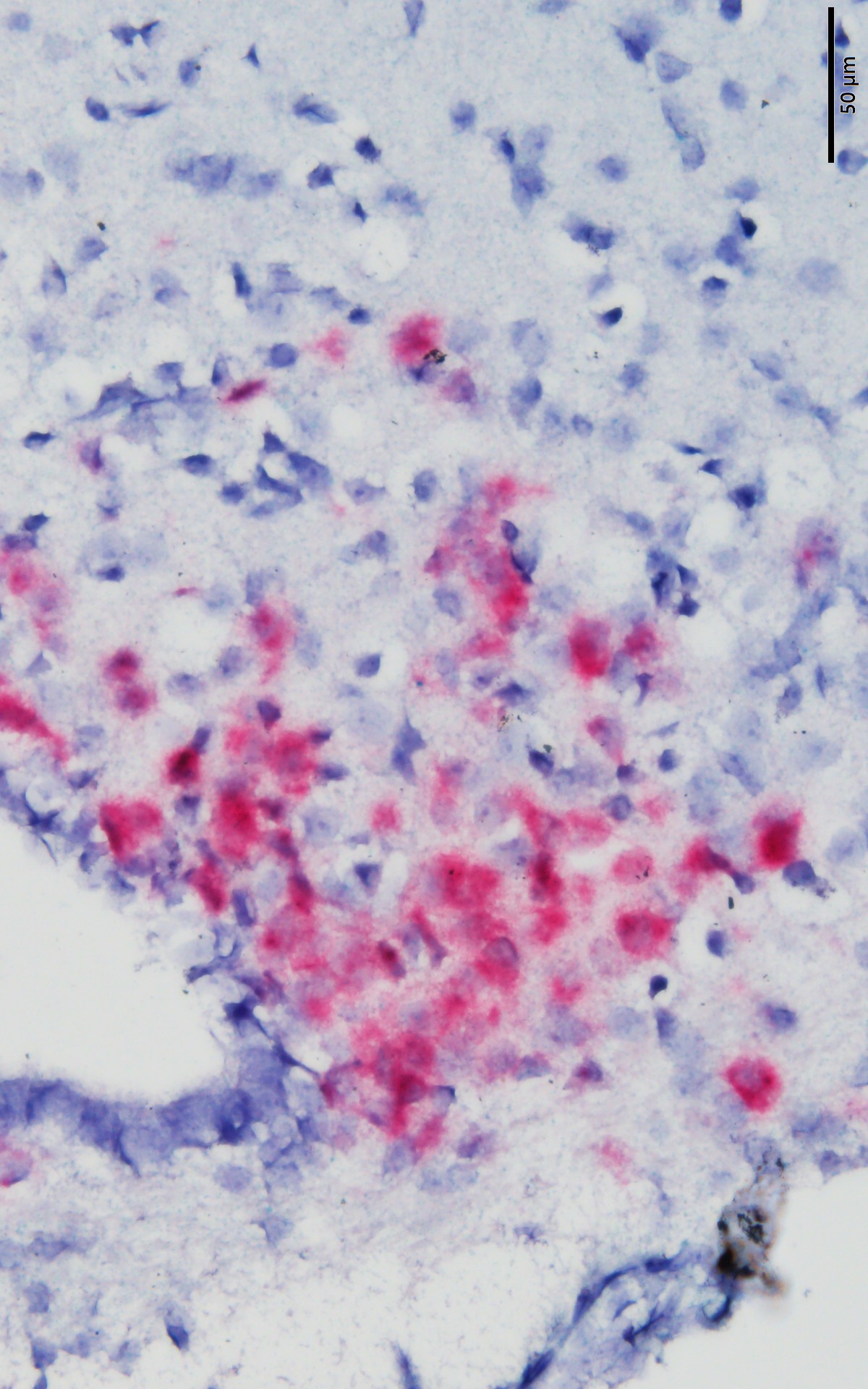


| **Kir6.2 WT** | **Kir6.2 KO** | **Ins1Cre/0;Kir6.2flox/flox** | **NestinCre/0;Kir6.2flox/flox** |
| --- | --- | --- | --- |

**C Kir6.2 WT Kir6.2 KO**

**Ins1Cre/0;Kir6.2flox/flox NestinCre/0;Kir6.2flox/flox**

| 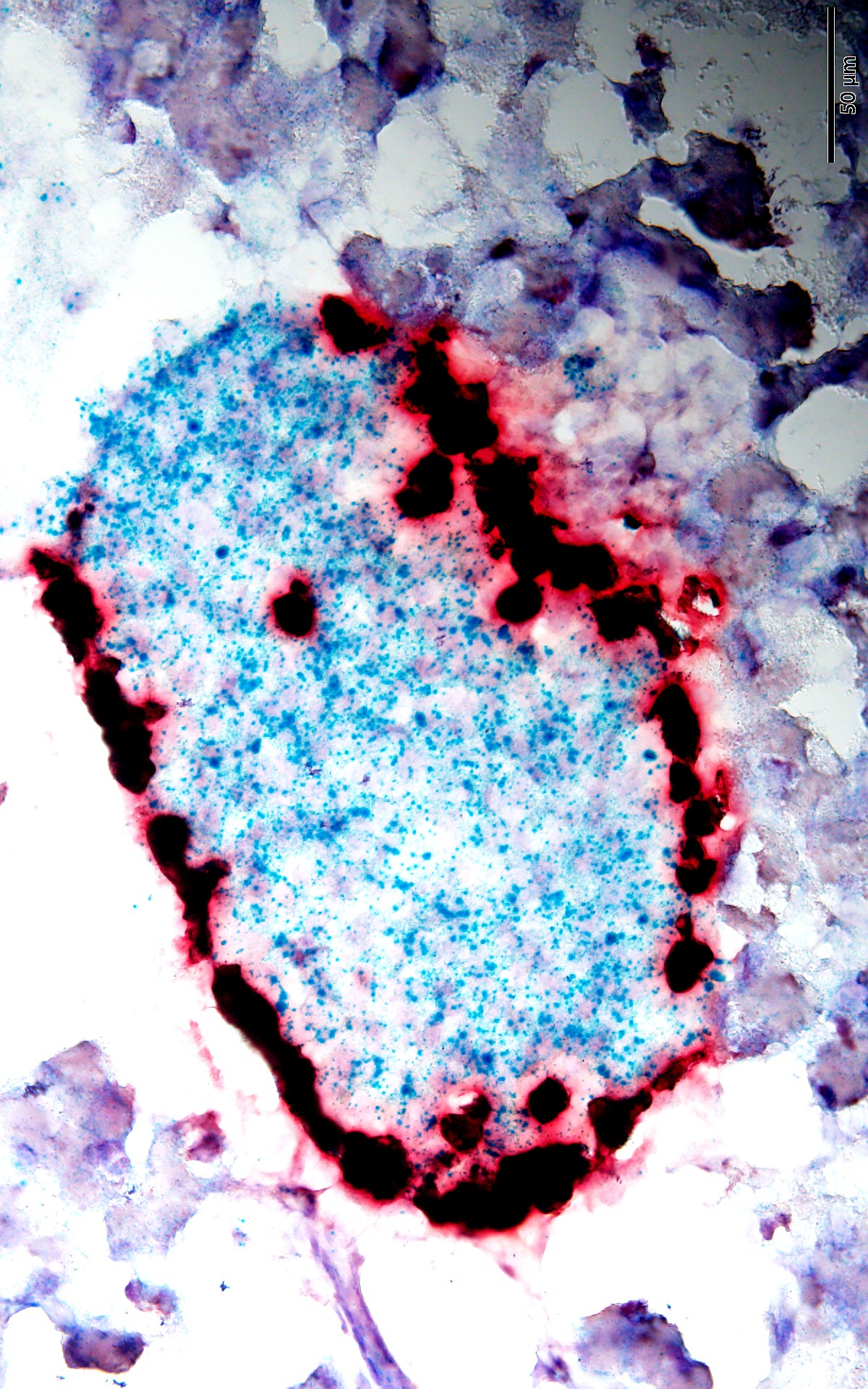 | 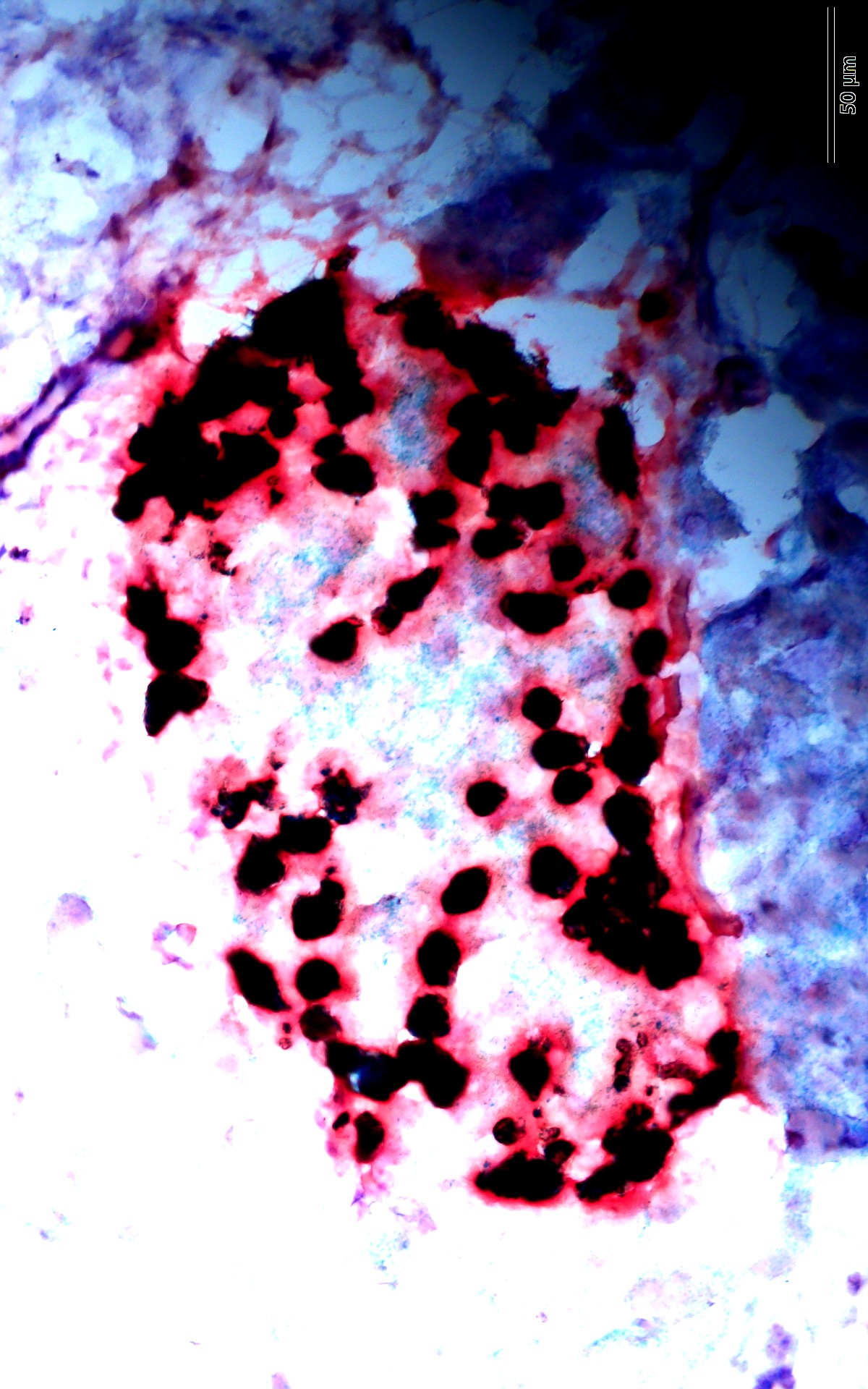 | 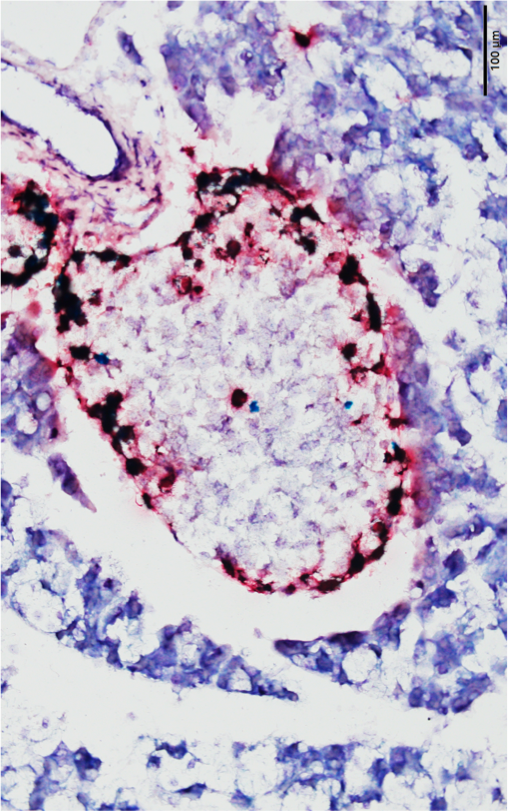 | 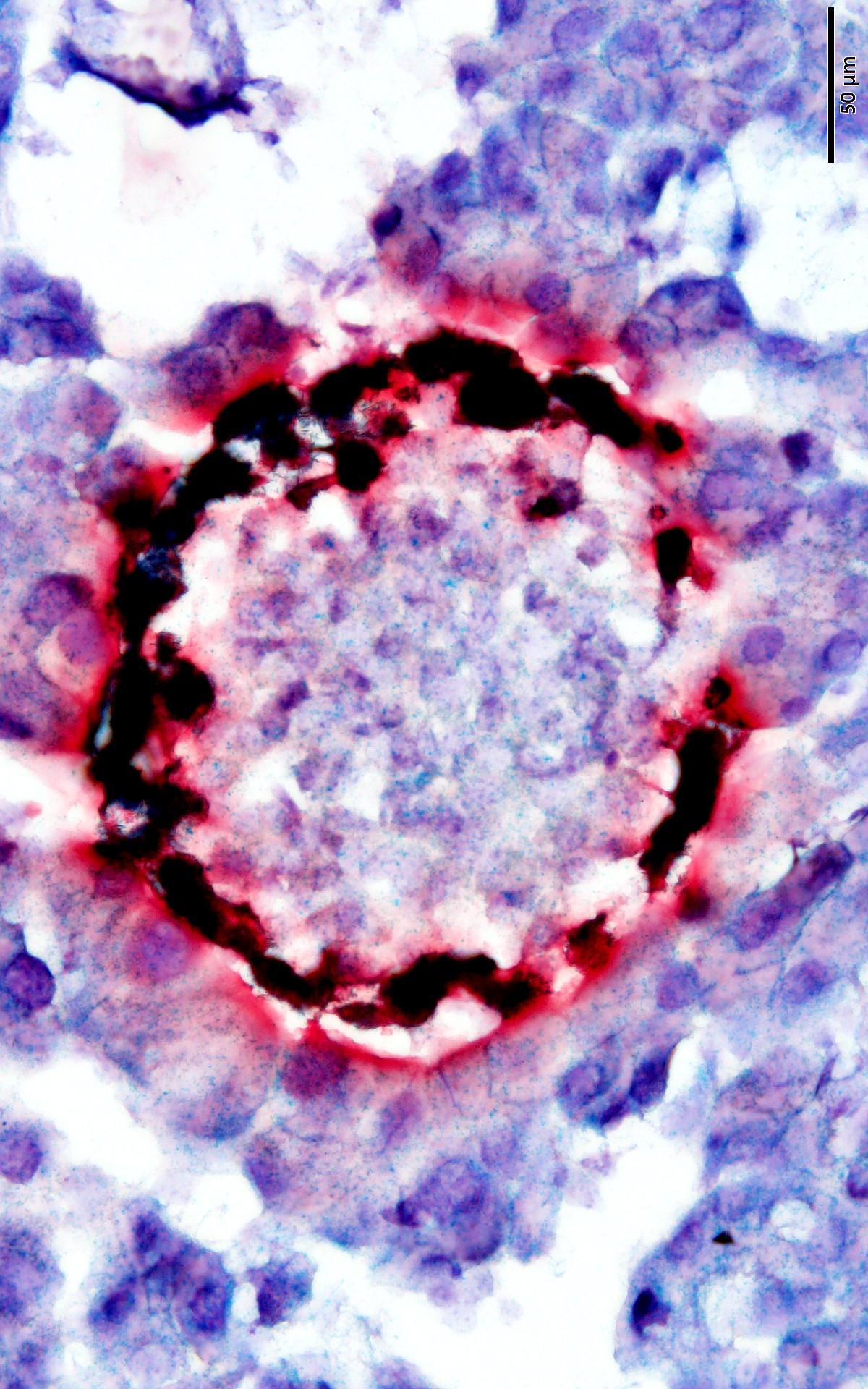 |
| --- | --- | --- | --- |

**Supplementary Figure 3: Validation of successful Kir6.2 knockout in target organs in different mouse strains. A-C:** RNAscope staining images for *Kcnj11* mRNA (*blue*) in cortex and hippocampus with higher magnification images of the retrosplenial granular cortex region (**A**), co-stained with *Agrp* mRNA (*red*) for anatomical reference in the arcuate nucleus (**B**), and co-stained with *Gcg* mRNA (*red*) for anatomical reference in the pancreatic islets (**C**) of WT, Kir6.2 KO, Ins1Cre/0;Kir6.2flox/flox, and NestinCre/0;Kir6.2flox/flox mice (from *left* to *right*). Counterstained with hematoxylin (*purple*). Scale bars, 500 μm (**A** large panels), 100 μm (**A** magnified *top left* square panels, **C** Ins1Cre/0;Kir6.2flox/flox panel), and 50 μm (**B**, **C** others).

**Alt text:** The validation of *Kcnj11* mRNA expression labeled in blue in target knockout regions, including brain cortex and hypothalamus, with pink *Agrp* signals as reference, shown in **A** and **B** and pancreatic islets, with pink *Gcg* signals as reference, shown in **C** prove successful knockout in targets and no off-target deletion in other regions.

**A B**

**40 40**

WT Kir6.2 KO

Ins1Cre/0;Kir6.2flox/+ Ins1Cre/0;Kir6.2flox/flox

**ns**

**ns**

**30 30**

**Body Weight (grams)**

**Body Weight (grams)**

**20 20**

**10 10**

**0**

**1 2 3 4 5**

**Age (months)**

**C**

**30**

NestinCre/0;Kir6.2flox/+ NestinCre/0;Kir6.2flox/flox

**0**

**1 2 3 4 5**

**Age (months)**

**20**

**ns**

**Body Weight (grams)**

**10**

**0**

**1 2 3 4 5**

**Age (months)**

**Supplementary Figure 4: Body weight of conditional Kir6.2 KO mouse strains were not affected. A**–**C:** Body weight measurements of WT and Kir6.2 KO (**A**), Ins1Cre/0;Kir6.2flox/+ and flox/flox (**B**), and NestinCre/0;Kir6.2flox/+ and flox/flox mice (**C**). WT: *n* = 6; Kir6.2 KO: *n* = 7; Ins1Cre/0;Kir6.2flox/+: *n* = 5; Ins1Cre/0;Kir6.2flox/flox: *n* = 10; NestinCre/0;Kir6.2flox/+: *n* = 8; NestinCre/0;Kir6.2flox/flox: *n* =5. Statistical comparisons were made by two-way ANOVA with Šídák’s correction. Data are represented as mean

± SEM. ns: not significant.

**Alt text:** Graphs of body weight monitored monthly up to 5 months in mice lacking both pancreatic and neuronal, only pancreatic, and only neuronal functional K_ATP_ channels shown in **A** to **C** show no alterations.

## Supplementary Methods Tamoxifen administration

Tamoxifen (Sigma-Aldrich) was dissolved in 100% EtOH, heated to 42°C until complete dissolution, to make a stock concentration of 100 mg/mL and stored at -20°C prior to use. Before use, stock solution was thawed to room temperature and sunflower seed oil from *Helianthus annuus* (Sigma-Aldrich) was added and mixed further to reach a final concentration of 10 mg/mL. A final dose of 40 mg/kg was administered to αMHCMCM/0;Kir6.2flox/+ and flox/flox mice via oral gavage for 5 consecutive days. IPGTT and ITT were re-tested 2 weeks after the final day of tamoxifen administration.

**A**

**800**

**Blood glucose (mg/dL)**

**600**

**400**

**200**

**B**

**250**


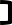


MCKCre/0;Kir6.2flox/+ MCKCre/0;Kir6.2flox/flox


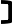


MCKCre/0;Kir6.2flox/+ MCKCre/0;Kir6.2flox/flox

**Blood glucose (mg/dL)**

**200**

**150**

**100**

ns

ns

**50**

**0**

**C 800**

**Blood glucose (mg/dL)**

**600**

**400**

**200**

**0 30 60 90 120 150**

**Time (min)**

αMHC^MCM/0^;Kir6.2^flox/+^ (Before) αMHC^MCM/0^;Kir6.2^flox/flox^ (Before) αMHC^MCM/0^;Kir6.2^flox/+^ (After)


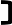


αMHC^MCM/0^;Kir6.2^flox/flox^ (After)

**0**

**300**

**Blood glucose (mg/dL)**

**D**

**200**

**100**

**0 30 60 90 120**

**Time (min)**

**0**

ns

ns

**0 30 60 90 120 150**

**Time (min)**

**0**

**0 30 60 90 120**

αMHC^MCM/0^;Kir6.2^flox/+^ (Before) αMHC^MCM/0^;Kir6.2^flox/flox^ (Before) αMHC^MCM/0^;Kir6.2^flox/+^ (After)

αMHC^MCM/0^;Kir6.2^flox/flox^ (After)

**Time (min)**

**E F**

αMHC^MCM/0^;Kir6.2^flox/+^ (Before) αMHC^MCM/0^;Kir6.2^flox/flox^ (Before) αMHC^MCM/0^;Kir6.2^flox/+^ (After)

αMHC^MCM/0^;Kir6.2^flox/flox^ (After)

**800**


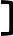


αMHC^MCM/0^;Kir6.2^flox/+^ (Before) αMHC^MCM/0^;Kir6.2^flox/flox^ (Before) αMHC^MCM/0^;Kir6.2^flox/+^ (After)

αMHC^MCM/0^;Kir6.2^flox/flox^ (After)

**250**

**600**

**Blood glucose (mg/dL)**

**400**

**200**

**200**

**150**

**Blood glucose (mg/dL)**

**100**

ns

**50**

**0**

ns

**0 30 60 90 120 150**

**Time (min)**

**0**

**0 30 60 90 120**

**Time (min)**

## Supplementary Figure 5: Glucose tolerance and insulin sensitivity are unaffected in mice without functional cardiac or muscular K_ATP_ channels.

**A**–**F:** Blood glucose levels during IPGTT and ITT in MCKCre/0;Kir6.2flox/+,flox/flox (IPGTT: *P* = 0.8295, F (1, 19) = 0.04768; ITT: *P* = 0.5995, F (1, 18) = 0.2858) (**A**,**B**) and αMHCMCM/0;Kir6.2flox/+,flox/flox mice before

and 2 weeks after receiving tamoxifen (IPGTT: *P* = 0.5351, F (3, 49) = 0.7368; ITT: *P* = 0.3069, F (3, 49)

= 1.236) (**C**,**D**) or vehicle administration (IPGTT: *P_group_* = 0.0146, F (3, 24) = 4.295; *P_time_ _×_ _group_* = 0.1298, F (15, 120) = 1.463; ITT: *P* = 0.5872, F (3, 24) = 0.6558) (**E**,**F**). MCKCre/0;Kir6.2flox/+: *n* = 7 (**A**), 6 (**B**);

MCKCre/0;Kir6.2flox/flox: *n* = 14 (**A**,**B**); αMHCMCM/0;Kir6.2flox/+: *n* = 17 (**C**,**D** before), 13 (**C**,**D** after), 7 (**E**,**F**); αMHCMCM/0;Kir6.2flox/flox: *n* = 13 (**C** before), 10 (**C** after), 12 (**D** before), 11 (**D** after), 8 (**E**,**F** before), 6 (**E**,**F** after). Statistical comparisons were made by two-way ANOVA with Šídák’s (**A**,**B**) and Tukey’s correction (**C**–**F**). Data are represented as mean ± SEM. ns: not significant.

**Alt text:** Graphs depicting glucose tolerance and insulin sensitivity in mice lacking functional K_ATP_ channels in skeletal or cardiac myocytes labeled from **A** to **F** show that neither is altered in any of the mice.

**B**


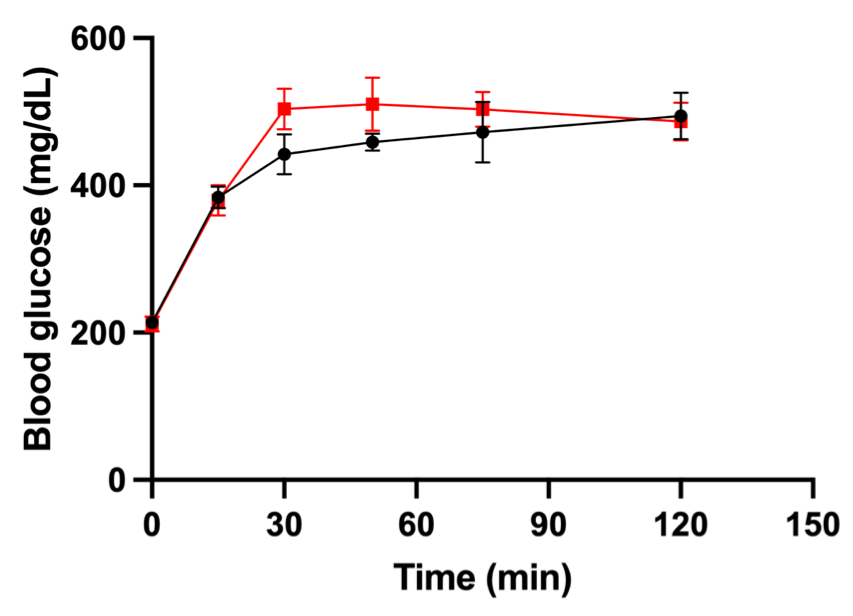

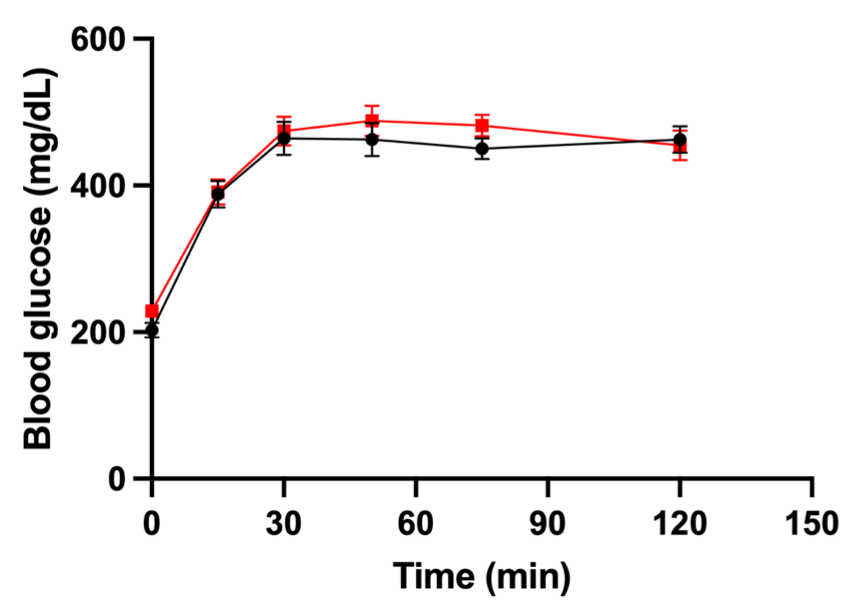


**A**

AgRPCre/+;Kir6.2flox/+

AgRPCre/+;Kir6.2flox/flox

**C**

MCKCre/0;Kir6.2flox/+

MCKCre/0;Kir6.2flox/flox


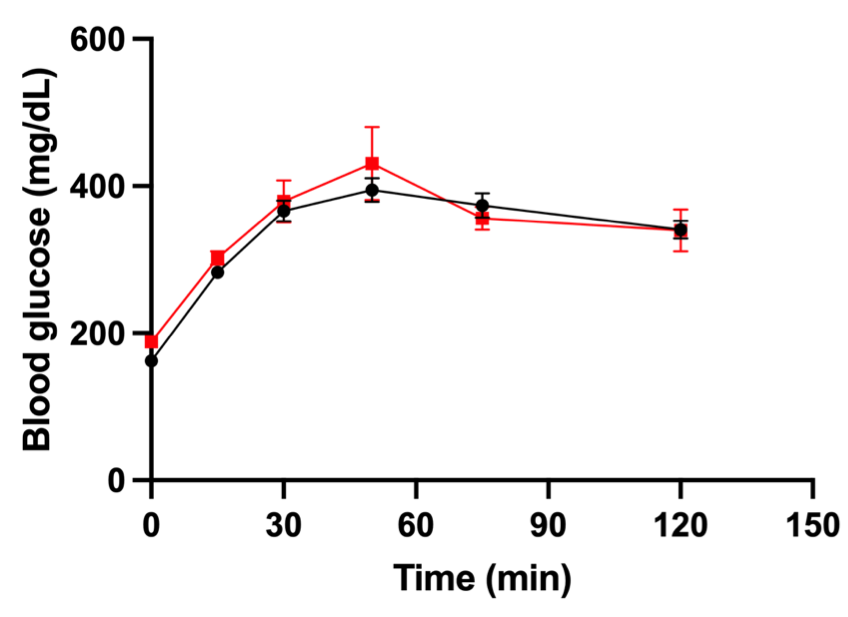


THCre/0;Kir6.2flox/+ THCre/0;Kir6.2flox/flox

ns

ns

ns

## Supplementary Figure 6: Glycopenia sensitivity is unaffected in mice without functional neuronal or muscular K_ATP_ channels.

**A**–**C:** Blood glucose levels during 2DG-induced glycopenia assessment in AgRPCre/+;Kir6.2flox/+,flox/flox (**A**), THCre/0;Kir6.2flox/+,flox/flox (**B**), and MCKCre/0;Kir6.2flox/+,flox/flox mice (**C**). AgRPCre/+;Kir6.2flox/+: *n* = 12; AgRPCre/+;Kir6.2flox/flox: *n* = 11; THCre/0;Kir6.2flox/+: *n* = 11; THCre/0;Kir6.2flox/flox: *n* = 3; MCKCre/0;Kir6.2flox/+: *n* = 3; MCKCre/0;Kir6.2flox/flox: *n* = 6. Statistical comparisons were made by two-way ANOVA with Šídák’s correction. Data are represented as mean ± SEM. ns: not significant.

**Alt text:** Graphs depicting the ability to sense glycopenia, a state of systemic glucose shortage, in mice lacking functional K_ATP_ channels in AgRP neurons, TH neurons, and skeletal myocytes labeled from **A** to **C** show no difference.


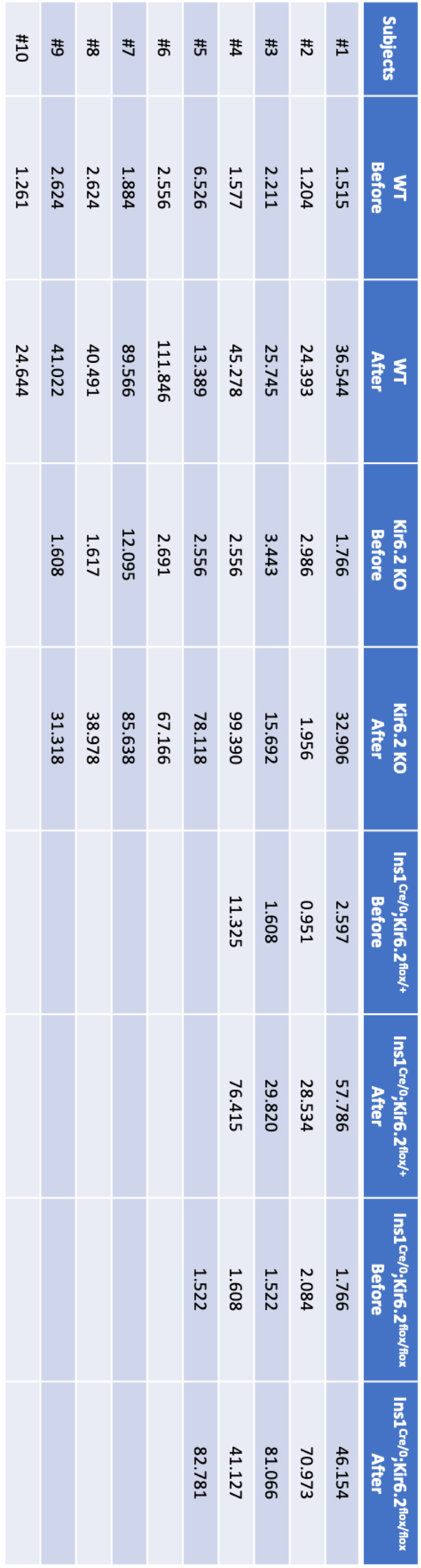


## Supplementary Table 1: Individual plasma glucagon concentration levels before and 30 minutes after 2DG-induced glycopenia.

The individual plasma glucagon concentration values (pmol/L) measured in WT, Kir6.2 KO, Ins1Cre/0;Kir6.2flox/+, and Ins1Cre/0;Kir6.2flox/flox mice before (labeled: “before”) and 30 minutes after 2DG injection (labeled: “after”). These datasets are used for **Figure 3D**.


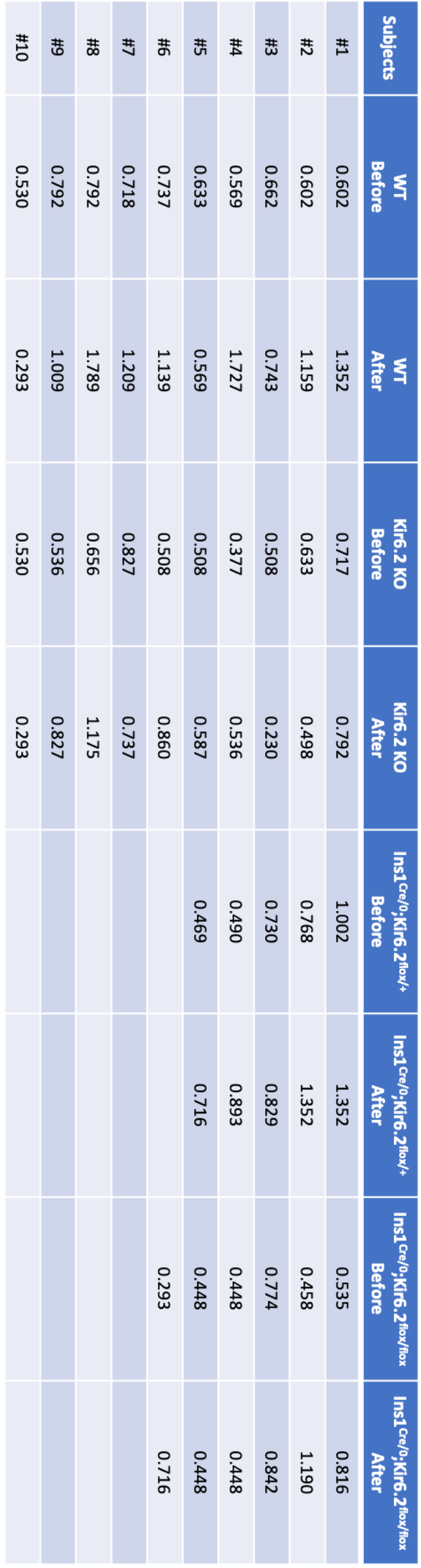


## Supplementary Table 2: Individual plasma insulin concentration levels before and 30 minutes after 2DG-induced glycopenia.

The individual plasma insulin concentration values (ng/mL) measured in WT, Kir6.2 KO, Ins1Cre/0;Kir6.2flox/+, and Ins1Cre/0;Kir6.2flox/flox mice before (labeled: “before”) and 30 minutes after 2DG injection (labeled: “after”). These datasets are used for **Figure 3E**.

**A**


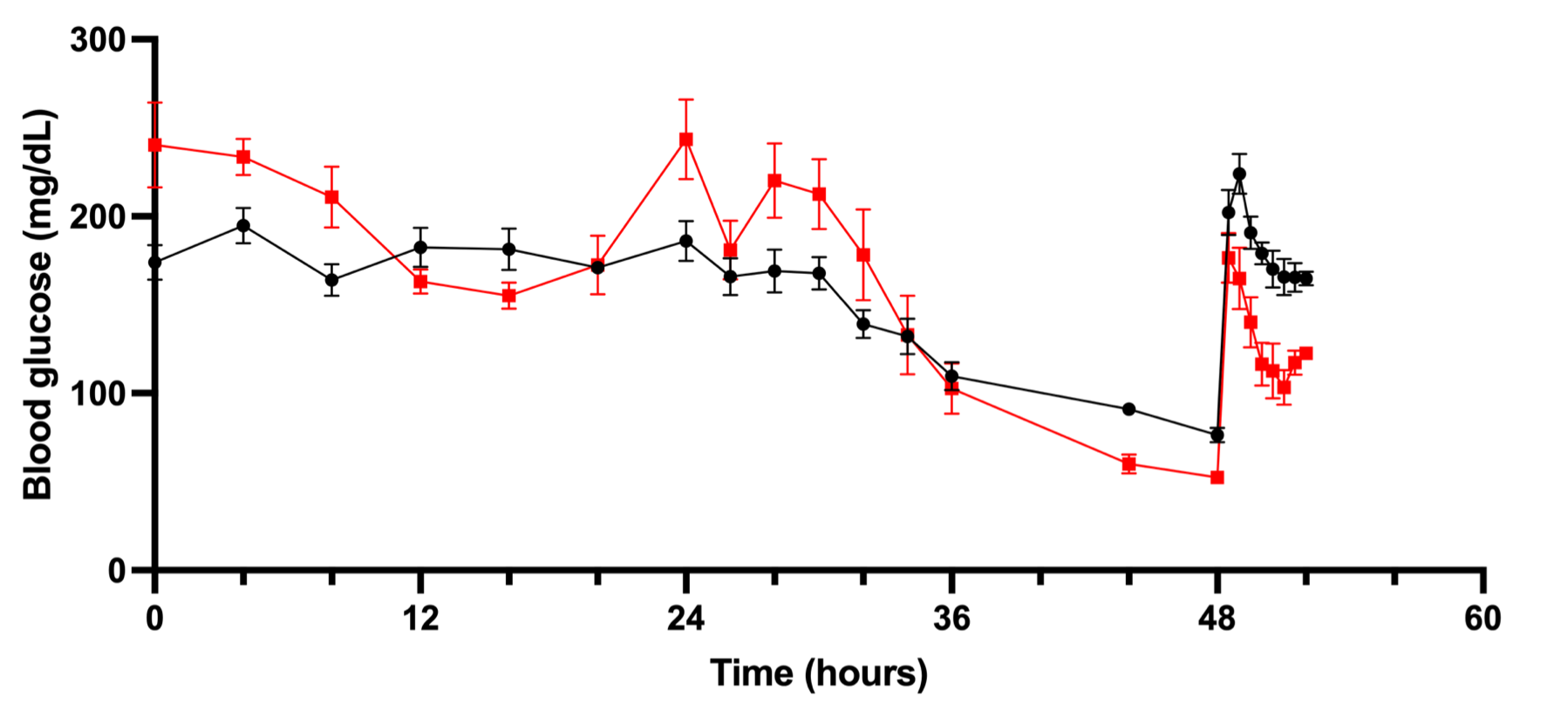

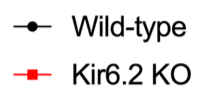


**re-feeding**

**(*P* = 0.0003)**

**(*P* = 0.0238)**

***ad libitum* (*P* = 0.0356)**

**(*P* = 0.1351)**

******

******

**fasting**

= dark cycle

= light cycle

**B**


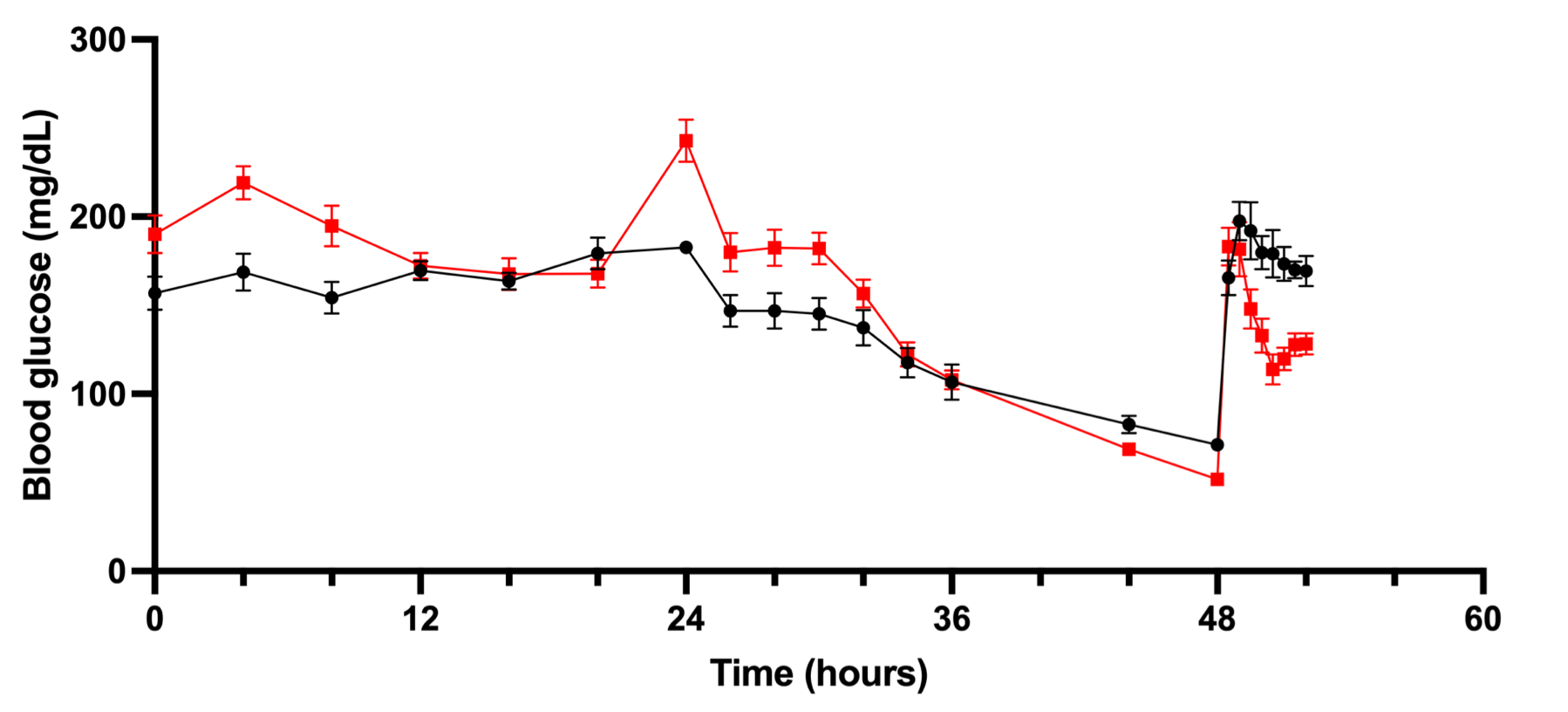

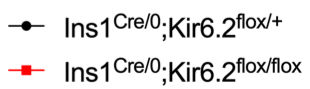


**re-feeding**

**(*P* = 0.0027)**

**(*P* = 0.0446)**

***ad libitum* (*P* = 0.0069)**

**(*P* = 0.0334)**

**fasting**

= dark cycle

= light cycle


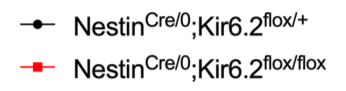

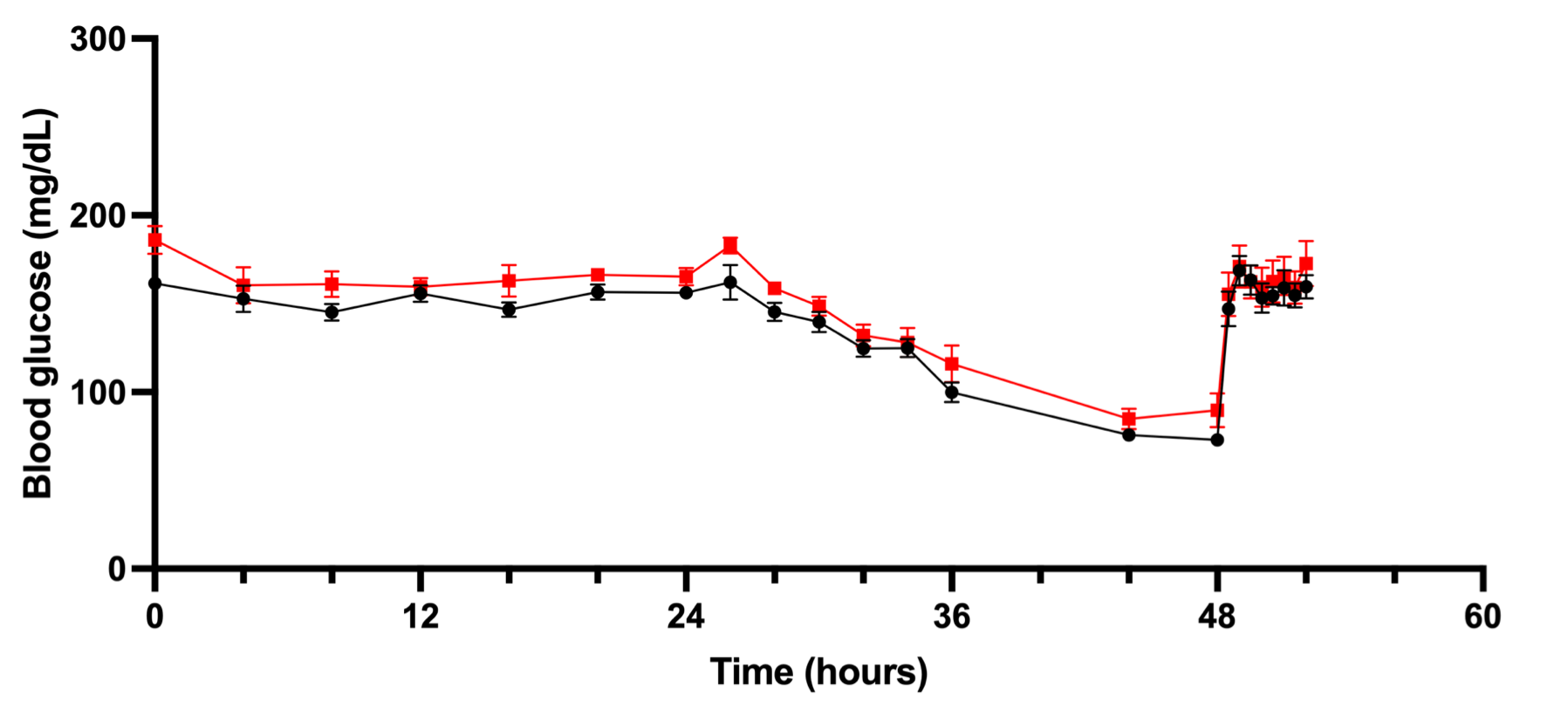
**C**

*******

**re-feeding**

**(*P* = 0.5045)**

***ad libitum* (*P* = 0.0097)**

**fasting (*P* = 0.0802)**

= dark cycle

= light cycle

**Supplementary Figure 7: K_ATP_ channels in pancreatic β cells act as the systemic glucostat. A**–**C:** 3-stage monitoring of blood glucose levels in WT vs Kir6.2 KO (**A**), Ins1Cre/0;Kir6.2flox/+ vs flox/flox (**B**), and NestinCre/0;Kir6.2flox/+ vs flox/flox mice (**C**). WT: *n* = 6; Kir6.2 KO: *n* = 6; Ins1Cre/0;Kir6.2flox/+: *n* = 6; Ins1Cre/0;Kir6.2flox/flox: *n* = 14; NestinCre/0;Kir6.2flox/+: *n* = 11; NestinCre/0;Kir6.2flox/flox: *n* = 7. Statistical comparisons were made by two-way ANOVA with Šídák’s correction followed by multiple comparisons. Data are represented as mean ± SEM. **P*

< 0.05, ***P* < 0.01, ****P* < 0.001, *****P* < 0.0001, ns: not significant.

**Alt text:** Graphs depicting blood glucose fluctuations when mice lacking both pancreatic and neuronal, only pancreatic, and only neuronal functional K_ATP_ channels are freely eating, fasted for prolonged durations, and refed after prolonged fasting labeled respectively from **A** to **C** show instability of blood glucose levels in mice lacking functional pancreatic K_ATP_ channels.


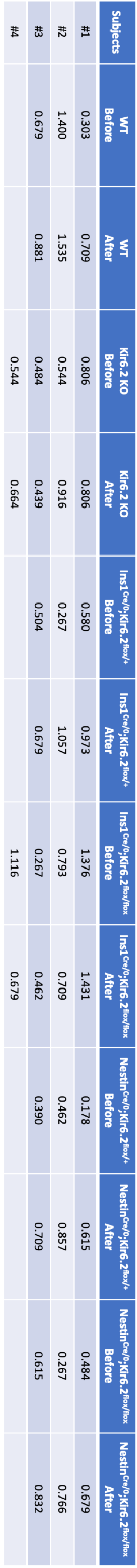


## Supplementary Table 3: Individual plasma insulin concentration levels after 24-hour fasting and after 30-minute re-feeding.

The individual plasma insulin concentration values (ng/mL) measured in WT, Kir6.2 KO, Ins1Cre/0;Kir6.2flox/+, Ins1Cre/0;Kir6.2flox/flox, NestinCre/0;Kir6.2flox/+, and NestinCre/0;Kir6.2flox/flox mice after 24-hour fasting (labeled: “before”) and after 30-minute re-feeding (labeled: “after”). These datasets are used for **Figure 4D**.

**A**

**800**

**Blood glucose (mg/dl)**

**600**

Control (Before) Control (After 2 wks) Control (After 3 wks) Control (After 4 wks) Control (After 6 wks) Control (After 8 wks)

Rescued (Before) Rescued (After 2 wks) Rescued (After 3 wks) Rescued (After 4 wks) Rescued (After 6 wks) Rescued (After 8 wks)

**400**

**200**

**0**

**0 30 60 90 120 150**

# Time (min)

**B**


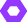

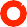

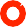

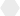

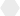

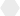

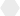

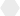

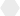

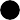

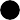


**800**

**Blood glucose (mg/dl)**

**600**

**400**

**C**

**800**

**Blood glucose (mg/dl)**

**600**

**400**

Before After 2 wks

After 3 wks

After 4 wks

After 6 wks

After 8 wks

**200 200**

**0**

**0 30 60 90 120 150**

**Time (min)**

**0**

**0 30 60 90 120 150**

**Time (min)**

Resting


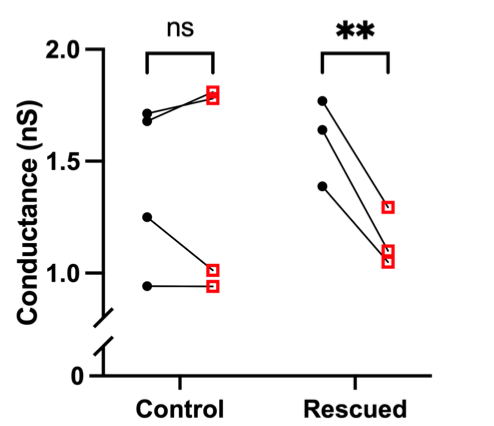


**F**

Glibenclamide 10 µM

**D E**

**400** ✱✱


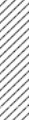

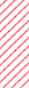

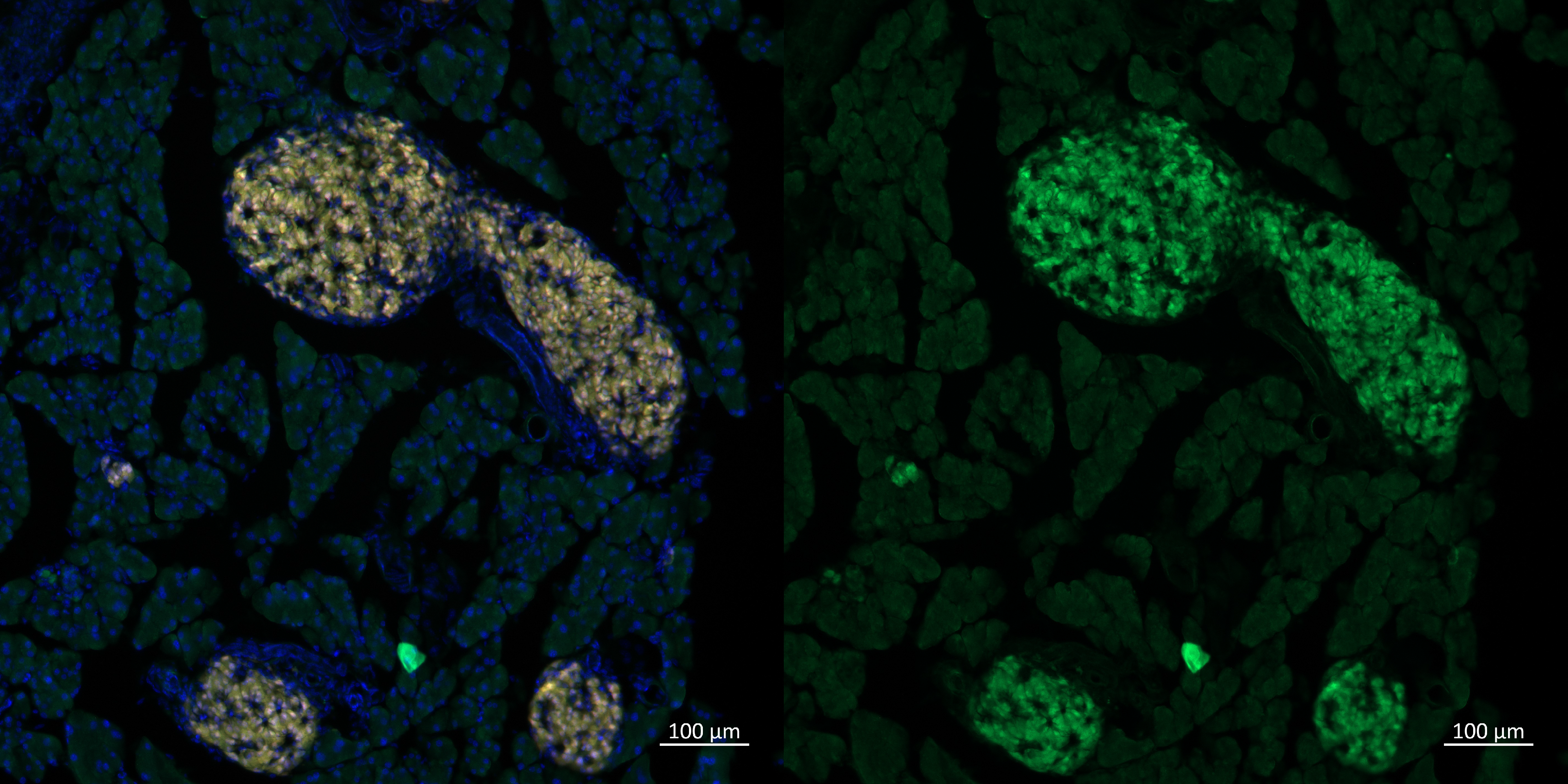

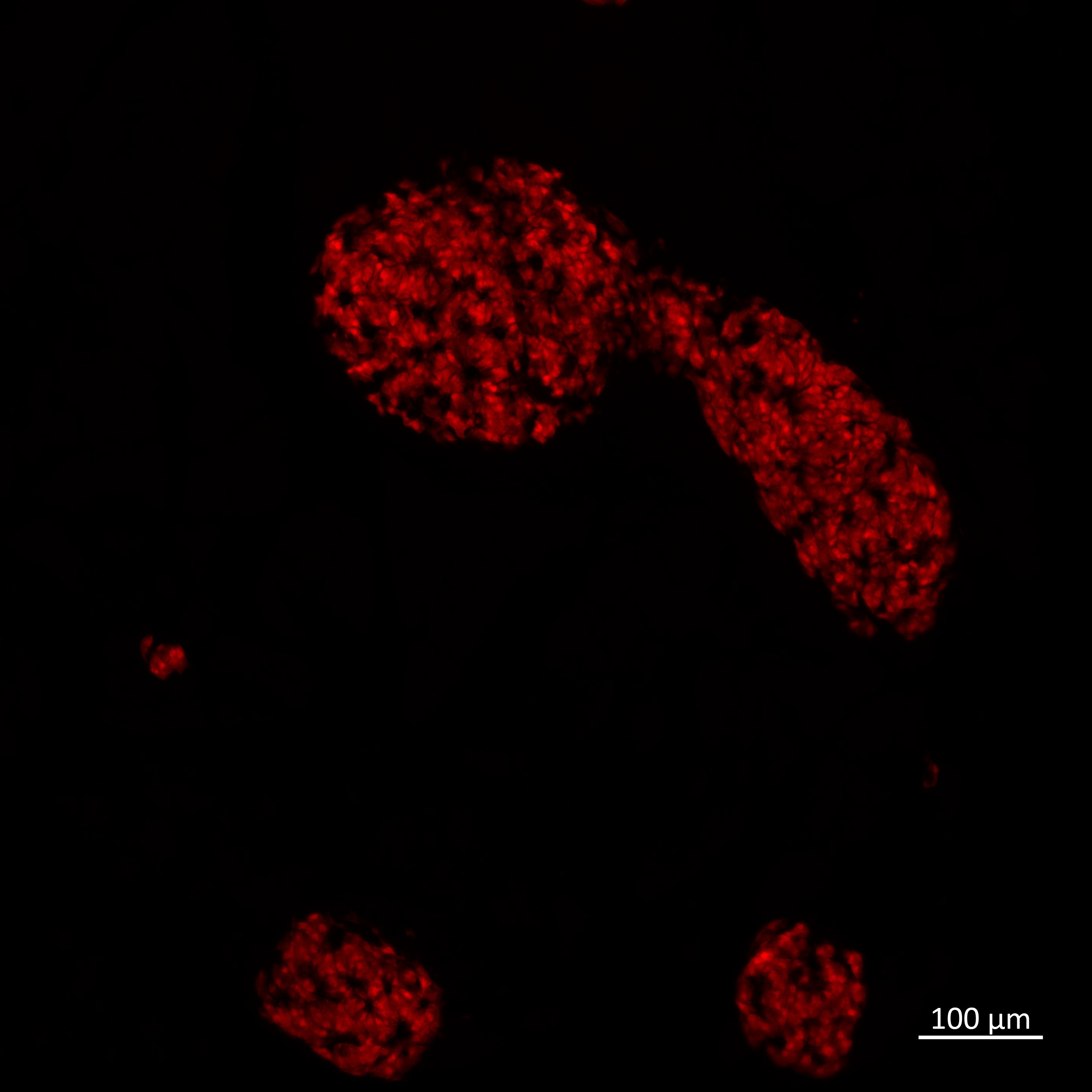


**300**

**Blood glucose (mg/dl)**

**200**

**100**

**0**

## Merged GFP tdTomato

**Supplementary Figure 8: Restoration of functional pancreatic K_ATP_ channels rescues glucose homeostasis.**

(legend on next page)

**Supplementary Figure 8: Restoration of functional pancreatic K_ATP_ channels rescues glucose homeostasis. A:** All of the IPGTT blood glucose traces in control and rescued mice before surgery and 2, 3, 4, 6, and 8 weeks post-surgery. IPGTT traces from **control** mice are labeled in **grayscale** colors, from black (before) to white (8 weeks post-surgery); IPGTT traces from **rescued** mice are labeled in **rainbow** colors, red (before), orange (2 weeks post-surgery), yellow (3 weeks post-surgery), green (4 weeks post-surgery), blue (6 weeks post-surgery), and purple (8 weeks post-surgery). **B**–**C:** Split figures from **A**. Blood glucose levels during IPGTT of control (**B**) and rescued mice (**C**) before and 2, 3, 4, 6, and 8 weeks post-surgery. **D:** Basal blood glucose levels after a 6-hour fasting duration of control and rescued mice before and 8 weeks after surgery. **E:** Fluorescent images of Ins1Cre/0;Ai14+;Kir6.2 KO pancreatic islets that have been successfully infected with AAV9-GFP. Green, EGFP; red, tdTomato (Ins1+); blue, DAPI. Scale bars, 20 μm. **F:** conductance before and after 10 μM glibenclamide administration of control (*left*) and rescued (*right*) Ins1Cre/0;Kir6.2 KO pancreatic β cells indicates successful functional K_ATP_ channel restoration. Control: *n* = 9 (**A**–**D**), 4 cells (**F**); Rescued: *n* = 10 (**A**–**D**), 3 cells (**F**). Statistical comparisons were made by two-way ANOVA with Tukey’s correction (**A**–**C**), one-way ANOVA with Tukey’s correction followed by multiple comparisons (**D**), and two-way ANOVA followed by Fisher’s least significant difference (LSD) test (**F**). Data are represented as mean ± SEM. ***P* < 0.01, *****P* < 0.0001, ns: not significant.

**Alt text:** Graphs depicting that glucose tolerance is only rescued upon restoration of functional pancreatic K_ATP_ channels in mice previously lacking both pancreatic and neuronal functional K_ATP_ channels are labeled from **A** to **D**, and figures with fluorescent labeling to show successful vehicle (AAV-GFP) viral administration in the pancreatic islets are included in **E**. Graph showing that K_ATP_-dependent K+ conductance is reinstated upon functional K_ATP_ channel restoration to pancreatic β cells is labeled in **F**, in which the administration of K_ATP_ channel blocker glibenclamide significantly decreases the conductance.

**Kir6.2 KO**

**Wild-type**


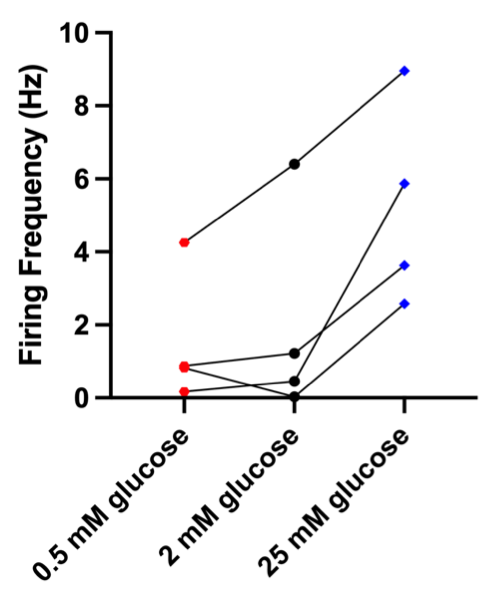


**D**

**A**

**Glucose-excitatory**

**E**

**B**

**Glucose-inhibitory**

**F**

**C**

**Non-glucoresponsive**

## Supplementary Figure 9: Firing frequencies of individual VMH neurons of different glucose responsiveness.

**A**–**F:** Firing frequencies of WT GE (**A**), GI (**B**), non-GR (**C**), Kir6.2 KO GE (**D**), GI (**E**)and non-GR (**F**) VMH spontaneous firing neurons.

WT GE: *n* = 3 (**A**); WT GI: *n* = 2 (**B**); WT non-GR: *n* = 6 (**C**); Kir6.2 KO GE:

*n* = 4 (**D**), Kir6.2 KO GI: *n* = 3 (**E**); Kir6.2 KO non-GR: *n* = 5 (**F**).

**Alt text:** Graphs depicting firing frequencies of, from left to right, glucose-excitatory, glucose- inhibitory, and non-glucoresponsive individual ventromedial hypothalamic neurons with (*top*) or without (*bottom*) functional K_ATP_ channels under low (0.5 mM), intermediate (2 mM), and high (25 mM) glucose concentrations.
